# Transposase‐Assisted Donor Tethering Boosts Large‐Fragment HDR in Plants

**DOI:** 10.1002/advs.75565

**Published:** 2026-05-06

**Authors:** Sha Wei, Kai Zhang, Shuke Deng, Jinjie Chen, Xiaomei Huang, Jingfei Guo, Yuqing Wu, Yingjie Guo, Zhen Liang

**Affiliations:** ^1^ School of Life Science Shanxi University Taiyuan China; ^2^ Institute of Big Data Science and Industry Shanxi University Taiyuan China

**Keywords:** CRISPR/Cas9, homology‐directed repair, plant, transposase

## Abstract

Precise insertion of large DNA fragments by homology‐directed repair (HDR) remains inefficient and poorly reproducible in plants, largely due to limited donor availability at double‐strand break sites. Here, we develop a transposase‐assisted donor tethering strategy that improves the reliability of HDR‐mediated large‐fragment insertion. By fusing Cas9 to an integration‐defective *piggyBac* variant that retains sequence‐specific DNA‐binding activity, donor templates are physically co‐localized with Cas9‐induced breaks. When combined with a transcription‐coupled donor and a repair‐pathway‐biased Cas9 variant, this system enhances the frequency of accurate large‐fragment insertions. Using this approach, we achieved efficient and precise kilobase‐scale targeted gene insertions across multiple loci in both dicot and monocot species. These findings establish donor tethering as an effective strategy to improve plant HDR efficiency and provide a general framework for precise large‐fragment genome insertion.

## Graphical abstract

A transposase‐assisted donor tethering strategy is developed to enhance homology‐directed repair in plants. By recruiting donor DNA to double‐strand breaks and synergizing with repair pathway reprogramming and transcription‐coupled donor design, this system markedly improves large‐fragment targeted insertion efficiency, providing a robust platform for precise genome engineering and molecular breeding.

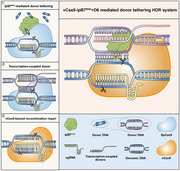

## 1 Introduction

Genome editing has become a foundational technology for functional genomics and genetic manipulation across diverse organisms [1, 2]. Current CRISPR‐based systems enable a wide range of genome modifications. Nucleases such as Cas9 and Cas12, as well as other programmable RNA‐guided nucleases, function by introducing DNA double‐strand breaks (DSBs) that are repaired by error‐prone non‐homologous end joining (NHEJ), generating small insertions or deletions that disrupt gene function [3, 4, 5, 6, 7, 8]. By contrast, base editors enable single‐nucleotide substitutions without generating DSBs, while prime editors extend this capability to substitutions, deletions, and small insertions (<100 bp) [9, 10, 11]. Dual‐prime editing systems have recently expanded the scope of genome engineering by enabling larger‐scale DNA modifications through coordinated manipulation of 3′ DNA flaps generated by paired pegRNAs. These systems support deletions, inversions, and programmable DNA duplication at the chromosomal scale [12, 13, 14]. In addition, dual‐prime editing approaches can facilitate targeted insertions, in some cases reaching up to ∼1 kb in mammalian systems, although efficiency decreases with increasing fragment size [15]. Despite these advances, the accurate insertion of large DNA fragments at predefined genomic loci remains a major challenge, particularly in plants. This limitation constrains applications that require precise kilobase‐scale insertions, such as endogenous gene tagging, regulatory element installation, or function‐preserving locus modification.

In principle, homology‐directed repair (HDR) provides a precise route for targeted sequence insertion [16]. However, large‐fragment HDR in plants is highly variable and poorly reproducible, with outcomes that depend strongly on locus, species, and experimental context [17]. Numerous efforts have sought to bias repair toward HDR. Recent work has demonstrated that engineered Cas9 variants can modulate DSB end configurations, thereby influencing DNA repair pathway choice. For example, a recently developed Cas9 variant (vCas9) predominantly generates staggered double‐strand breaks with 5′ overhangs, in contrast to the blunt‐end cleavage pattern typically produced by wild‐type SpCas9. In mammalian cells, vCas9 has been shown to suppress canonical NHEJ and bias repair toward pathways that utilize homologous sequence, including MMEJ and, in the presence of donor templates, HDR [18]. Increasing donor template availability through geminivirus‐derived replicons [19, 20, 21, 22, 23] or transcription‐coupled donor systems [24, 25] has yielded improvements in specific systems. In parallel, donor recruitment strategies that enrich repair templates near DSBs have proven effective in animal cells using diverse molecular tethering approaches [26, 27, 28]. In plants, Cas9‐VirD2 and related fusions have demonstrated donor recruitment effects, but their utility has largely been restricted to small‐scale sequence modifications [29, 30]. Together, these efforts highlight a common bottleneck in plant HDR: the low and stochastic availability of donor templates at DSB sites, which underlies locus dependence and poor reproducibility of large‐fragment insertion.

DNA transposons offer an underexplored mechanism for mobilizing genetic cargo with high efficiency. The *piggyBac* transposon is notable for its strong affinity for defined terminal repeat sequences and its capacity to mobilize large genetic cargos [31]. Importantly, engineered *piggyBac* variants have been developed that retain DNA‐binding activity while lacking integration capacity, raising the possibility that such variants could be repurposed as molecular tethers rather than mobile elements [32]. Leveraging this property provides a strategy to stabilize donor‐DSB co‐localization without introducing transposon‐mediated integration or perturbing endogenous regulatory architecture.

Here, we present a Cas9‐*piggyBac* fusion strategy that enhances HDR‐mediated large‐fragment integration in plants. By coupling Cas9‐induced DSBs with the DNA‐binding activity of an integration‐defective *piggyBac* variant, the fusion protein recruits donor DNA to the cleavage site, thereby bridging DNA breakage and repair. When combined with a vCas9 variant and transcription‐coupled donor, the approach enables efficient, scar‐free integration of kilobase‐scale DNA fragments into endogenous loci. Importantly, this strategy consistently improves HDR efficiencies across multiple loci in both *Nicotiana benthamiana* and *Oryza sativa*, supporting its broad applicability for precise genome engineering. Together, these findings establish a transposase‐assisted donor recruitment framework that addresses long‐standing barriers to precise large‐fragment knock‐ins and provides a versatile platform for precision molecular breeding in plants.

## 2 Results

### 2.1 Donor Tethering Establishes a Functional Foundation for HDR in Plants

**FIGURE 1:**
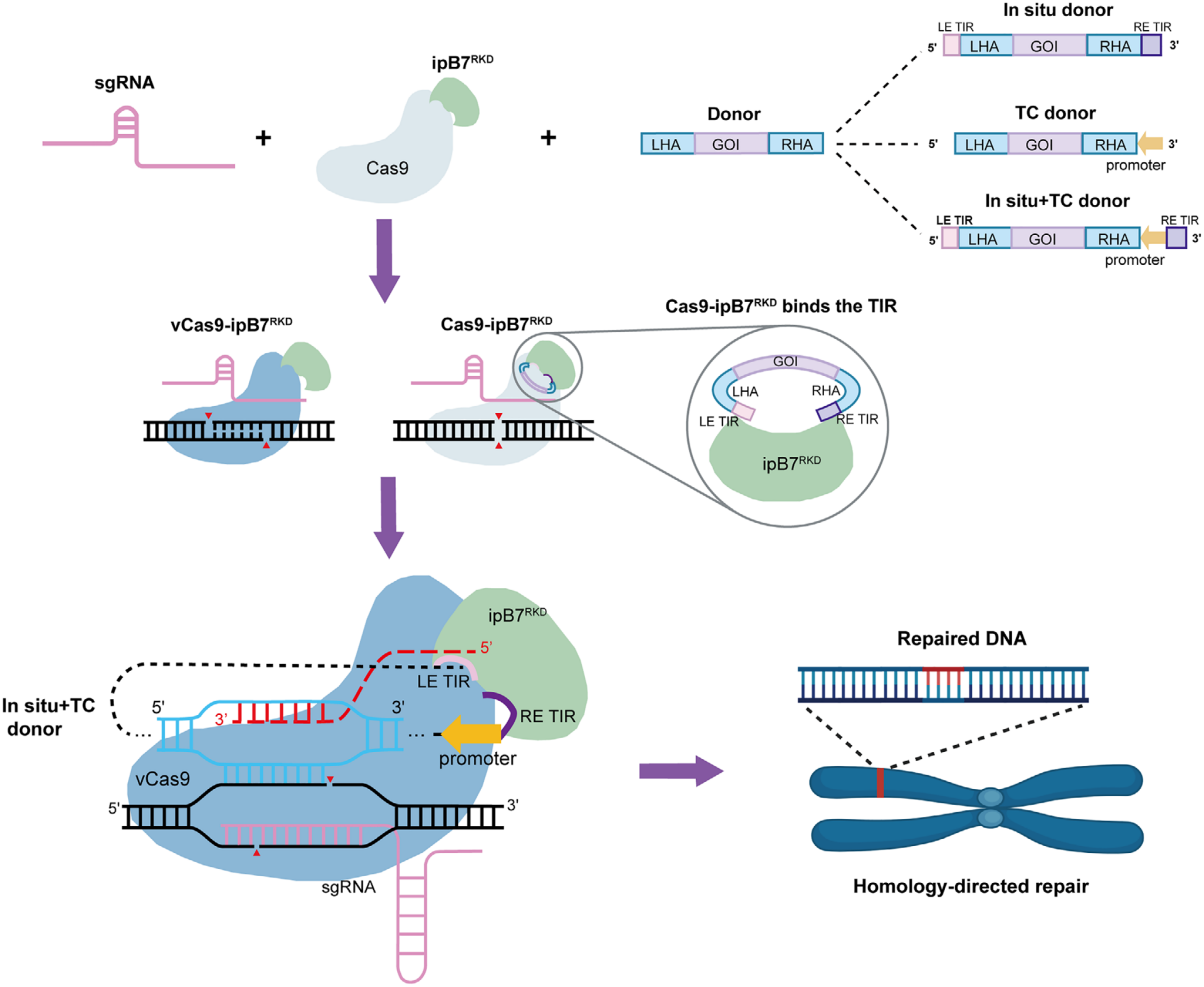
Schematic illustration of the Cas9‐*piggyBac* fusion strategy for enhancing HDR efficiency in plants. An integration‐defective *piggyBac* (PB) variant (ipB7^RKD^) is fused to Cas9 to recruit donor DNA to Cas9‐induced double‐strand breaks (DSBs), increasing local donor concentration and favoring homology‐directed repair (HDR). The donor template carries the gene of interest (GOI) flanked by target‐specific left/right homology arms (LHA/RHA). In situ donor: the template is flanked by PB left/right terminal inverted repeats (LE‐TIR/RE‐TIR) to enable ipB7^RKD^ binding. TC (transcription‐coupled) donor: the template includes an RNA polymerase III promoter to promote donor DNA duplex opening. In situ + TC donor: combines both features. A vCas9 variant biases DSB repair pathway choice toward HDR. Together, the vCas9‐ipB7^RKD^ fusion, sgRNA, and in situ + TC donor form a ternary complex that drives precise, efficient, large‐fragment knock‐in by HDR.

A prerequisite for donor‐tethering‐based HDR is the ability to physically recruit donor DNA to Cas9‐induced double‐strand breaks. DNA transposons such as *piggyBac* (PB) possess intrinsic sequence‐specific DNA‐binding activity that can be repurposed for this purpose. Among engineered PB variants, the excision‐competent but integration‐defective mutant ipB7^R372A/K375A/D450N^ (hereafter referred to as ipB7^RKD^) lacks genomic reintegration capacity while retaining robust cleavage activity at terminal inverted repeat (TIR) sequences (Figure S1) [32]. We therefore hypothesized that ipB7^RKD^ could serve as a molecular tether to localize donor DNA near Cas9‐induced DSBs without conferring autonomous integration activity (Figure 1).

**FIGURE 2:**
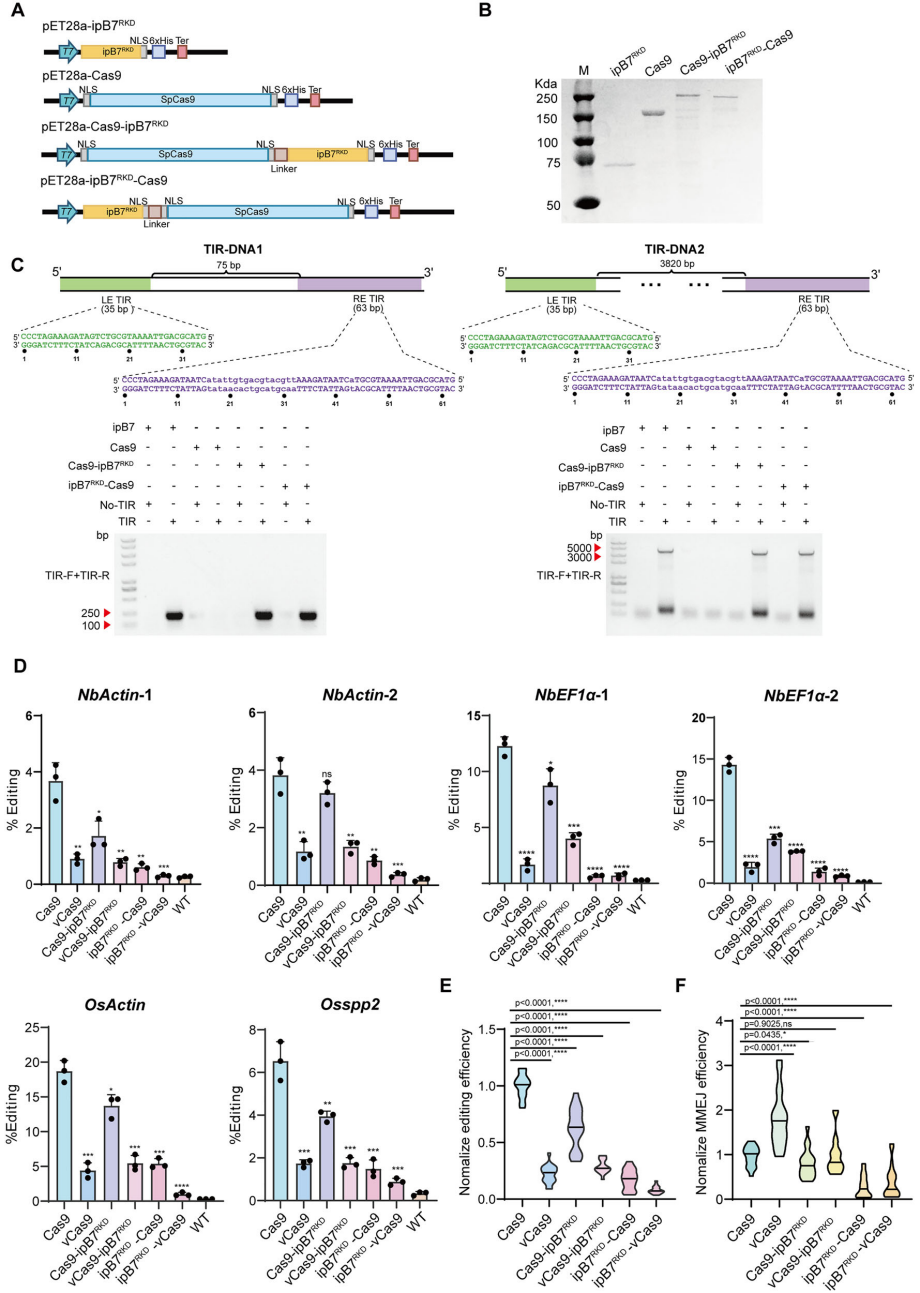
Cas9‐ipB7^RKD^ fusion protein efficiently binds donor templates and cleaves the target loci in plants. (A) Schematic diagrams of prokaryotic expression constructs encoding ipB7^RKD^, Cas9, Cas9‐ipB7^RKD^, and ipB7^RKD^‐Cas9 fusion proteins. (B) Expression and purification of ipB7^RKD^, Cas9, Cas9‐ipB7^RKD^, and ipB7^RKD^‐Cas9 from *E. coli* BL21 (DE3). Proteins purified by Ni‐NTA affinity chromatography were analyzed by SDS‐PAGE, showing bands of the expected sizes (68, 155, 223, and 223 kDa). (C) DNA‐binding assays of ipB7^RKD^, Cas9, Cas9‐ipB7^RKD^, and ipB7^RKD^‐Cas9 with donor templates containing *piggyBac* terminal inverted repeats (TIRs). Each protein was incubated with short (TIR‐DNA1) and long (TIR‐DNA2) DNA fragments carrying TIR sequences. (D) Comparison of the editing efficiency achieved by Cas9, vCas9, Cas9‐ipB7^RKD^, ipB7^RKD^‐Cas9, vCas9‐ipB7^RKD^, and ipB7^RKD^‐vCas9 at four target sites in *Nicotiana benthamiana* and at 2 target sites in rice. Editing efficiencies (mean ± s.e.m.) are based on three independent experiments (n = 3). (E) Overall genome editing efficiencies achieved by Cas9, vCas9, Cas9‐ipB7^RKD^, ipB7^RKD^‐Cas9, vCas9‐ipB7^RKD^, and ipB7^RKD^‐vCas9. Editing efficiencies achieved by Cas9 for each target were normalized to 1, and the efficiencies of other constructs for each target were adjusted accordingly. (F) Relative microhomology‐mediated end‐joining (MMEJ) efficiencies normalized to Cas9. *p*‐values were determined using the two‐tailed Student's *t*‐test: ^*^ *p* < 0.05, ^**^ *p* < 0.01, ^***^ *p* < 0.001.

To determine whether ipB7^RKD^ retains donor‐binding capacity when fused to Cas9, we generated fusion proteins with ipB7^RKD^ placed at either the N‐ or C‐terminus of Cas9 and expressed them alongside ipB7^RKD^ alone and Cas9 controls (Figure 2A,B). In vitro DNA‐binding assays revealed that both Cas9‐ipB7^RKD^ and ipB7^RKD^‐Cas9 efficiently bound donor templates flanked by *piggyBac* left‐end (LE) and right‐end (RE) TIRs [33] (Table S1), whereas no detectable interaction was observed with donor templates lacking TIRs (Figure 2C and Figure S2). This TIR‐dependent binding behavior was preserved when a 3.8‐kb DNA fragment was inserted between the LE and RE TIRs, indicating that ipB7^RKD^‐mediated donor association is not limited by donor size within the kilobase range relevant to plant genome engineering (Figure 2C and Figure S3).

Because effective donor tethering must be compatible with Cas9‐mediated DNA cleavage and subsequent recombination, we next assessed whether fusion of ipB7^RKD^ affects nuclease activity at endogenous genomic targets. Genome editing efficiencies of Cas9‐ipB7^RKD^, ipB7^RKD^‐Cas9, and their corresponding vCas9 variants were evaluated at four loci in *Nicotiana benthamiana* and two loci in *Oryza sativa* using transient agroinfiltration and protoplast transfection assays, respectively (Tables S2 and S3). While C‐terminal fusion of ipB7^RKD^ to Cas9 resulted in a moderate reduction in overall cleavage efficiency compared with Cas9 alone, N‐terminal fusion caused a more pronounced decrease (Figure 2D,E). Based on these results, Cas9‐ipB7^RKD^ was selected for subsequent HDR experiments as a balance between donor tethering capacity and nuclease activity.

In addition to donor proximity, modulation of DNA repair pathway choice has been reported to influence HDR outcomes. The vCas9 variant exhibited reduced overall cleavage activity in plants but generated a higher proportion of microhomology‐mediated end joining (MMEJ) events relative to Cas9 (Figure 2F and Figures S4–S7). Fusion of ipB7^RKD^ to vCas9 retained this repair bias, indicating that donor tethering and repair‐pathway modulation are compatible and can be integrated within a single editing system. Collectively, these observations establish that ipB7^RKD^ and Cas9 fusion proteins retain sequence‐specific donor‐binding activity, maintain sufficient nuclease function, and support recombination‐associated repair pathways, providing a functional foundation for donor‐tethered HDR in plants.

### 2.2 Donor‐DSB Co‐Localization Systematically Modulates HDR Outcomes in Plants

**FIGURE 3:**
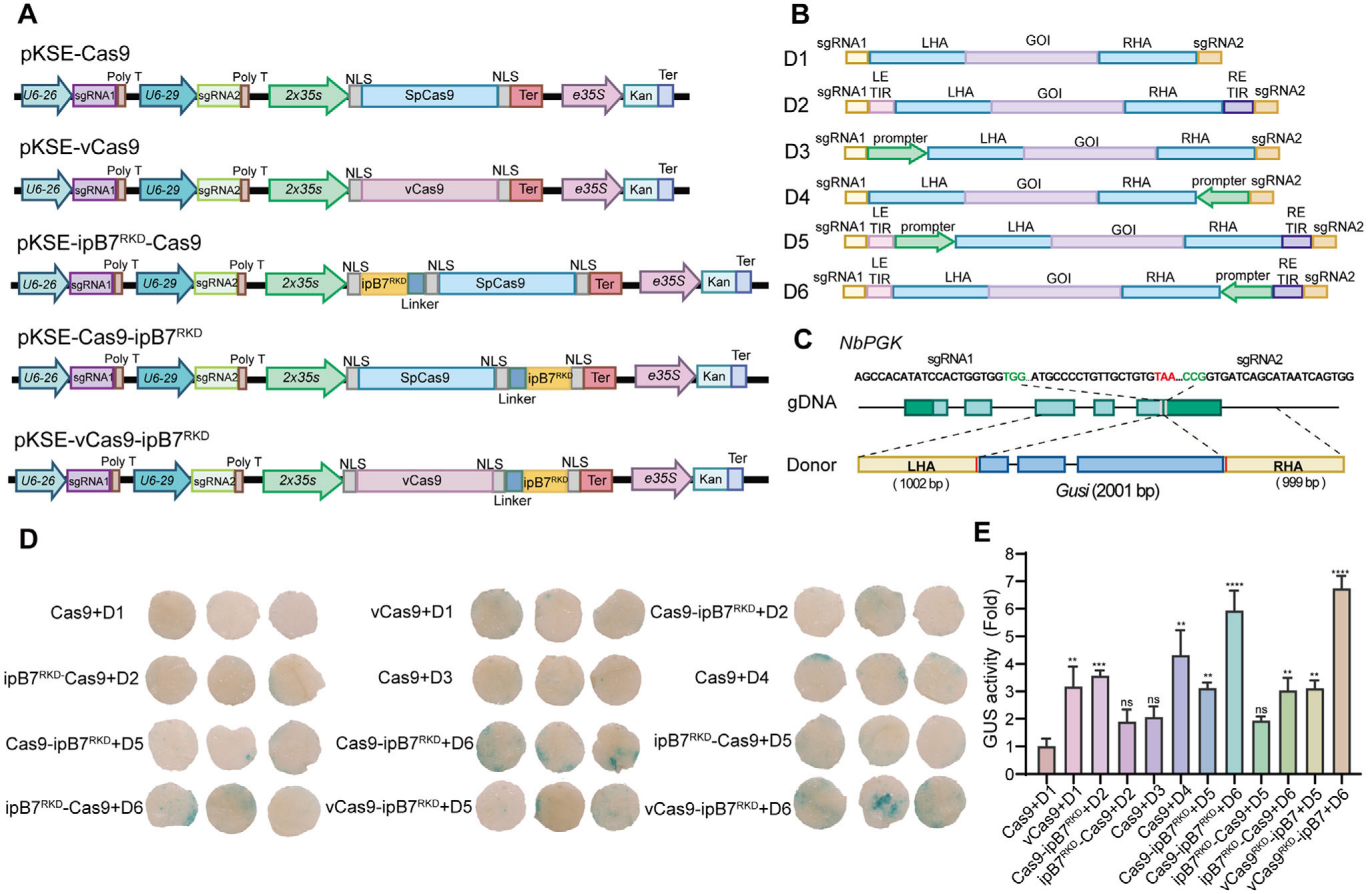
Cas9‐ipB7^RKD^ fusion enhances targeted GUS integration at endogenous loci in *N. benthamiana*. (A) Schematic diagrams of binary gene‐editing vectors expressing two sgRNAs and encoding Cas9, vCas9, ipB7^RKD^‐Cas9, Cas9‐ipB7^RKD^, and vCas9‐ipB7^RKD^ for dicots. (B) Design of donor templates used to evaluate the in situ recruitment and transcription‐coupled (TC) effects on HDR efficiency. Each donor is flanked by two sgRNA sites for release from T‐DNA. D2 contains LE/RE TIRs (in situ donor); D3 and D4 include a promoter at either the 5′ or 3′ end (TC donors); D5 and D6 combine both features (in situ + TC donors). (C) Target design for in‐frame *GUSi* insertion at the endogenous *NbPGK* locus. UTRs and exons are shown in dark and light green, 1‐kb homology arms (HAs) in yellow, and the *GUSi* in blue. The sgRNA target site is located near the stop codon. PAM sequences are labeled in green, and the stop codon in magenta. To prevent re‐cleavage after integration, synonymous base substitutions were introduced into the sgRNA target sites within the homology arms and indicated by magenta. (D) GUS‐stained leaf discs, harvested at 2 dpi from corresponding inoculation spots. Blue spots indicate cells with targeted insertions. (E) Quantification of GUS activity from leaf extracts collected two days after infiltration. GUS activity obtained with Cas9‐D1 was normalized to 1, and relative fold changes were calculated for other treatments. Data represent mean ± s.e.m. from three independent experiments (n = 3). *p*‐values were determined using the two‐tailed Student's *t*‐test: ^*^ *p* < 0.05, ^**^ *p* < 0.01, ^***^ *p* < 0.001.

Having established compatibility between donor binding, nuclease activity, and recombination‐associated repair, we next examined whether physically co‐localizing donor DNA with Cas9‐induced DSBs alters HDR behavior in plant cells. To dissect the contribution of donor tethering and its interaction with other HDR‐enhancing modules, we selected Cas9‐ipB7^RKD^, vCas9‐ipB7^RKD^, and ipB7^RKD^‐Cas9, together with Cas9 and vCas9 controls, based on their nuclease activity and repair‐pathway profiles (Figure 3A). Six modular donor configurations (D1‐D6) were designed to independently or combinatorially assess the effects of *piggyBac*‐mediated donor tethering, transcription‐coupled donor activation, and repair‐pathway bias (Figure 3B).

Using a transient agroinfiltration system in *Nicotiana benthamiana*, we targeted the endogenous *NbPGK* locus for insertion of a 2‐kb intron‐containing *GUSi* reporter immediately upstream of the stop codon. Two sgRNAs flanking the stop codon were designed to induce DSBs in the genome and simultaneously release the donor template (Figure 3C). Relative to the basal Cas9‐D1 system, introduction of donor tethering alone (Cas9‐ipB7^RKD^ with D2) increased HDR frequency by approximately 3.6‐fold, supporting that physical donor‐DSB co‐localization enhances homologous recombination (Figure 3D,E). To further examine whether donor tethering enhances the physical association between donor DNA and Cas9 complexes in vivo, we performed chromatin immunoprecipitation followed by quantitative PCR (ChIP‐qPCR) in *Nicotiana benthamiana* leaves transiently expressing selected configurations. The ChIP signals were quantified using the percentage input method [34]. Compared with the Cas9‐D1 control, both Cas9‐ipB7^RKD^ with D2 and vCas9‐ipB7^RKD^ with D6 exhibited markedly increased ChIP signals, with approximately 7.2 and 5.7 fold enrichment, respectively (Figure S8 and Table S4). These results indicate enhanced association between donor DNA and Cas9‐ipB7^RKD^ fusion proteins in vivo. By contrast, N‐terminal fusion of ipB7^RKD^ to Cas9 did not improve HDR, consistent with its reduced cleavage activity. Transcription‐coupled donor activation further modulated HDR outcomes in a directional manner: placement of a U6 promoter at the right end of the donor (D4) increased HDR frequency by 4.3 fold, whereas left‐end placement (D3) resulted in no detectable improvement. In parallel, the vCas9 nuclease alone enhanced HDR by 3.2 fold, supporting its role in biasing DSB repair toward recombination‐associated pathways. Combining donor tethering with transcription‐coupled activation produced synergistic effects beyond either factor alone. Cas9‐ipB7^RKD^ paired with the D6 donor yielded a marked enhancement in HDR, and the highest HDR frequency was achieved using vCas9‐ipB7^RKD^ with D6, reaching a 6.7 fold increase over the baseline Cas9‐D1 system (Figure 3E). These results suggest that donor‐DSB co‐localization constitutes a dominant and engineerable determinant of HDR performance in plants, while transcriptional activation and repair‐pathway bias act as complementary modulators. This modular framework establishes a controlled basis for evaluating donor‐tethered HDR across genomic contexts.

### 2.3 Reproducible Scar‐Free Insertion at Endogenous Loci During Stable Plant Regeneration

To determine whether donor tethering supports HDR beyond transient expression and persists during plant regeneration, we evaluated stable HDR events in *Nicotiana benthamiana* using the endogenous phosphoglycerate kinase (*NbPGK*) locus as an initial test case. This locus enables precise insertion immediately upstream of the native stop codon, allowing assessment of targeted integration without altering upstream regulatory elements. Based on the performance hierarchy observed in transient assays, five representative nuclease‐donor configurations were selected to compare individual modules (vCas9 bias, ipB7^RKD^ tethering, or transcription coupling) and their combinations during regeneration.

**FIGURE 4:**
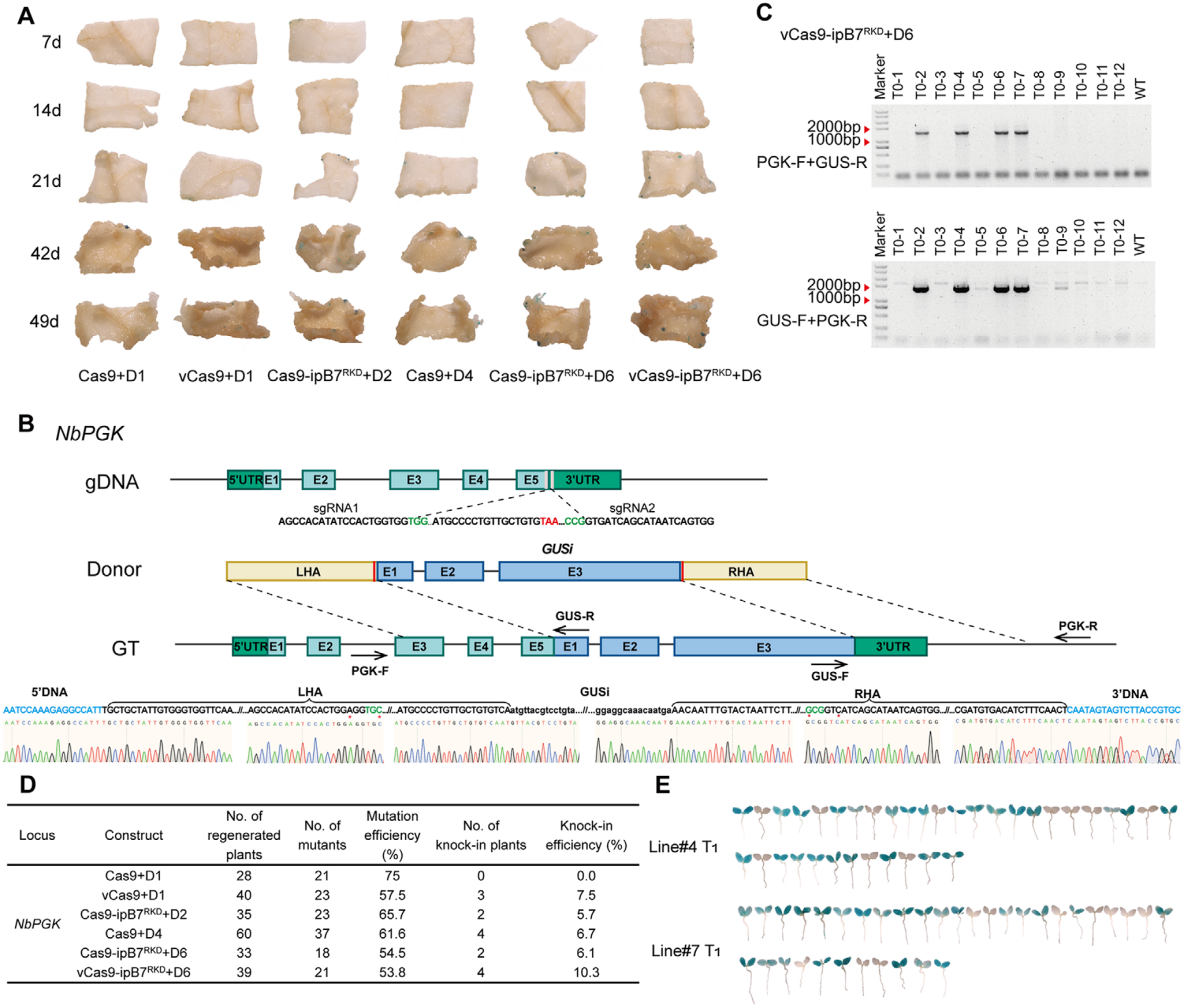
Evaluation of optimized HDR systems for stable integration in *N. benthamiana*. (A) Regenerated *N. benthamiana* tissues following agroinfiltration with six selected HDR strategies. Leaf discs were stained with X‐Gluc at 7, 14, 21, 42, and 49 days post‐inoculation, showing progressive GUS expression. (B) Molecular characterization of gene targeting (GT) events at the *NbPGK* locus. Targeted integration events were detected by junction PCR with primer pairs PGK‐F/GUS‐R and GUS‐F/PGK‐R. Sequencing chromatograms with synonymous substitutions (*) and the intended *GUSi* gene confirmed the perfect insertion. (C) PCR‐based detection of the GT events. The presence of both 5′ (1,758 bp) and 3′ (1,718 bp) junction amplicons indicated targeted *GUSi* integration. (D) Summary of the HDR efficiencies of six knock‐in strategies at the *NbPGK* locus in stable *N. benthamiana* transformants. (E) GUS staining of T_1_ seedlings of two insertion lines generated by vCas9‐ipB7^RKD^+D6, demonstrating stable inheritance of the targeted integration.

Following leaf agroinfiltration and tissue culture regeneration, GUS‐positive calli and shoots were detected for multiple configurations, with the most extensive reporter expression observed in plants generated using the fully integrated vCas9‐ipB7^RKD^ with D6 system (Figure 4A). Genomic PCR and junction sequencing confirmed precise *GUSi* insertion at the *NbPGK* locus, including accurate 5′ and 3′ recombination junctions and the presence of expected synonymous substitutions at sgRNA recognition sites (Figure 4B,C, and Table S5). No targeted insertion was detected in the unoptimized Cas9‐D1 control, whereas configurations incorporating donor tethering, transcriptional activation, or repair‐pathway bias yielded measurable HDR events. The highest knock‐in frequency (10.3%) was observed with vCas9‐ipB7^RKD^ and D6 (Figure 4D). Mutation analysis of T_0_ regenerated plants across different configurations revealed editing efficiencies of 54.5%–75.0% for Cas9‐based systems and 53.8%–57.5% for vCas9‐based systems (Figure 4d), indicating a moderate reduction in mutation efficiency associated with vCas9, consistent with its altered cleavage pattern. These results suggest that the enhanced knock‐in frequencies arise from the coordinated effects of donor tethering, transcription‐coupled donor design, and repair pathway modulation, rather than differences in nuclease activity.

To assess robustness across genomic contexts, the same configurations were evaluated at three additional endogenous loci (*NbTPR*, *NbActin*, and *NbEF1α*). For *NbActin* and *NbEF1α*, a self‐cleaving T2A peptide was used to enable co‐translation of the endogenous protein and the *GUSi* reporter without disrupting native function (Figure S9). At all loci tested, the relative performance hierarchy among editing configurations was preserved (Table 1 and Table S5). These results indicate that donor tethering supports reproducible, function‐preserving HDR across diverse endogenous targets.

**TABLE 1:** Gene targeting frequencies in *N. benthamiana*.

Locus	Construct	No. of regenerated plants	No. of knock‐in plants	Knock‐in efficiency (%)
*NbTPR*	Cas9+D1	38	0	0.0
	vCas9+D1	50	2	4.0
	Cas9‐ipB7^RKD^+D2	30	1	3.3
	Cas9+D4	34	1	2.9
	Cas9‐ipB7^RKD^+D6	56	2	3.5
	vCas9‐ipB7^RKD^+D6	35	3	8.6
*NbActin*	Cas9+D1	38	0	0.0
	vCas9+D1	35	2	5.7
	Cas9‐ipB7^RKD^+D2	40	1	2.5
	Cas9+D4	48	2	4.2
	Cas9‐ipB7^RKD^+D6	42	2	4.8
	vCas9‐ipB7^RKD^+D6	50	5	10.0
*NbEF1α*	Cas9+D1	30	0	0.0
	vCas9+D1	35	2	5.7
	Cas9‐ipB7^RKD^+D2	35	1	2.9
	Cas9+D4	32	1	3.1
	Cas9‐ipB7^RKD^+D6	31	1	3.2
	vCas9‐ipB7^RKD^+D6	38	3	7.9

To evaluate heritability, two independent *NbPGK* knock‐in lines generated using vCas9‐ipB7^RKD^ with D6 were advanced to the T_1_ generation. Junction‐specific PCR revealed Mendelian segregation of the knock‐in alleles, and full‐length PCR identified homozygous insertions in a subset of progeny (Figures S10 and S11). Histochemical GUS staining confirmed these genotypes and produced segregation ratios consistent with Mendelian inheritance in T_1_ plants (Figure 4E). These findings demonstrate that donor‐tethered HDR supports precise and heritable large‐fragment insertion during stable plant regeneration.

### 2.4 *PiggyBac*‐Assisted Donor Tethering Supports Precise HDR Integration in Rice

**FIGURE 5:**
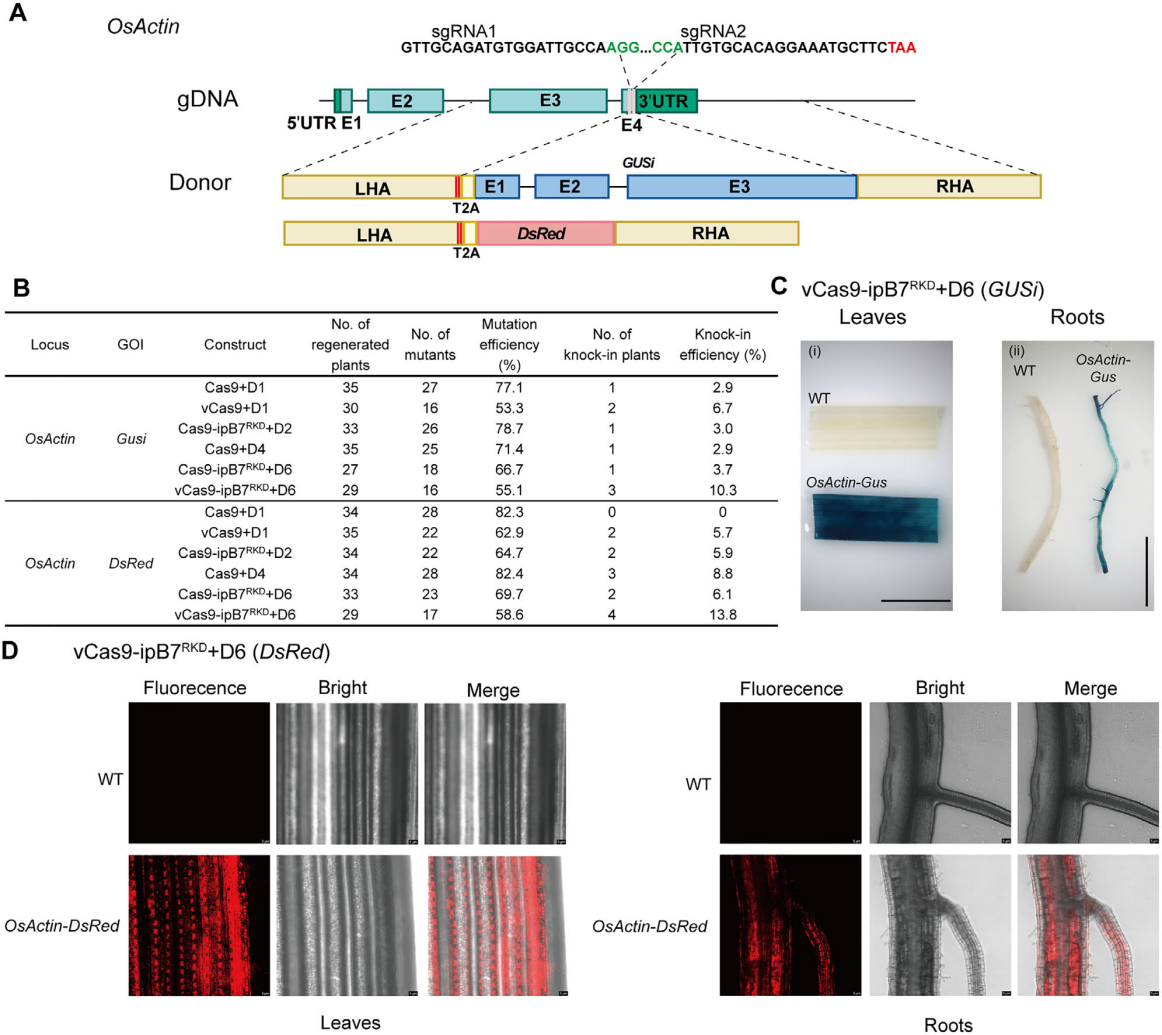
HDR‐mediated knock‐in assay at the *OsActin* locus in rice. (A) Target design for in‐frame *T2A‐GUSi* or *T2A‐DsRed* insertion at the endogenous *OsActin* locus. UTRs and exons are shown in dark and light green, homology arms in yellow, the *GUSi* in blue, and the *DsRed* in red. The sgRNA target site is located near the stop codon. PAM sequences are labeled in green, and the stop codon in magenta. (B) Summary of HDR efficiencies of *T2A‐GUSi* or *T* *2A‐DsRed* insertion using six knock‐in strategies at the *OsActin* locus in stable rice transformants. (C) X‐Gluc staining of *T2A‐GUSi* knock‐in plants. (i) Seedling leaves and (ii) roots from WT and *GUSi* knock‐in line #15. Scale bars, 1 cm. (D) Confocal imaging of DsRed fluorescence in leaves and roots of the *OsActin‐T2A‐DsRed* knock‐in line #27. Scale bar, 5 µm.

To evaluate applicability beyond dicot species and leaf‐based regeneration systems, we examined the donor‐tethering framework in the monocot crop *Oryza sativa* (cv. Nipponbare) using *Agrobacterium*‐mediated callus transformation (Figure S12). Five representative HDR configurations were tested, spanning baseline Cas9 editing, donor tethering, transcription‐coupled donor activation, repair‐pathway bias, and full module integration. The constitutively expressed *OsActin* locus was selected as the target, and two donor templates were designed to assess donor‐size dependence: a 2.0 kb intron‐containing *GUSi* cassette and a 0.7 kb *DsRed* cassette (Figure 5A).

For the insertion of the 2.0 kb *GUSi* cassette, baseline Cas9 editing yielded an HDR efficiency of 2.9%, while vCas9 increased this value to 6.7%. Donor tethering alone, with or without transcriptional activation, produced comparable efficiencies. In contrast, combining donor tethering and transcriptional activation with vCas9 resulted in the highest HDR efficiency of 10.3% (Figure 5B and Table S5). When the shorter *DsRed* donor was used, baseline Cas9 editing failed to yield detectable HDR events. Under these conditions, donor tethering, transcriptional activation, and repair‐pathway bias each contributed to increased HDR efficiencies, with the fully integrated vCas9‐ipB7^RKD^ with D6 configuration achieving the highest efficiency of 13.8% (Figure 5B and Figures S13 and S14). Although absolute HDR efficiencies varied with donor size, the relative performance hierarchy among editing configurations was preserved, indicating predictable and size‐dependent contributions of individual modules. Mutation analysis of T_0_ plants at the *OsActin* locus also showed efficient genome editing across different configurations, despite a moderate reduction in editing efficiency associated with vCas9 and vCas9‐based systems (Figure 5B). Together, these results suggest that the improved knock‐in performance is associated with the integrated vCas9‐ipB7^RKD^ with D6 configuration.

Phenotypic analyses supported the molecular results. GUS staining revealed strong reporter activity in leaves and roots of *GUSi* knock‐in plants, and confocal microscopy detected strong DsRed fluorescence in corresponding tissues (Figure 5C,D), confirming functional expression of the inserted sequences.

### 2.5 High Precision and Genomic Stability of ipB7^RKD^‐Mediated Donor Tethering‐Based HDR

To evaluate genomic precision and stability, whole‐genome sequencing was performed on six independent rice lines generated using the vCas9‐ipB7^RKD^ with D6 configuration (Table S6). These included three *GUSi* and three *DsRed* knock‐in lines at the *OsActin* locus. Genome‐wide analyses confirmed precise insertion of the intended reporter cassettes immediately upstream of the *OsActin* stop codon, with no random donor integrations detected elsewhere in the genome. No *piggyBac*‐derived sequences or transposon‐like insertion footprints were identified, indicating that the integration‐defective ipB7^RKD^ variant did not regain transposition activity when fused to Cas9 (Table S7).

At predicted CRISPR off‐target sites containing three to four mismatches to the sgRNAs, no indels or ectopic donor integrations were detected. Limited indel signals were observed only at sites with one to two mismatches, consistent with the known specificity profile of CRISPR‐based nucleases, and were not associated with donor tethering (Tables S8 and S9). Random T‐DNA insertions were present in T_0_ plants, as expected for *Agrobacterium*‐mediated transformation, but were unrelated to HDR events and can be eliminated through genetic segregation. These results demonstrate that *piggyBac*‐assisted donor tethering enables precise and stable large‐fragment HDR in rice without introducing detectable genome‐wide integration liabilities.

## 3 Discussion

Precise targeted insertion of large DNA fragments remains a persistent challenge in plant genome engineering, largely due to the dominance of non‐homologous end joining (NHEJ) pathways and the intrinsically low activity of homology‐directed repair (HDR). In this study, we developed an integrated HDR framework centered on a *piggyBac*‐derived donor‐tethering module based on the integration‐defective ipB7^RKD^ variant, which physically recruits donor templates to Cas9‐induced double‐strand breaks. This donor‐localization strategy was combined with transcription‐coupled donor activation, which enhances donor accessibility, and vCas9‐mediated modulation of DSB repair, which biases repair toward recombination pathways. Together, these elements address a long‐standing practical limitation of plant HDR by stabilizing donor availability at repair sites, enabling reproducible large‐fragment insertion across plant species without altering the underlying repair machinery. More broadly, these findings suggest that the apparent inefficiency of large‐fragment HDR in plants primarily reflects uncontrolled variability in donor‐DSB encounters, and that enforcing donor localization converts HDR from a stochastic outcome into a reproducible engineering process. However, we note that the number of independent transformation events per locus and treatment group remains relatively limited, which may affect the precision of absolute efficiency estimates. Nevertheless, the relative performance trends among different configurations are consistently observed across multiple genomic loci and both dicot and monocot systems, supporting the robustness of the overall conclusions.

A fundamental barrier to efficient HDR‐mediated gene insertion in plants is the scarcity of donor templates at DSB sites. Unlike animal systems, where donor DNA can often be supplied at high copy number, plant cells impose stringent physical and delivery constraints. *Agrobacterium*‐mediated transformation typically introduces a limited number of T‐DNA copies per cell, resulting in a low probability that an HDR‐competent donor physically encounters a DSB [35]. Strategies such as geminivirus‐based replicons [19, 22] or chemically stabilized donors [36] attempt to overcome this limitation by increasing donor dosage, but often suffer from limited reproducibility, donor‐size constraints, or concerns regarding unintended integration. By contrast, donor tethering increases the effective local concentration of repair templates at the break site without elevating global donor abundance, providing a mechanistically distinct and delivery‐compatible solution to donor scarcity within standard all‐in‐one T‐DNA configurations [37].

Donor recruitment directly addresses the problem of donor scarcity at DSB sites by increasing the effective local concentration of repair templates. Cas9‐VirD2 fusions modestly improved HDR in rice by tethering donors via T‐DNA border sequences, yet this system required phosphorothioate‐protected donors carrying intact border motifs and mainly supported base substitutions [29]. More recently, Cas9‐Rep fusions combined with WDV replicons enabled the medium‐sized insertion of a 519 bp NanoLuc reporter in rice [38]. However, this approach remains limited by the poor reproducibility of WDV replication and by the reduced replication efficiency of geminiviruses as donor size increases, which restricts their use for multi‐kilobase integration. In contrast, *piggyBac* transposases possess a strong affinity for TIRs and have evolved to mobilize large DNA cargoes across diverse organisms. The DNA‐binding capability of the ipB7^RKD^ variant supports its potential use as a molecular tether for donor colocalization. By exploiting this property, our Cas9‐ipB7^RKD^ fusion enables robust co‐localization of donor templates and DSBs using a standard *Agrobacterium*‐delivered all‐in‐one vector without relying on viral replication or chemically stabilized templates.

Many plant engineering applications, including endogenous protein tagging and insertion of reporters or functional modules, demand strict preservation of the endogenous coding sequence and its regulatory context [17]. This emphasis on positional precision distinguishes the present framework from emerging prime editing‐based strategies. Prime editors, such as GRAND and NM‐PE, enable efficient small insertions and epitope tagging, but they are generally constrained by the strict spatial coupling between the PAM site and the intended insertion position [39, 40]. As a result, true stop‐codon‐adjacent tagging remains challenging and often necessitates insertions several codons upstream. By contrast, our HDR system tolerates distances of several nucleotides between the sgRNA cleavage site and the intended insertion site, offering greater flexibility in locus design (Figure S15). To overcome the inherent size limitation of prime editing, recent studies have developed recombinase‐ or integrase‐assisted strategies that couple programmable DNA targeting with site‐specific recombination. Representative systems, including TwinPE [41], PASTE [42], and PrimeRoot [43], enable kilobase‐scale DNA insertions by installing recombination or integration sites at the target loci, followed by recombinase‐ or integrase‐mediated cassette integration. While these approaches significantly expand the editing scope beyond small insertions, they often leave residual recombination or attachment site sequences at the editing junctions, potentially compromising seamless genome engineering. Building on this concept, the PCE system has been adapted for seamless chromosomal modifications in both mammalian cells and plants [44]. In rice, PCE achieved a reported efficiency of 6.2% for targeted GFP insertion. Nonetheless, this strategy typically requires three sequential steps at four targets, including initial integration of recombinase sites, large‐fragment installation, and scar removal at both junctions. Our *piggyBac*‐assisted HDR system achieves large‐fragment integration in a single step using a standard CRISPR‐Cas workflow. The simpler design and flexible positioning of the insertion site make the system particularly attractive for routine tagging and targeted gene integration in plants.

Although the Cas9‐ipB7^RKD^ system substantially improves HDR performance, further optimization remains possible. Recent large‐scale mining of transposase families, together with machine‐learning guided protein engineering, has enabled the development of variants with enhanced activity and altered DNA‐binding properties [45, 46, 47]. These advances suggest that ipB7^RKD^, as well as transposases beyond *piggyBac*, could be further optimized or adapted to expand donor‐tethering strategies. In parallel, improvements in plant regeneration efficiency may further increase the recovery of precise HDR events, particularly in recalcitrant species and elite cultivars.

Overall, this work establishes a *piggyBac*‐assisted HDR platform that enables efficient and precise kilobase‐scale insertion at endogenous loci in both dicot and monocot plants. By converting donor‐DSB proximity into an engineerable parameter, this framework supports function‐preserving, scar‐minimized genome modification in contexts where alteration of promoter architecture or regulatory environment is not tolerated, providing a practical foundation for precision genome engineering in plants.

## 4 Experimental Section

### 4.1 Plant Materials

*Nicotiana benthamiana* plants were grown in a greenhouse under a 16 h light/8 h dark photoperiod, with day/night temperatures of 23°C/18°C, with 50–60% humidity. Plants were cultivated in a 1:1 mixture of potting soil and vermiculite. Four‐ to six‐week‐old plants were used for agroinfiltration experiments.

### 4.2 Rice (*Oryza Sativa* L.)

The Japonica rice variety Nipponbare was used, and incubated at 28°C under a 16 h light/8 h dark photoperiod.

### 4.3 Vector Construction

The codon‐optimized ipB7^RKD^ sequence was synthesized commercially (GenScript, Nanjing, China). The ipB7^RKD^, ipB7^RKD^‐Cas9, and Cas9‐ipB7^RKD^ expression cassettes were assembled into the pET28a (+) backbone by Gibson assembly to generate the corresponding prokaryotic expression vectors (Table S10).

Wild‐type Cas9 was PCR‐amplified from pHUE411 [48] and cloned into pEasy‐Blunt to obtain pEasy‐Cas9. A vCas9 variant was generated by multiple rounds of PCR‐based site‐directed mutagenesis on the pEasy‐Cas9 template. To generate monocot gene‐editing vectors, the Cas9 coding region in pHUE411 was then replaced with vCas9 by *Eco*147I/*Sac*I digestion and ligation, yielding pHUE‐vCas9. To generate fusion constructs, the ipB7^RKD^‐linker module produced by overlap PCR was inserted upstream of the Cas9 or vCas9 coding regions in pHUE411 using *Avr*II/*Sda*I digestion, producing pHUE‐ipB7^RKD^‐Cas9 and pHUE‐ipB7^RKD^‐vCas9. Conversely, the linker‐ipB7^RKD^ fragment was inserted downstream of Cas9 or vCas9 by *Afl*II/*Sac*I digestion to generate pHUE‐Cas9‐ipB7^RKD^ and pHUE‐vCas9‐ipB7^RKD^. To generate dicot gene‐editing vectors, the pKSE401 backbone was digested with *Xba*I and *Sac*I and purified from agarose gels. The DNA fragments encoding vCas9, Cas9‐ipB7^RKD^, vCas9‐ipB7^RKD^, ipB7^RKD^‐Cas9, and ipB7^RKD^‐vCas9 were excised from the corresponding monocot expression constructs using *Xma*JI and *Sac*I. The recovered fragments were subsequently ligated into the *Xba*I/*Sac*I‐digested pKSE401 backbone to obtain the respective dicot editing vectors. Each construct was digested with *Bsa*I to introduce target‐specific sgRNA cassettes, yielding complete genome‐editing vectors (Table S10).

A synthetic LE/RE TIR fragment containing multicloning sites was synthesized and ligated into pEasy‐Blunt to generate pEasy‐TIR for the construction of the D2 donor. For transcription‐coupled donor variants, a U6 promoter was inserted into pEasy‐TIR in the forward orientation using *Eco*RI/*Bam*HI (pEasy‐TIR‐U6; D5 donor) or in the reverse orientation using *Bam*HI/*Hin*dIII (pEasy‐TIR‐antiU6; D6 donor). Left and right homology arms (LHA and RHA) were PCR‐amplified from plant genomic DNA and assembled with the gene‐of‐interest (GOI) fragment into the corresponding donor backbones by seamless cloning. Synonymous substitutions were introduced at sgRNA recognition sites within the LHA or RHA by site‐directed mutagenesis to prevent re‐cleavage after HDR.

Final all‐in‐one HDR vectors were obtained by inserting the completed donor modules into the sgRNA‐containing CRISPR backbones pre‐linearized with *Mss*I, using Gibson assembly (Figure S16 and Table S10).

### 4.4 Protein Purification

The prokaryotic expression plasmids pET28a‐ipB7^RKD^, pET28a‐Cas9, pET28a‐Cas9‐ipB7^RKD^, and pET28a‐ipB7^RKD^‐Cas9 were transformed into *E. coli* BL21 (DE3) for recombinant protein production. The pET28a‐Cas9 construct used in this study was described previously [49]. Transformed cultures were grown to an OD_600_ of 0.6, followed by induction with 0.5 mm IPTG for 18 h at 18°C. Cells were harvested and lysed, and His‐tagged proteins were purified using nickel affinity chromatography (Invitrogen) and dialyzed with storage buffer (20 mm HEPES, pH 7.5, 150 mm KCl, 1 mm DTT, and 3% glycerol). The purity and concentration of Cas9 protein were measured by SDS‐PAGE and Bradford protein assay, respectively.

### 4.5 In Vitro DNA Binding Assay

TIR‐containing DNA fragments were amplified using primers TIR‐F/TIR‐R from pEasy‐TIR or pEasy‐TIR‐Donor, generating a short fragment (TIR‐DNA1, 202 bp) and a long fragment (TIR‐DNA2, 3,952 bp). PCR products were gel‐purified and used as DNA substrates for binding assays. Purified ipB7^RKD^, Cas9, Cas9‐ipB7^RKD^, and ipB7^RKD^‐Cas9 proteins were pre‐incubated with nickel‐NTA agarose resin (Invitrogen) overnight at 4°C to immobilize the His‐tagged proteins. DNA binding reactions (20 µL total volume) contained 8 µL double‐stranded DNA (500 ng), 4 µL 5* DNA‐binding buffer (50 mm Tris‐HCl, 5 mm MgCl_2_, 2.5 mm DTT, 20% Glycerol, 250 mm KCl, 0.015 mm BSA), and 8 µL protein‐resin slurry, and were incubated for 2 h at room temperature. To remove unbound DNA, the resin was washed repeatedly (40 or 45 washes) with 1 mL of 1^*^ DNA‐binding buffer, and wash fractions were collected for PCR analysis (Figures S2 and S3). Bound protein‐DNA complexes were subsequently eluted with elution buffer (25 mm Tris‐HCl, pH 8.0, 500 mm NaCl, 200 mm Imidazole). The eluted material was used as a template for PCR to detect DNA retained by the protein‐resin complex.

### 4.6 Protoplast Transfection

*Oryza sativa* cv. Nipponbare was used for the protoplast experiments. Protoplast isolation and PEG‐mediated transfection were performed as previously described [50]. Approximately 20 µg of plasmid DNA was introduced into the protoplasts using PEG‐mediated transfection. After incubation for 48 h in the dark at 25°C, the protoplasts were collected to extract DNA for subsequent amplicon deep sequencing.

### 4.7 Agrobacterium Transformation of *N. Benthamiana*

T‐DNA vectors were introduced into *Agrobacterium tumefaciens* strain GV3101 using the freeze‐thaw transformation method. A single transformed colony was cultured overnight at 28°C in 5 mL LB medium supplemented with kanamycin (50 mg/L) and rifampicin (25 mg/L). The overnight culture was then inoculated into 20 mL LB containing the same antibiotics and 15 µm acetosyringone, and grown at 28°C with shaking (200 rpm) until the OD_600_ of 0.6. Cells were pelleted and resuspended in infiltration buffer (10 mm MgCl_2_, 10 mm MES, 150 µm acetosyringone, pH 5.6) to a final OD_600_ of 0.8, followed by incubation at room temperature for 3 h to induce virulence. The suspensions were infiltrated into *N. benthamiana* plants using a 1 mL needleless syringe. Leaf discs were collected 36 h post‐infiltration for downstream analyses.

### 4.8 Amplicon Deep Sequencing

Amplicon libraries were generated by a two‐step PCR protocol. In the first round, target loci were amplified using site‐specific primers. In the second round, forward and reverse barcodes were added to the 5′ ends of the primer pairs, yielding amplicons of ∼200 bp suitable for high‐throughput sequencing. Equal amounts of PCR products were mixed as a pool and subjected to commercial sequencing on the Illumina NovaSeq PE150 platform. Three independent biological replicates were analyzed for each target site, and mutation frequencies were quantified using the CRISPR‐Match [51].

### 4.9 GUS Staining and Quantification

GUS staining was performed using standard X‐Gluc assays. Leaf discs or seedling tissues were immersed in GUS staining solution and incubated at 37°C for 24 h. Samples were subsequently cleared in 70% ethanol and imaged using an optical microscope. For quantitative GUS measurements, three infiltrated leaves from the same plant were pooled, and GUS activity was determined using a commercial GUS reporter assay kit according to the manufacturer's instructions.

### 4.10 ChIP‐qPCR Analysis

Plasmids were transiently delivered into *Nicotiana benthamiana* leaves by *Agrobacterium*‐mediated infiltration. After infiltration, plants were kept in the dark for 12 h and then returned to normal growth conditions for an additional 24 h. Approximately 1.5 g of infiltrated leaf tissue was harvested, rinsed twice with sterile water, and cross‐linked in buffer containing 0.4 M sucrose, 10 mm Tris‐HCl (pH 8.0), 5 mm β‐mercaptoethanol, 0.1 mm PMSF, and 2% formaldehyde. Samples were vacuum‐infiltrated for 10 min, followed by quenching with 2 m glycine for 5 min under vacuum. Cross‐linked tissues were ground in liquid nitrogen and resuspended in EB1 buffer (0.4 m sucrose, 10 mm Tris‐HCl, pH 8.0, 5 mm β‐mercaptoethanol, and 0.1 mm PMSF). After incubation on ice for 15 min, the homogenate was centrifuged at 12,000 rpm for 10 min at 4°C. The pellet was sequentially resuspended in EB2 buffer (0.25 m sucrose, 10 mm Tris‐HCl, pH 8.0, 5 mm β‐mercaptoethanol, 0.1 mm PMSF, 1% Triton X‐100, and 10 mm MgCl_2_·6H_2_O) and EB3 buffer (1.7 m sucrose, 10 mm Tris‐HCl, pH 8.0, 5 mm β‐mercaptoethanol, 0.1 mm PMSF, 0.15% Triton X‐100, and 2 mm MgCl_2_·6H_2_O). The resulting nuclear pellet was resuspended in 500 µL lysis buffer (50 mm Tris‐HCl, pH 8.0, 10 mm EDTA, 1% SDS, and 0.1 mm PMSF) and sonicated to shear chromatin (3 s on/3 s off for 15 min). After centrifugation at 16,000 × g for 10 min at 4°C, 10% of the supernatant was reserved as input. For ChIP, 30 µL of Anti‐FLAG M2 agarose beads (Sigma‐Aldrich) were washed twice with PBS and twice with ChIP dilution buffer (1.2 mm EDTA, 16.7 mm Tris‐HCl, pH 8.0, 167 mm NaCl, 1.1% Triton X‐100, and 0.1 mm PMSF), and then incubated with the nucleic acid extract diluted in ChIP dilution buffer. The mixture was incubated overnight at 4°C on a rotating shaker. The immunocomplexes were washed once each with low‐salt wash buffer (1% SDS, 2 mm EDTA, 20 mm Tris‐HCl, pH 8.0, 150 mm NaCl, and 1% Triton X‐100), high‐salt wash buffer (1% SDS, 2 mm EDTA, 20 mm Tris‐HCl, pH 8.0, 500 mm NaCl, and 1% Triton X‐100), and LiCl wash buffer (0.25 m LiCl, 2 mm EDTA, 20 mm Tris‐HCl, pH 8.0, 1% NP‐40, and 1% sodium deoxycholate), followed by two washes with TE buffer (1 mm EDTA and 10 mm Tris‐HCl, pH 8.0). Bound chromatin was eluted twice with 250 µL elution buffer (0.1 M NaHCO_3_ and 1% SDS) at 65°C for 15 min each. Eluates were combined, supplemented with 20 µL 5 M NaCl, and incubated overnight at 65°C with shaking at 550 rpm to reverse cross‐linking. Subsequently, 20 µL 0.25 M EDTA, 20 µL 1 M Tris‐HCl (pH 8.0), and 2 µL proteinase K (20 mg mL^−^^1^) were added, followed by incubation at 45°C for 1.5 h. DNA was purified by phenol:chloroform:isoamyl alcohol extraction (25:24:1), precipitated with ethanol, and dissolved in 30 µL nuclease‐free water. For qPCR, TIR‐specific primers (qTIR‐PGK‐F and qTIR‐PGK‐R) were used to detect enriched DNA fragments in both immunoprecipitated and input samples, with three technical replicates for each sample. The ChIP‐qPCR data were normalized using the Percent Input method (% INPUT) [34]. The ChIP signal was calculated using the following equation: % INPUT = 100 × 2^^((Cq.input‐log2(DF))‐Cq.IP)^, Cq.input and Cq.IP represents the quantification cycle of input and IP samples, and DF represents the dilution factor. Primer sequences for ChIP‐qPCR are listed in Table S4.

### 4.11 *N. Benthamiana* Regeneration

Leaves from 4‐week‐old *N. benthamiana* plants were infiltrated with *Agrobacterium* suspensions carrying the corresponding T‐DNA vectors. For each construct, 3–5 leaves from each of three independent plants were used. Five days after infiltration, leaves were excised and surface‐sterilized in 10% bleach for 20 min, followed by five washes with sterile water. Sterilized leaves were cut into ∼1 × 1 cm pieces and placed on regeneration medium (4.4 g/L MS medium with vitamins, 0.5 g/L MES, 1 mg/L 6‐benzylaminopurine, 0.1 mg/L 1‐naphthaleneacetic acid, 30 g/L sucrose, and 8 g/L agar, pH 5.7) supplemented with kanamycin (100 mg/L) and timentin (200 mg/L). The regenerated shoots were transferred to medium (4.4 g/L MS with vitamins, 0.5 g/L MES, 0.05 mg/L 1‐naphthaleneacetic acid, 30 g/L sucrose, 8 g/L agar, pH 5.7) to obtain the transgenic plants.

### 4.12 Agrobacterium Transformation of Rice

All binary constructs were transformed into *Agrobacterium tumefaciens* strain EHA105 using a freeze/thaw method and selected on LB medium supplemented with kanamycin (50 mg/L) and rifampicin (25 mg/L). Rice transformation was conducted as described previously by Hiei et al. [52]. Mature seeds of the japonica rice variety Nipponbare were used for callus induction. The embryogenic calli were then subjected to infection by *Agrobacterium* and subsequently selected using 50 mg/L hygromycin for 4 weeks to obtain resistant calli. The surviving calli were transferred to regeneration medium containing 50 mg/L hygromycin to regenerate the transgenic plants.

### 4.13 Confocal Microscopy

Fluorescence imaging was performed using a Leica STELLARIS 5 laser scanning confocal microscope (Leica, Germany). DsRed was excited at 552 nm, and emission was collected at 580–615 nm. Leaf and root tissues were mounted on microscope slides in water and imaged immediately. For direct comparison between wild‐type and knock‐in lines, the same exposure settings were used for bright‐field and fluorescence acquisition across corresponding tissue regions.

### 4.14 Whole‐Genome Sequencing Analysis

High‐quality genomic DNA (0.2 µg) from rice was used to construct a sequencing library with the Rapid Plus DNA Lib Prep Kit for Illumina (RK20208). The libraries were sequenced via 2 × 150 bp paired‐end sequencing via Illumina NovaSeq X Plus sequencer. The reference genome of *Oryza sativa* (IRGSP‐1.0) was downloaded from Ensembl Plants (https://www.ensembl.org/). Raw sequencing reads were processed to remove adapter contamination, low‐quality nucleotides, and unrecognized bases (N). The resulting clean reads were aligned to the rice reference genome using the Burrows‐Wheeler Aligner (BWA) software with the mem ‐t 4 ‐k 32 ‐M parameters [53]. PCR duplicates were removed using SAMtools (parameter: rmdup). SNP and indel calling was performed with BCFtools using the command “mpileup ‐q 1 ‐a DP, SP, AD ‐C 50 ‐m 2 ‐F 0.002 ‐L 9999”. The reference genome and the inserted sequence were aligned using BWA to generate BAM files. Structural variations (SVs) were subsequently identified with BreakDancer, from which breakpoints indicative of chromosomal translocations involving the inserted fragment were selected. The corresponding SV results were extracted for further analysis of the inserted sequences. Potential off‐target sites in the rice genome were predicted using Cas‐OFFinder [54].

## Author Contributions

Z.L. designed the projects; S.W., K.Z., S.D., J.C., X.H., J.G., and Y.W. performed most of the experiments; Y.G. analyzed the whole genome sequencing data; S.W. contributed to data analysis and created all figures; Z.L. and S.W. wrote the manuscript, and Z.L. supervised the project.

## Funding

This work was supported by grants from the National Natural Science Foundation of China (32170410 and 62572289), the Fundamental Research Program of Shanxi Province (202403021221020), the Shanxi Scholarship Council of China (2023‐006), the Natural Science Foundation of Shanxi Province for the Excellent Youth (202203021224002), and the Key Project for Core Agricultural Technology Development of Shanxi Province.

## Conflicts of Interest

The authors declare no conflict of interest.

## Supporting information




**Supporting File**: advs75565‐sup‐0001‐SuppMat.docx.

## Data Availability

The authors declare that all data and materials supporting the findings of this study are available from the corresponding author on request. The whole‐genome sequencing data that support the findings of this study have been deposited in the Genome Sequence Archive of the National Genomics Data Center, China National Center for Bioinformation/Beijing Institute of Genomics, Chinese Academy of Sciences (project accession number: PRJCA051718). The core plasmids used in this study, including pHUE‐vCas9‐ipB7^RKD^, pKSE‐vCas9‐ipB7^RKD^, pEasy‐TIR, and pEasy‐TIR‐antiU6, have been deposited in the WeKwikGene plasmid repository (Westlake University; https://wekwikgene.wllsb.edu.cn/) and are publicly available under the following accession numbers: 0002647, 0002648, 0002649 and 0002650.
