# Supplementary material for: Transposase‐Assisted Donor Tethering Boosts Large‐Fragment HDR in Plants
Source: Adv Sci (Weinh). 2026 May 6;13(43):e75565. doi: 10.1002/advs.75565 (PMC13335927; doi:10.1002/advs.75565)
Supplement: Supplementary file 1 — Supporting File: advs75565‐sup‐0001‐SuppMat.docx. [file ADVS-13-e75565-s001.docx]

**Supporting Information**

**Transposase-assisted donor tethering boosts large-fragment HDR in plants**

*Sha Wei*, *Kai Zhang*, *Shuke Deng*, *Jinjie Chen*, *Xiaomei Huang*, *Jingfei Guo*, *Yuqing Wu*, *Yingjie Guo*, *Zhen Liang*^*^

1. Wei, K. Zhang, S. Deng, J. Chen, X. Huang, J. Guo, Y. Guo, Z. Liang

School of Life Science, Shanxi University, Taiyuan 030006, China.

Email: [zliang@sxu.edu.cn](mailto:zliang@sxu.edu.cn)

Y. Guo

Institute of Big Data Science and Industry, Shanxi University, Taiyuan 030006, China.

**Supplementary Figures**


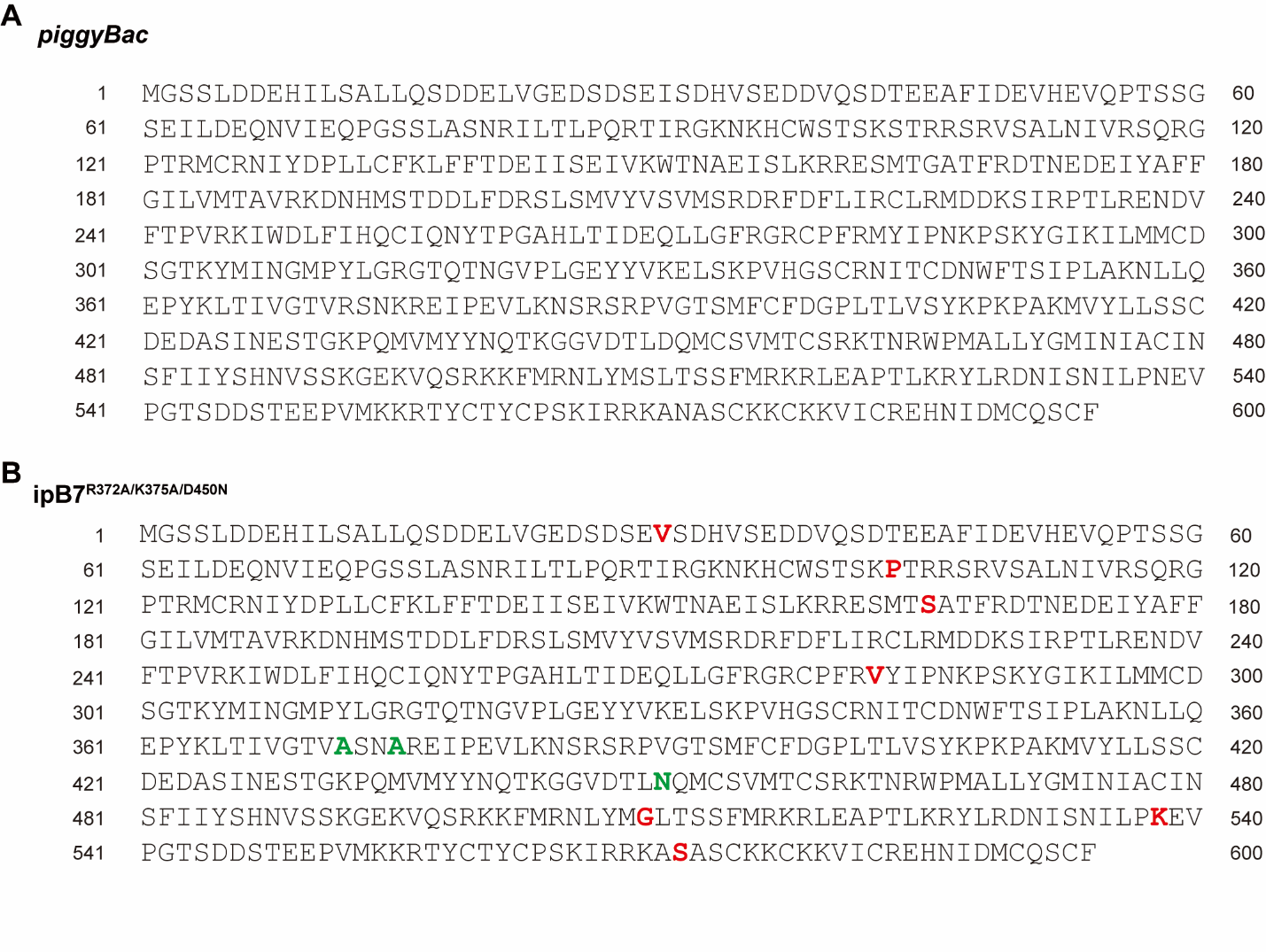


**Figure S1. Amino acid sequences of wild-type *piggyBac* and the ipB7^RKD^ variant.** (A) Amino acid sequence of wild-type piggyBac transposase. (B) Amino acid sequence of the ipB7^RKD^ variant, with the seven substitutions in ipB7 highlighted in red and the R372A/K375A/D450N substitutions highlighted in green.

**
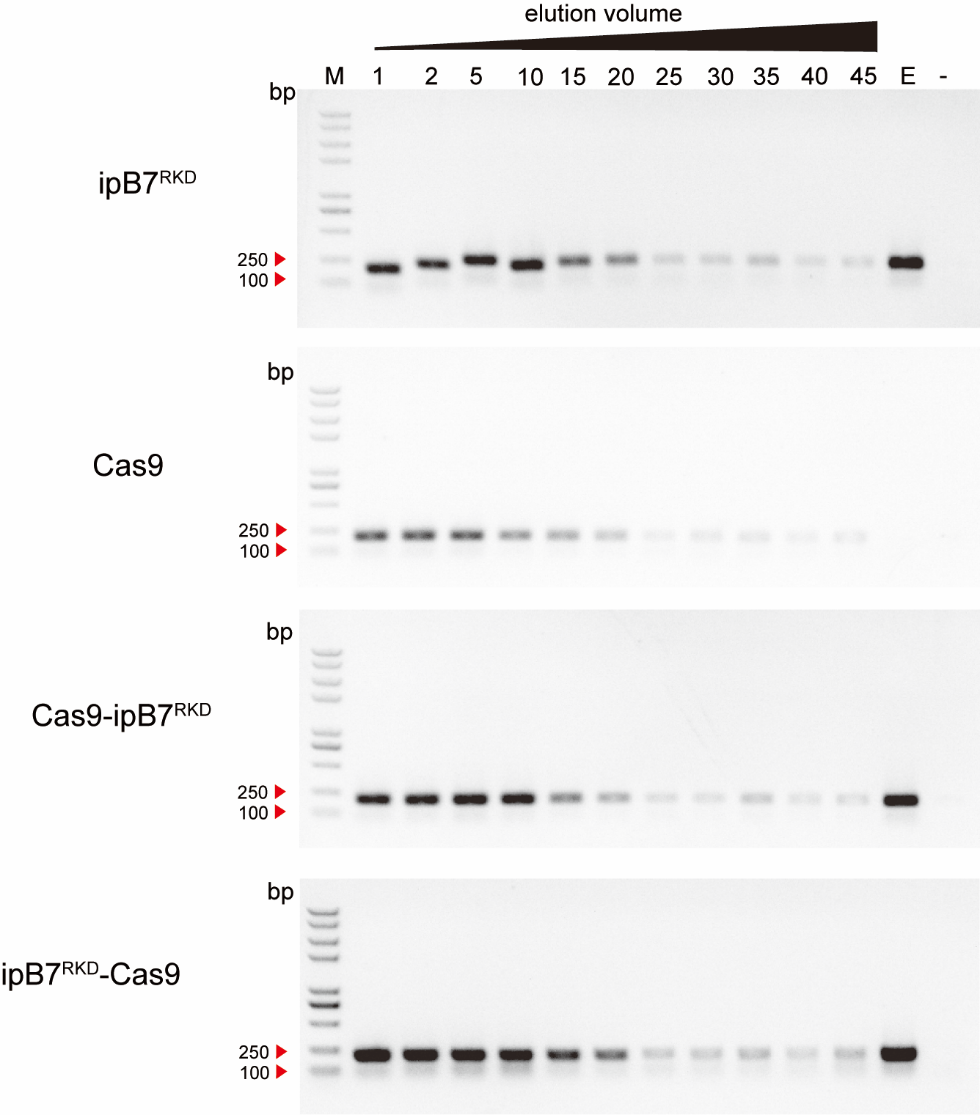
**

**Figure S2.** **DNA-binding assay of *piggyBac*-derived fusion proteins with a short TIR-containing DNA fragment.** Electrophoretic binding assays showing the ability of ipB7^RKD^, Cas9, Cas9-ipB7^RKD^ and ipB7^RKD^-Cas9 to associate with a 202 bp DNA fragment carrying *piggyBac* terminal inverted repeats (TIR-DNA1). Lanes 1-45 represent sequential flow-through fractions containing unbound DNA, whereas lane E indicates DNA retained through protein binding.


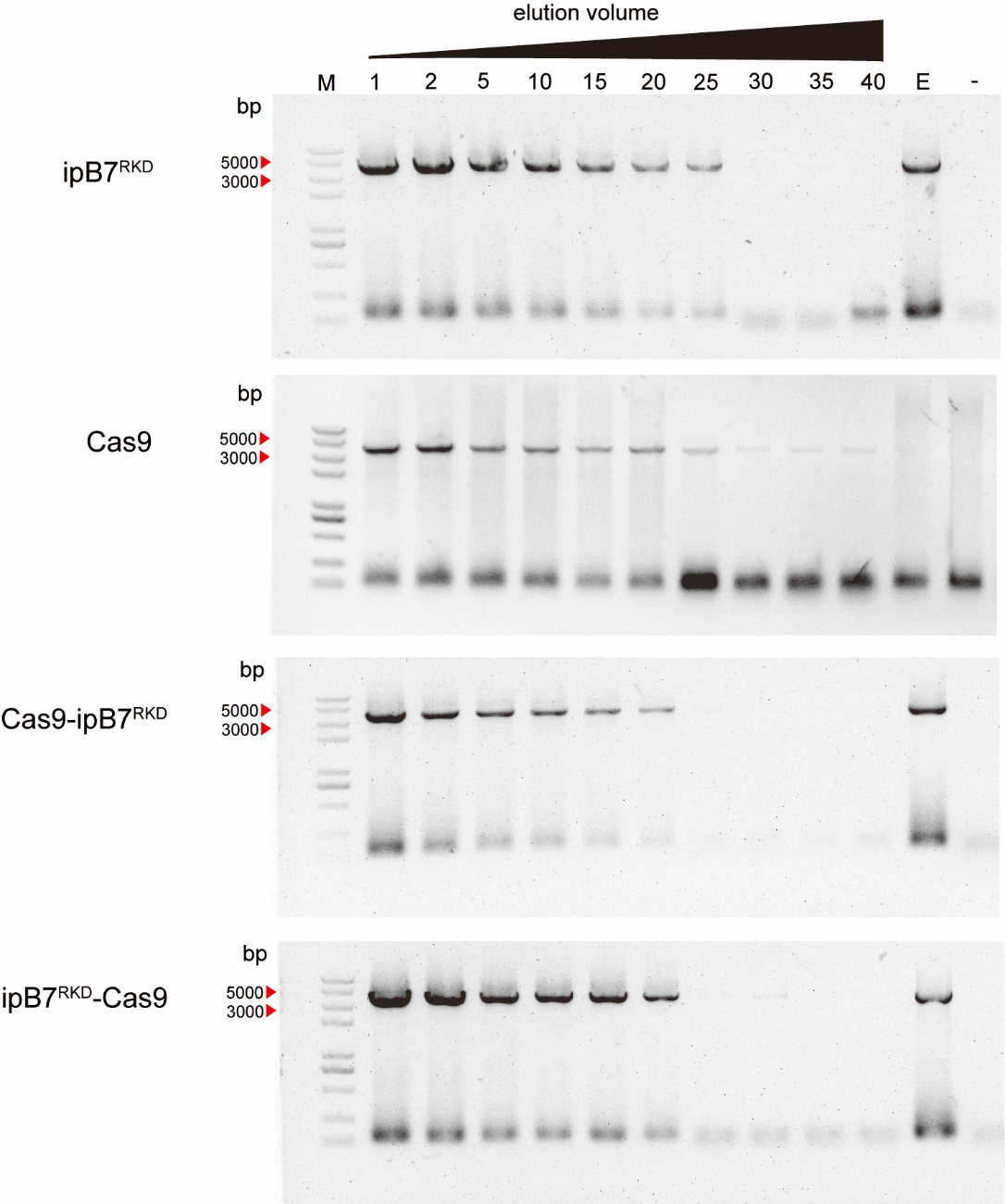


**Figure S3.** **DNA-binding assay of *piggyBac*-derived fusion proteins with a long TIR-containing DNA fragment.** Electrophoretic binding assays showing the ability of ipB7^RKD^, Cas9, Cas9-ipB7^RKD^ and ipB7^RKD^-Cas9 to associate with a 3952 bp DNA fragment carrying *piggyBac* terminal inverted repeats (TIR-DNA2). Lanes 1-40 represent sequential flow-through fractions containing unbound DNA, whereas lane E indicates DNA retained through protein binding.


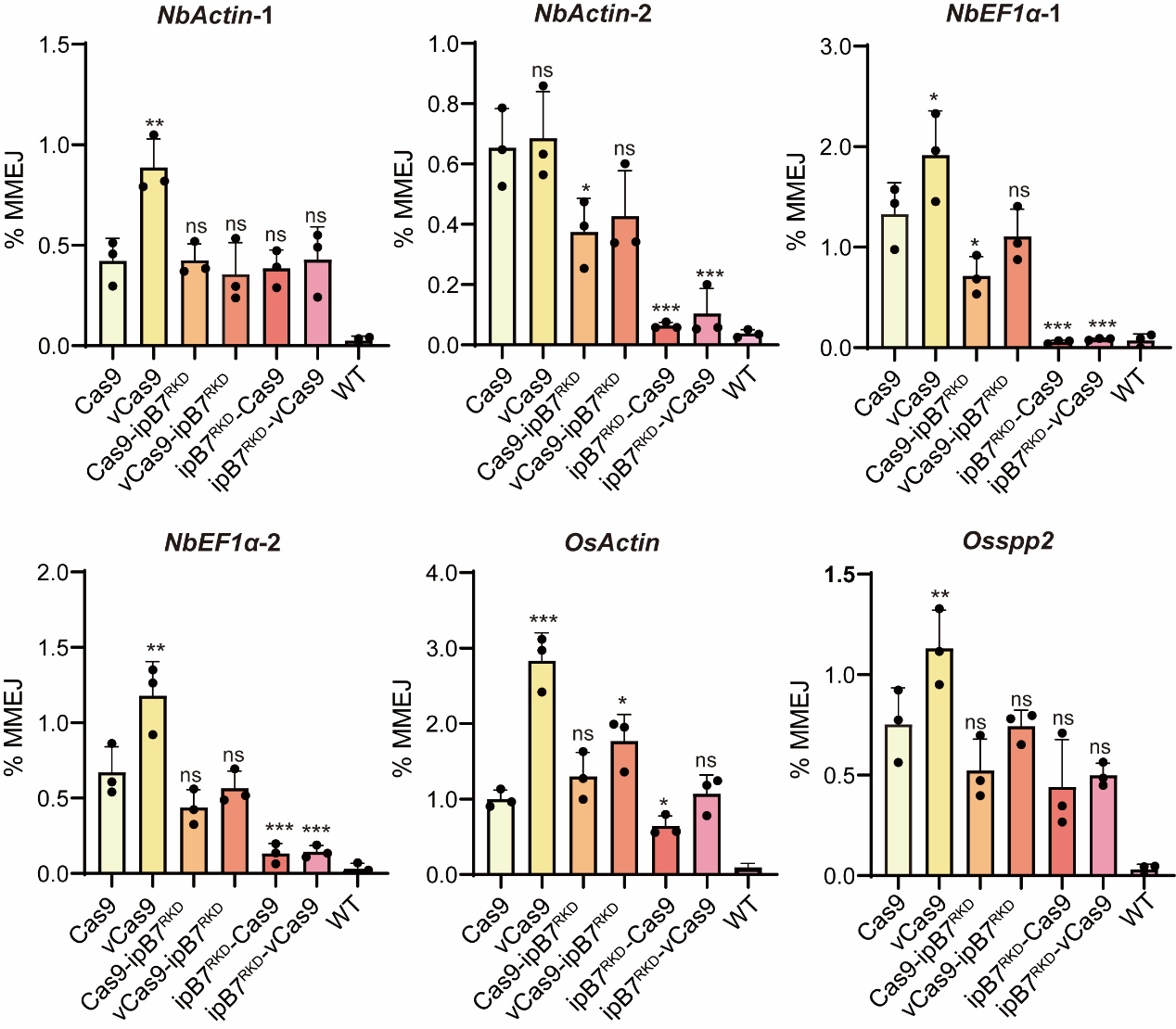


**Figure S4.** **Comparison of the MMEJ efficiency among Cas9 and *piggyBac*-derived fusion variants.** Microhomology-mediated end-joining (MMEJ) efficiencies of Cas9, vCas9, Cas9-ipB7^RKD^, ipB7^RKD^-Cas9, vCas9-ipB7^RKD^, and ipB7^RKD^-vCas9 at 4 target sites in *N. benthamiana* and 2 target sites in rice were analyzed. Editing efficiencies (mean ± s.e.m.) are based on three independent experiments (n = 3). *p*-values were determined using the two-tailed Student’s t-test: **p* < 0.05, ***p* < 0.01, ****p* < 0.001.


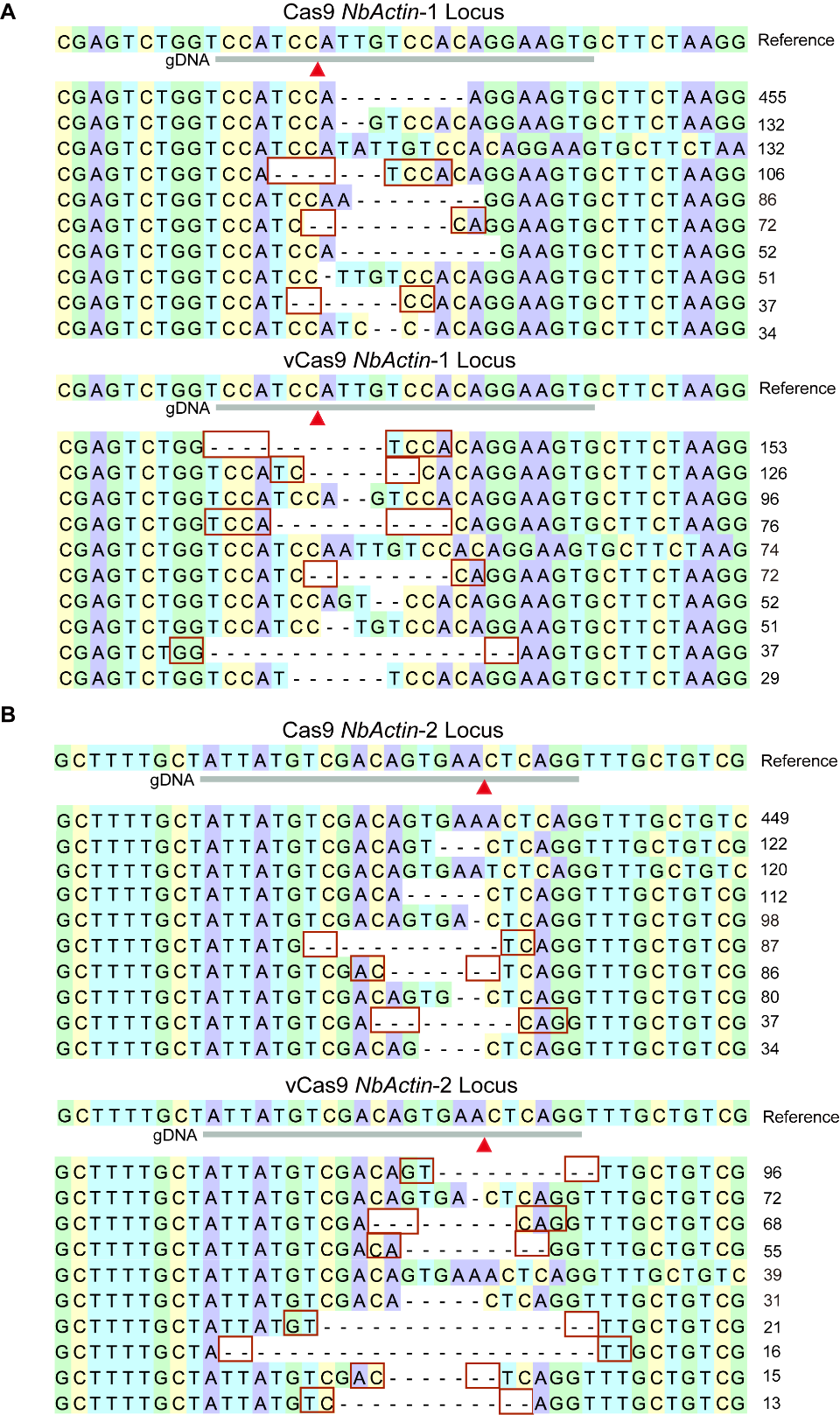


**Figure S5.** **vCas9 suppresses NHEJ and promotes MMEJ at two targets in the *NbActin* locus.** The ten most frequent mutation types generated by Cas9 and vCas9 at the (A) *NbActin*-1 and (B) *NbActin*-2 loci are shown. Deletions at microhomologies are indicated by red boxes. The numbers on the right indicate the read counts for each mutation type.


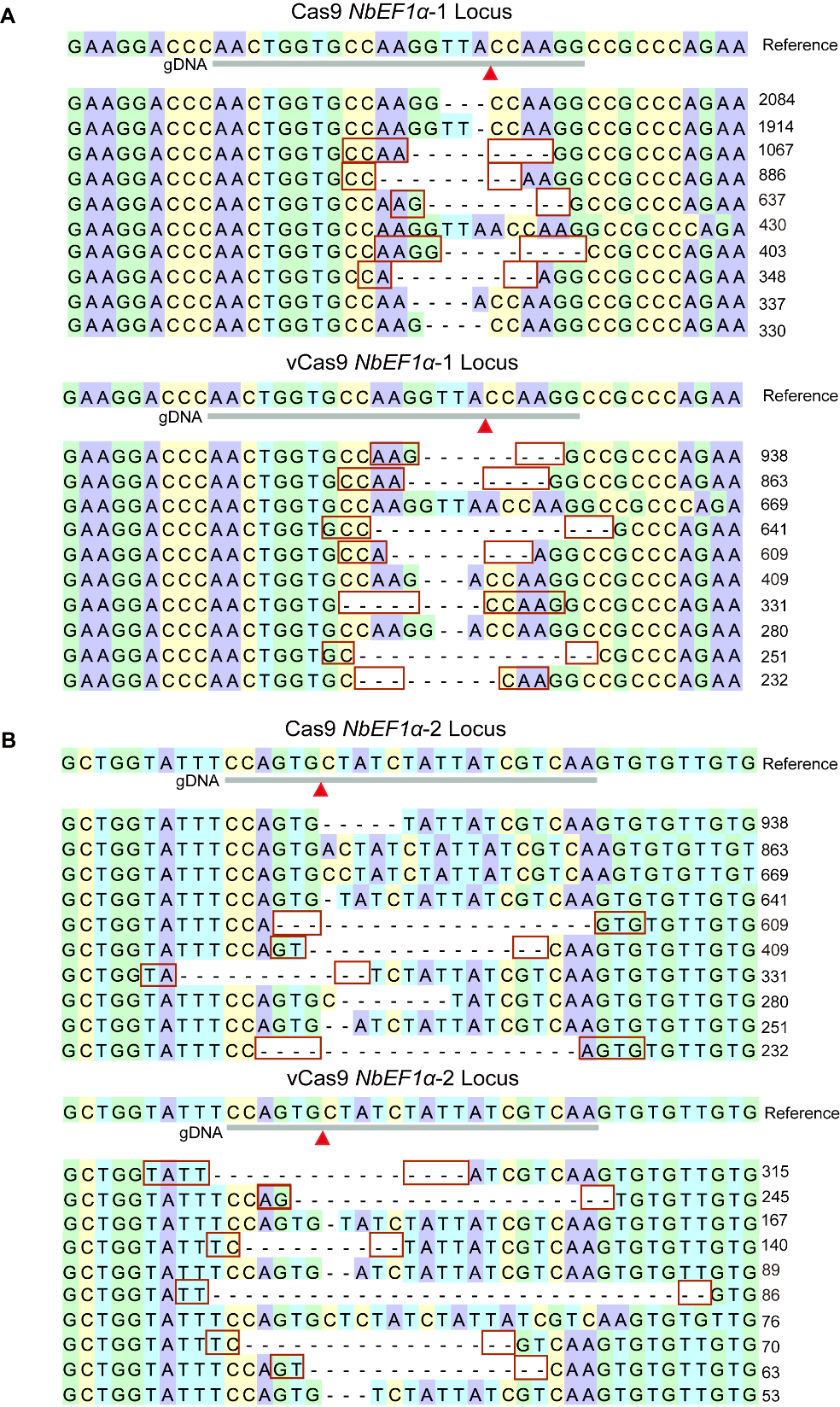


**Figure S6.** **vCas9 suppresses NHEJ and promotes MMEJ at two targets in the *NbEF1α* locus.** The ten most frequent mutation types generated by Cas9 and vCas9 at the (A) *NbEF1α*-1 and (B) *NbEF1α*-2 loci are shown. Deletions at microhomologies are indicated by red boxes. The numbers on the right indicate the read counts for each mutation type.


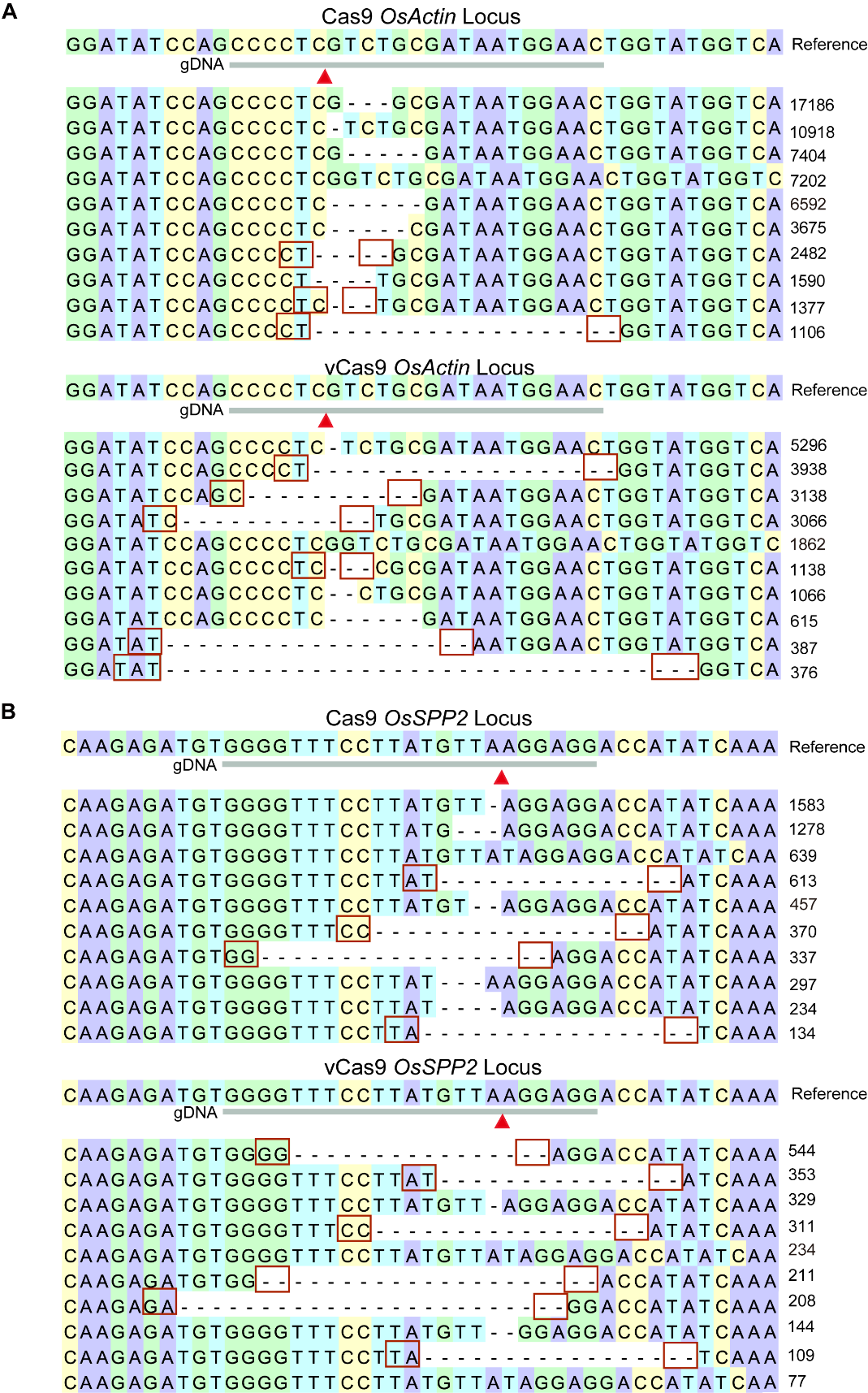


**Figure S7.** **vCas9 suppresses NHEJ and promotes MMEJ in rice.** The ten most frequent mutation types generated by Cas9 and vCas9 at the (A) *OsActin* and (B) *OsSPP2* loci are shown. Deletions at microhomologies are indicated by red boxes. The numbers on the right indicate the read counts for each mutation type.


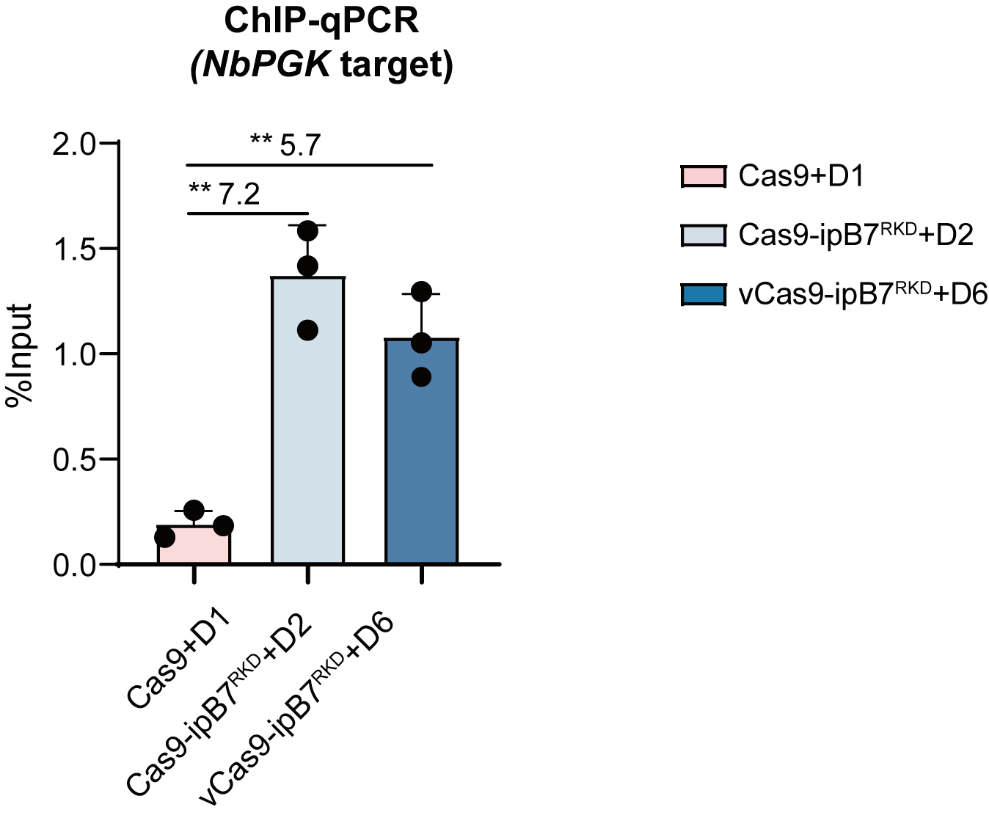


**Figure S8.** **ChIP-qPCR analysis of the interaction between donor DNA and the Cas9 fusion proteins at the *NbPGK* target locus.** The chromatin DNA was immunoprecipitated with Cas9+D1, Cas9-ipB7^RKD^+D2, and vCas9-ipB7^RKD^+D6, followed by qPCR analysis with **TIR-specific primers**. The ChIP signals were calculated as the IP/input ratio (% Input) for each sample. The numbers above the bars indicate the **relative enrichment of the donor DNA,** with the **Cas9+D1** sample as the reference. *p*-values were determined using the two-tailed Student’s t-test: **p* < 0.05, ***p* < 0.01, ****p* < 0.001.

**
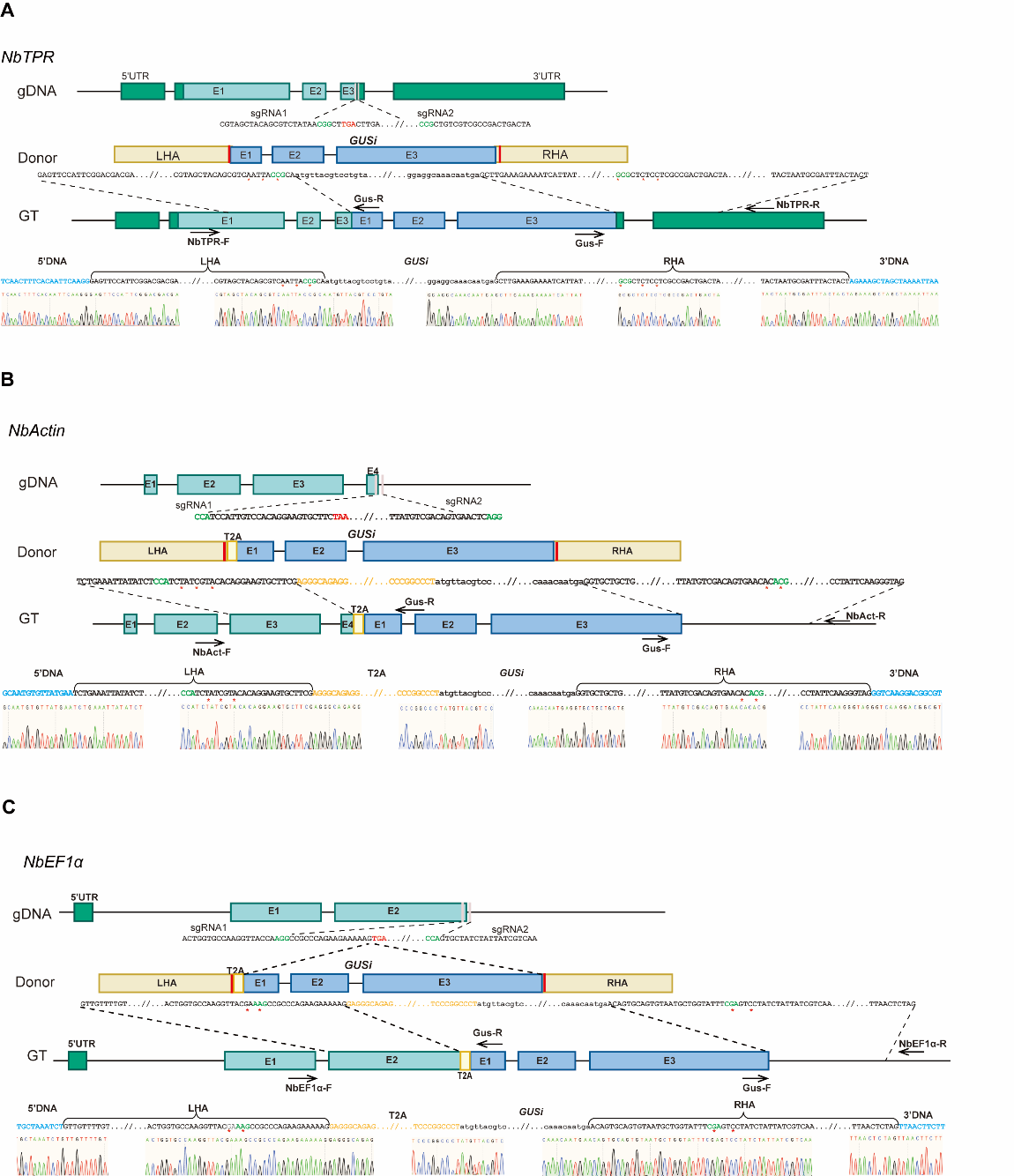
**

**Figure S9.** **Molecular characterization of targeted insertion events at three endogenous loci in *N. benthamiana*.** Targeting strategies and sequence validation for *GUSi* integration at the *NbTPR* locus (A) and *T2A-GUSi* integration at the *NbActin* (B) and *NbEF1α* (C) loci in *N. benthamiana*. UTRs and exons are shown in dark and light green, respectively; left and right homology arms (HAs) in yellow; and the *T2A-GUSi* gene in blue. The target sgRNA is positioned near the stop codon, with PAM sequences highlighted in green and stop codons in red. Sanger sequencing of the junction regions confirmed precise HDR-mediated integration, showing the expected insertions together with synonymous substitutions (*) introduced to prevent re-cleavage.


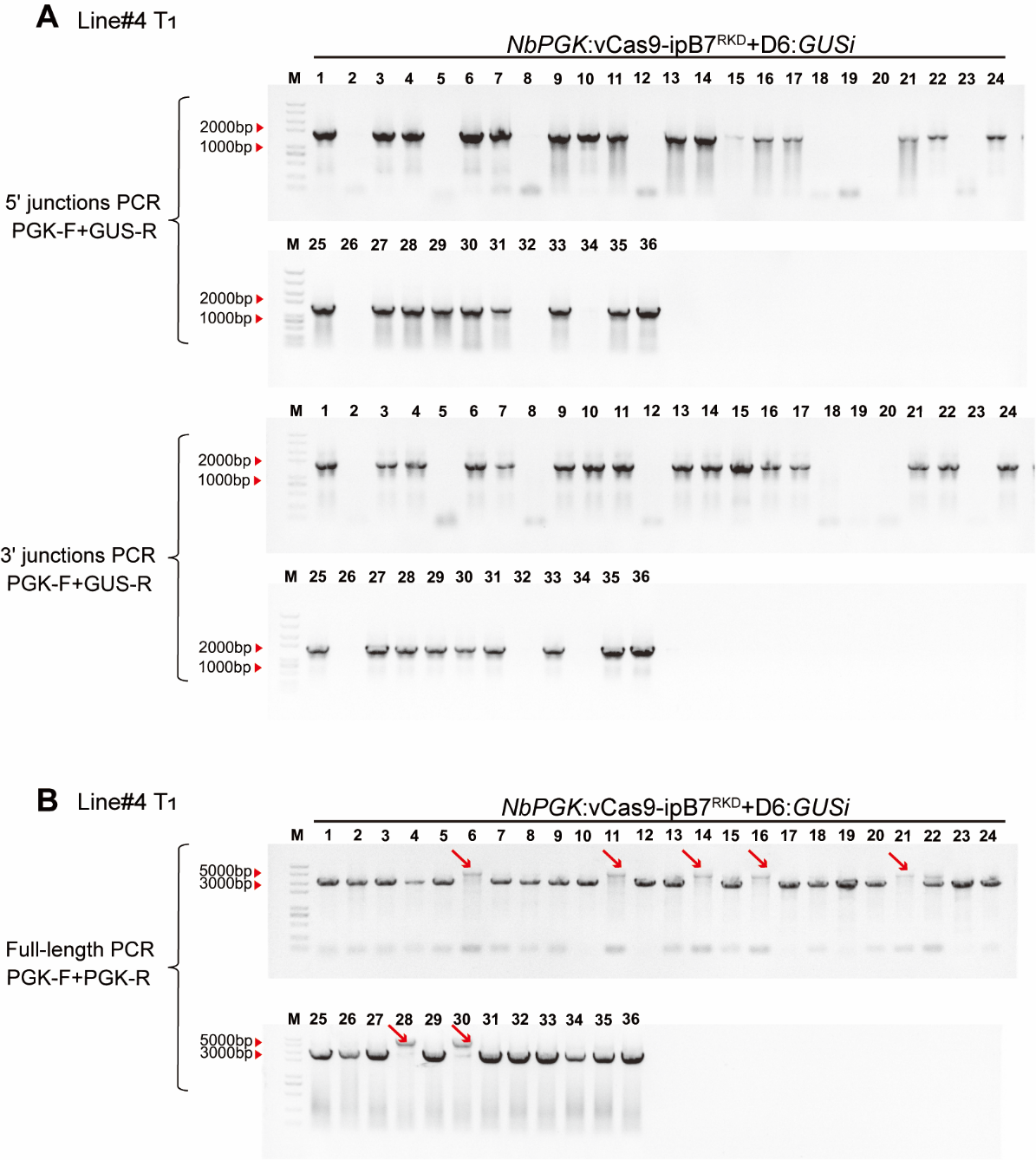


**Figure S10.** **Stable inheritance of targeted *GUSi* insertions at the *NbPGK* locus in line #4.** (A) PCR detection of targeted *GUSi* insertions in 36 T_1_ seedlings from line #4. The 5' (1,758 bp) and 3' (1,718 bp) junction fragments were amplified using primer pairs PGK-F/GUS-R and GUS-F/PGK-R, respectively, and visualized by gel electrophoresis. (B) Full-length knock-in amplification using primers PGK-F/PGK-R (4,984 bp), with expected bands indicated by red arrows.


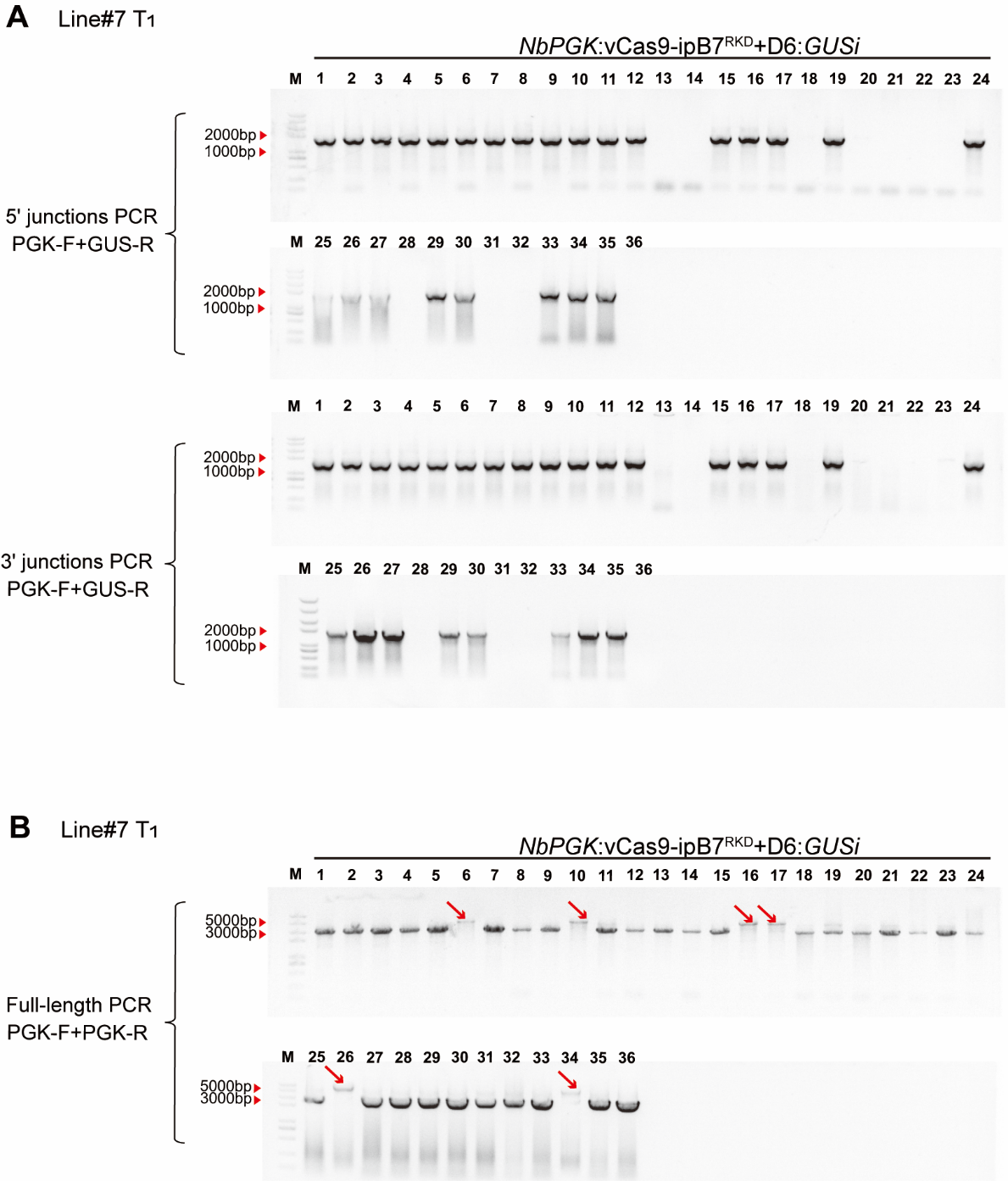


**Figure S11.** **Stable inheritance of targeted *GUSi* insertions at the *NbPGK* locus in line #7.** (A) PCR detection of targeted *GUSi* insertions in 36 T_1_ seedlings from line #7. The 5' (1,758 bp) and 3' (1,718 bp) junction fragments were amplified using primer pairs PGK-F/GUS-R and GUS-F/PGK-R, respectively, and visualized by gel electrophoresis. (B) Full-length knock-in amplification using primers PGK-F/PGK-R (4,984 bp), with expected bands indicated by red arrows.

*
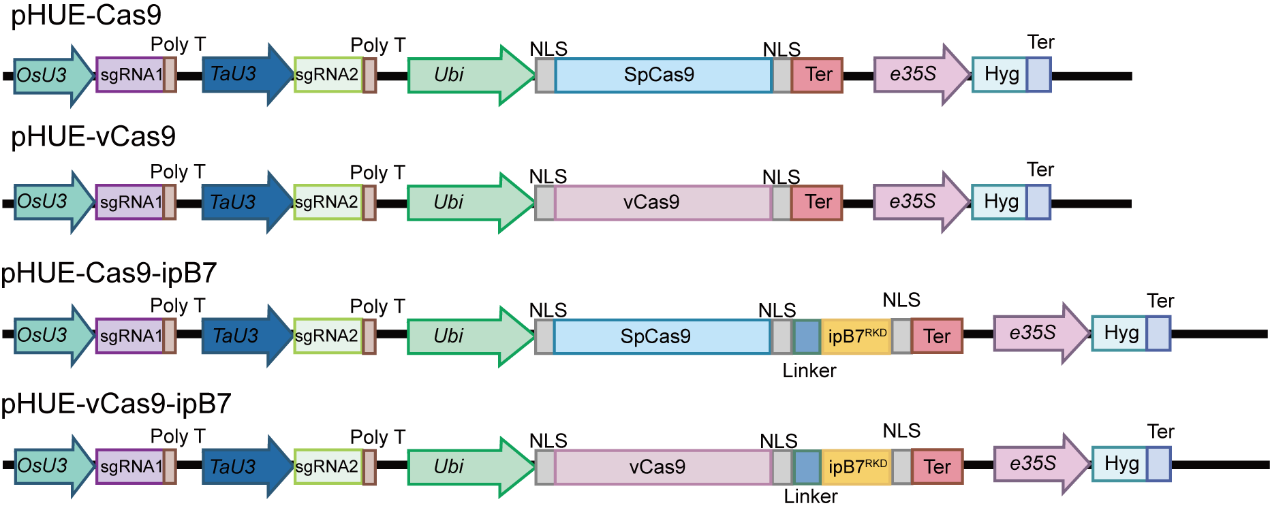
*

**Figure S12.** **Schematic diagrams of binary gene-editing vectors used for monocotyledons.** *OsU3*, snoRNA U3 promoter from *Oryza sativa*; *TaU3*, snoRNA U3 promoter from *Triticum aestivum*; *Ubi*, maize UBIQUITIN 1 promoter; NLS, nuclear localization signal; Hyg, hygromycin.

**
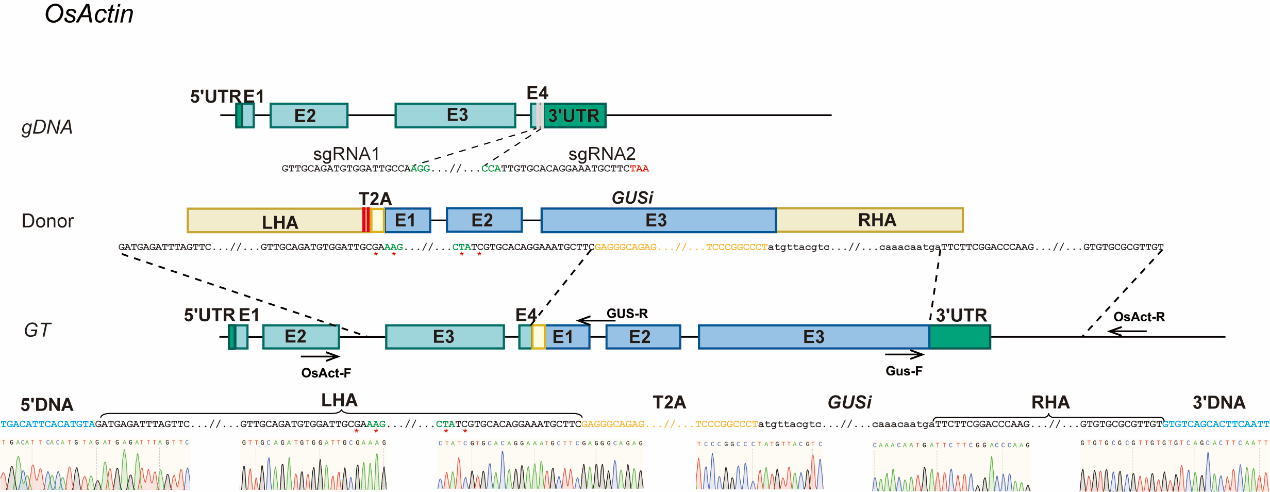
**

**Figure S13.** **Molecular characterization of targeted T2A-GUSi insertion in rice.** Targeting strategies and sequence validation for T2A-GUSi integration downstream of the *OsActin* locus. UTRs and exons are shown in dark and light green, respectively; left and right homology arms (HAs) in yellow; and the *T2A-GUSi* gene in blue. The target sgRNA is positioned near the stop codon, with PAM sequences highlighted in green and stop codons in red. Sanger sequencing of the junction regions confirmed precise HDR-mediated integration, showing the expected *T2A-GUSi* insertions together with synonymous substitutions (*) introduced to prevent re-cleavage.


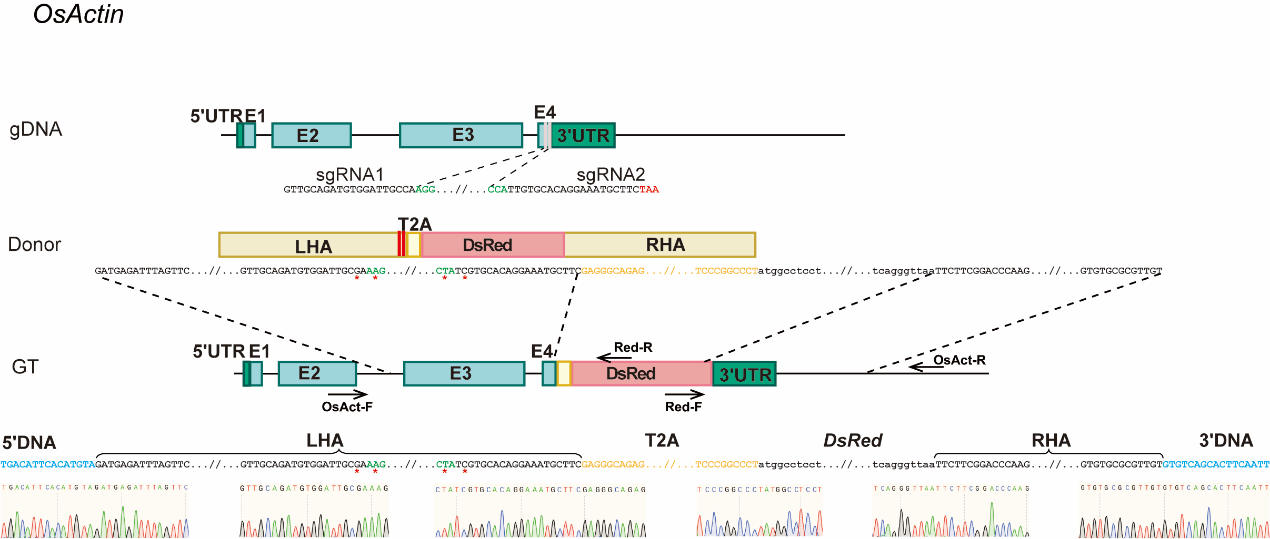


**Figure S14.** **Molecular characterization of targeted T2A-DsRed insertion in rice.** Targeting strategies and sequence validation for T2A-DsRed integration downstream of the *OsActin* locus. UTRs and exons are shown in dark and light green, respectively; left and right homology arms (HAs) in yellow; and the *T2A-DsRed* gene in blue. The target sgRNA is positioned near the stop codon, with PAM sequences highlighted in green and stop codons in red. Sanger sequencing of the junction regions confirmed precise HDR-mediated integration, showing the expected *T2A-DsRed* insertions together with synonymous substitutions (*) introduced to prevent re-cleavage.


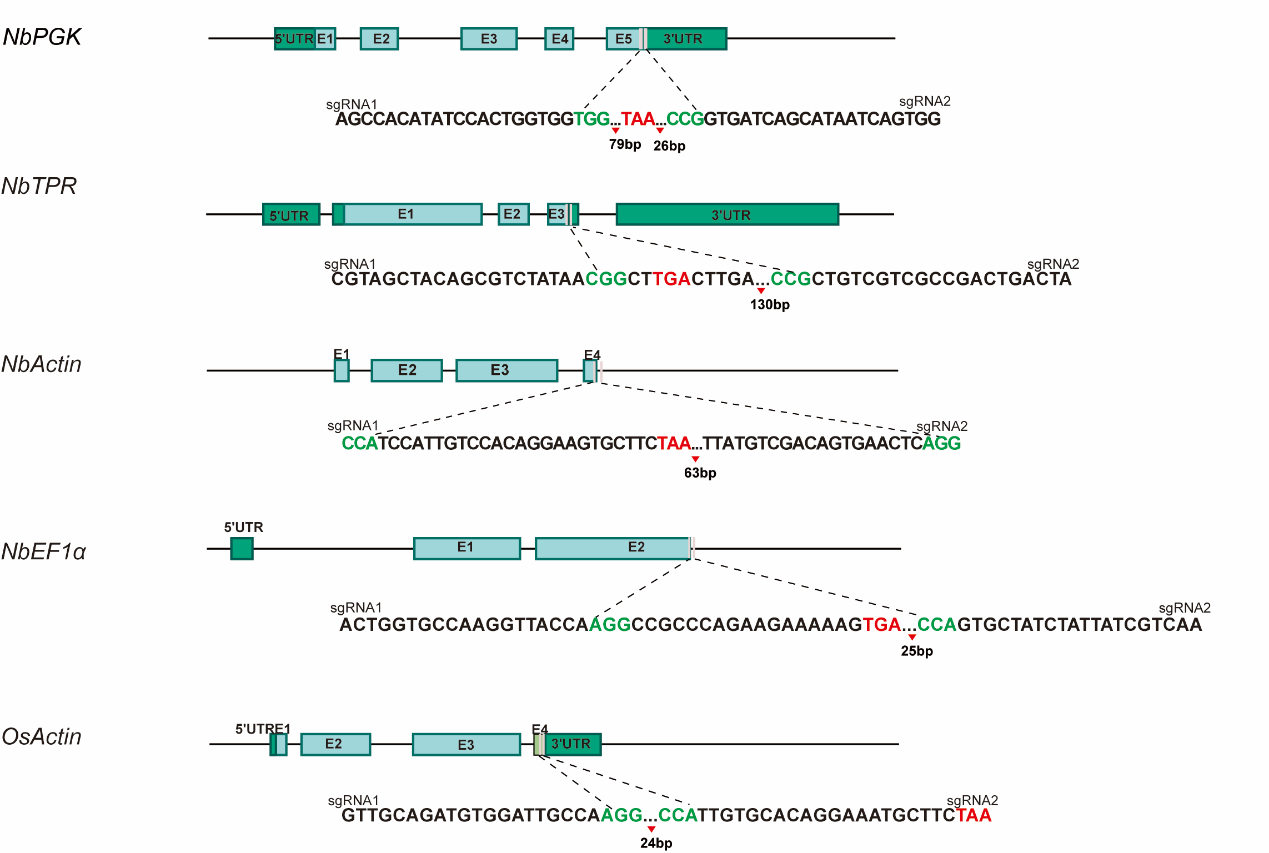


**Figure S15.** **Schematic representation of the flexible distance between the sgRNA target site and the insertion site.** The diagram highlights the variable spacing between the sgRNA target site and the genomic insertion site, demonstrating that this HDR-based strategy is not constrained by the strict PAM proximity required for base editing or prime editing. PAM sequences are shown in green and stop codons in red.


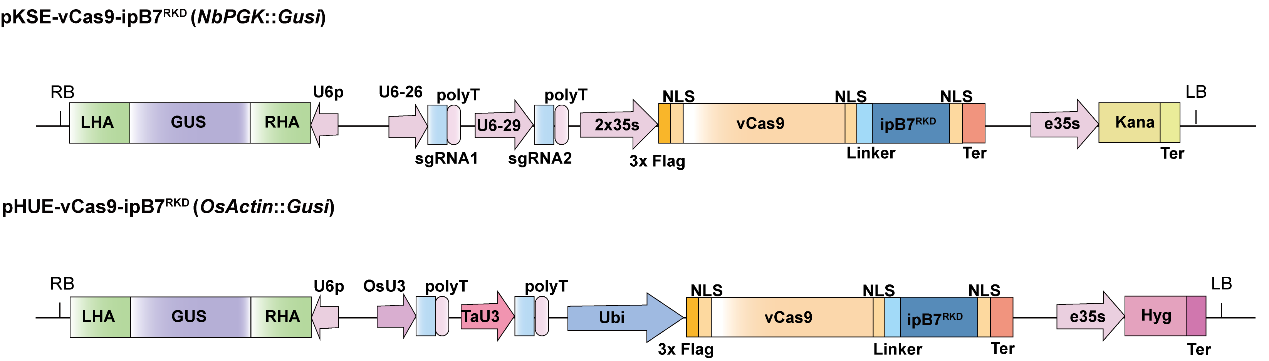


**Figure S16.** **Physical maps of the regions between the right border (RB) and left border (LB).** The sizes of the T-DNA regions and the orientation of core functional elements relative to the T-DNA borders are indicated. LB and RB denote the left and right borders of the T-DNA, respectively.

**Supplementary Tables**

**Table S1. Amplification primers for protein-binding DNA sequences.**

| **Primer Name** | **Primer Sequence (5**'**-3')** |
| --- | --- |
| TIR-F | CAAGCTGCCCTTTTAACCCTAG |
| TIR-R | GCTGCCCTTTTAACATGCGTC |

**Table S2. sgRNA target site.**

| **Target** | **Gene ID** | **Target sequence** | **Application** |
| --- | --- | --- | --- |
| *NbActin*-1 | Niben101Scf06954g67555 | CACTTCCTGTGGACAATGGATGG | Knockout |
| *NbActin*-2 | Niben101Scf06954g67555 | ATTATGTCGACAGTGAACTCAGG |  |
| *NbEF1α*-1 | Niben101Scf08618g01012 | AACTGGTGCCAAGGTTACCAAGG |  |
| *NbEF1α*-2 | Niben101Scf08618g01012 | TTGACGATAATAGATAGCACTGG |  |
| *OsActin* | LOC_Os03g50885 | GTTCCATTATCGCAGACGAGGGG |  |
| *OsSPP2* | LOC_Os01g27880 | GGGGTTTCCTTATGTTAAGGAGG |  |
| *NbPGK*-1 | Niben101Scf05688Ctg073 | AGCCACATATCCACTGGTGGTGG | HDR |
| *NbPGK*-2 | Niben101Scf05688Ctg073 | CCACTGATTATGCTGATCACCGG |  |
| *NbTPR*-1 | Niben101Scf03365Ctg040 | CGTAGCTACAGCGTCTATAACGG |  |
| *NbTPR*-2 | Niben101Scf03365Ctg040 | TAGTCAGTCGGCGACGACAGCGG |  |
| *NbActin*-1 | Niben101Scf06954g67555 | CACTTCCTGTGGACAATGGATGG |  |
| *NbActin*-2 | Niben101Scf06954g67555 | ATTATGTCGACAGTGAACTCAGG |  |
| *NbEF1α*-1 | Niben101Scf08618g01012 | AACTGGTGCCAAGGTTACCAAGG |  |
| *NbEF1α*-2 | Niben101Scf08618g01012 | TTGACGATAATAGATAGCACTGG |  |
| *OsActin-1* | LOC_Os03g50885 | GTTGCAGATGTGGATTGCCAAGG |  |
| *OsActin-2* | LOC_Os03g50885 | GAAGCATTTCCTGTGCACAATGG |  |

**Table S3. Primers used for amplicon deep sequencing.**

| **Primer name** | **Primer sequence (5**'**-3')** | **Application** |
| --- | --- | --- |
| NbActin-F | TATGCTAGTGGTCGTACAACTG | 1st round PCR for deep sequencing |
| NbActin-R | CACACTTCACTTAACAAGGACATG |  |
| NbEF1*α*-F | TGACTGTGCTGTCCTGATTATCGA |  |
| NbEF1*α*-R | CTAGAGTTAAGCTTGTACAATTTAAAGGTG |  |
| 1st-P2-F | TCTAACAAAGTTCATGCCAGAT |  |
| 1st-P2-R | AGAACATTCAAGCCCAGAAG |  |
| OsAct-1st-F | GTCTCGGTCTCGATCTTTG |  |
| OsAct-1st-R | TCCATATCATCCCAGTTGC |  |
| 2nd-NbAct-sg1-F1 | ACAGTGATAAGCTGCATTGTATTGTGG | 2nd round PCR for deep sequencing |
| 2nd-NbAct-sg1-R1 | GCCAATCCTGAGTTCACTGTCGACAT |  |
| 2nd-NbAct-sg1-F2 | CAGATCATAAGCTGCATTGTATTGTGG |  |
| 2nd-NbAct-sg1-R2 | CTTGTACCTGAGTTCACTGTCGACAT |  |
| 2nd-NbAct-sg1-F3 | ATCACGATAAGCTGCATTGTATTGTGG |  |
| 2nd-NbAct-sg1-R3 | TTAGGCCCTGAGTTCACTGTCGACAT |  |
| 2nd-NbAct-sg1-F4 | ACTTGAATAAGCTGCATTGTATTGTGG |  |
| 2nd-NbAct-sg1-R4 | GATCAGCCTGAGTTCACTGTCGACAT |  |
| 2nd-NbAct-sg1-F5 | TAGCTTATAAGCTGCATTGTATTGTGG |  |
| 2nd-NbAct-sg1-R5 | GGCTACCCTGAGTTCACTGTCGACAT |  |
| 2nd-NbAct-sg1-F6 | AGTCAAATAAGCTGCATTGTATTGTGG |  |
| 2nd-NbAct-sg1-R6 | AGTTCCCCTGAGTTCACTGTCGACAT |  |
| 2nd-NbAct-sg2-F1 | ATTCCTTCCATTGTCCACAGGAAGTG |  |
| 2nd-NbAct-sg2-R1 | GTAGAGCCACATTGCATTGTAAACATG |  |
| 2nd-NbAct-sg2-F2 | CAACTATCCATTGTCCACAGGAAGTG |  |
| 2nd-NbAct-sg2-R2 | TACAGCCCACATTGCATTGTAAACATG |  |
| 2nd-NbAct-sg2-F3 | CACGATTCCATTGTCCACAGGAAGTG |  |
| 2nd-NbAct-sg2-R3 | CACTCACCACATTGCATTGTAAACATG |  |
| 2nd-NbAct-sg2-F4 | CAGGCGTCCATTGTCCACAGGAAGTG |  |
| 2nd-NbAct-sg2-R4 | CATGGCCCACATTGCATTGTAAACATG |  |
| 2nd-NbAct-sg2-F5 | CCAACATCCATTGTCCACAGGAAGTG |  |
| 2nd-NbAct-sg2-R5 | ATGTCACCACATTGCATTGTAAACATG |  |
| 2nd-NbAct-sg2-F6 | CTAGCTTCCATTGTCCACAGGAAGTG |  |
| 2nd-NbAct-sg2-R6 | CTATACCCACATTGCATTGTAAACATG |  |
| 2nd-NbEF1*α*-sg1-F1 | ACAGTGTTGCCGAGTACCCACCATTG |  |
| 2nd-NbEF1*α*-sg1-R1 | GCCAATGAGTCTACACAACACACTTGAC |  |
| 2nd-NbEF1*α*-sg1-F2 | CAGATCTTGCCGAGTACCCACCATTG |  |
| 2nd-NbEF1*α*-sg1-R2 | CTTGTAGAGTCTACACAACACACTTGAC |  |
| 2nd-NbEF1*α*-sg1-F3 | ATCACGTTGCCGAGTACCCACCATTG |  |
| 2nd-NbEF1*α*-sg1-R3 | TTAGGCGAGTCTACACAACACACTTGAC |  |
| 2nd-NbEF1*α*-sg1-F4 | ACTTGATTGCCGAGTACCCACCATTG |  |
| 2nd-NbEF1*α*-sg1-R4 | GATCAGGAGTCTACACAACACACTTGAC |  |
| 2nd-NbEF1*α*-sg1-F5 | TAGCTTTTGCCGAGTACCCACCATTG |  |
| 2nd-NbEF1*α*-sg1-R5 | GGCTACGAGTCTACACAACACACTTGAC |  |
| 2nd-NbEF1*α*-sg1-F6 | AGTCAATTGCCGAGTACCCACCATTG |  |
| 2nd-NbEF1*α*-sg1-R6 | AGTTCCGAGTCTACACAACACACTTGAC |  |
| 2nd-NbEF1*α*-sg2-F1 | ATTCCTTGGTGTCATCAAGAGTGTTGA |  |
| 2nd-NbEF1*α*-sg2-R1 | GTAGAGGCTACCTGACATACAAGGCAG |  |
| 2nd-NbEF1*α*-sg2-F2 | CAACTATGGTGTCATCAAGAGTGTTGA |  |
| 2nd-NbEF1*α*-sg2-R2 | TACAGCGCTACCTGACATACAAGGCAG |  |
| 2nd-NbEF1*α*-sg2-F3 | CACGATTGGTGTCATCAAGAGTGTTGA |  |
| 2nd-NbEF1*α*-sg2-R3 | CACTCAGCTACCTGACATACAAGGCAG |  |
| 2nd-NbEF1*α*-sg2-F4 | CAGGCGTGGTGTCATCAAGAGTGTTGA |  |
| 2nd-NbEF1*α*-sg2-R4 | CATGGCGCTACCTGACATACAAGGCAG |  |
| 2nd-NbEF1*α*-sg2-F5 | CCAACATGGTGTCATCAAGAGTGTTGA |  |
| 2nd-NbEF1*α*-sg2-R5 | ATGTCAGCTACCTGACATACAAGGCAG |  |
| 2nd-NbEF1*α*-sg2-F6 | CTAGCTTGGTGTCATCAAGAGTGTTGA |  |
| 2nd-NbEF1*α*-sg2-R6 | CTATACGCTACCTGACATACAAGGCAG |  |
| cas9-2ndOsAct-F | CGATGTGGGTAGAATTTGAATCCCTCAGC |  |
| cas9-2ndOsAct-R | TGACCAATCATCTCCGGCGAACCCAG |  |
| Vcas9-2ndOsAct-F | ACAGTGGGGTAGAATTTGAATCCCTCAGC |  |
| Vcas9-2ndOsAct-R | GCCAATATCATCTCCGGCGAACCCAG |  |
| cas9-RKD-2ndOsAct-F | CAGATCGGGTAGAATTTGAATCCCTCAGC |  |
| cas9-RKD-2ndOsAct-R | CTTGTAATCATCTCCGGCGAACCCAG |  |
| RKD-cas9-2ndOsAct-F | ATCACGGGGTAGAATTTGAATCCCTCAGC |  |
| RKD-cas9-2ndOsAct-R | TTAGGCATCATCTCCGGCGAACCCAG |  |
| Vcas9-RKD-2ndOsAct-F | ACTTGAGGGTAGAATTTGAATCCCTCAGC |  |
| Vcas9-RKD-2ndOsAct-R | GATCAGATCATCTCCGGCGAACCCAG |  |
| RKD-Vcas9-2ndOsAct-F | TAGCTTGGGTAGAATTTGAATCCCTCAGC |  |
| RKD-Vcas9-2ndOsAct-R | GGCTACATCATCTCCGGCGAACCCAG |  |
| 2nd-P2- F1 | GTGAAACTGGTATTATCGAAGCTATTG |  |
| 2nd-P2-R1 | CAAAAGAGTACTGTACAACTGAATCTG |  |
| 2nd-P2-F2 | CGTACGCTGGTATTATCGAAGCTATTG |  |
| 2nd-P2-R2 | CACGATAGTACTGTACAACTGAATCTG |  |
| 2nd-P2-F3 | CGATGTCTGGTATTATCGAAGCTATTG |  |
| 2nd-P2-R3 | TGACCAAGTACTGTACAACTGAATCTG |  |
| 2nd-P2-F4 | ACAGTGCTGGTATTATCGAAGCTATTG |  |
| 2nd-P2-R4 | GCCAATAGTACTGTACAACTGAATCTG |  |
| 2nd-P2-F5 | CAGATCCTGGTATTATCGAAGCTATTG |  |
| 2nd-P2-R5 | CTTGTAAGTACTGTACAACTGAATCTG |  |
| 2nd-P2-F6 | ATCACGCTGGTATTATCGAAGCTATTG |  |
| 2nd-P2-R6 | TTAGGCAGTACTGTACAACTGAATCTG |  |

**Table S4.** **Primer sequences used for ChIP-qPCR.**

| **Primer Name** | **Primer Sequence (5'-3')** |
| --- | --- |
| qTIR-PGK-F | CAAGATTGGAGTGATCGAATC |
| qTIR-PGK-R | GAGTGATGTAGCGAGTTCTAG |

**Table S5. Primers for homologous recombination detection.**

| **Primer Name** | **Primer Sequence (5'-3')** | **Application** |
| --- | --- | --- |
| PGK-F | GTATGCTCTACCAATAGGTTGTG | Detection of *NbPGK*::*GUSi* |
| GUS-R | TTCCCACCAACGCTGATCAATTCCAC |  |
| GUS-F | TGAACAGGTATGGAATTTCGCC |  |
| PGK-R | GCCATCTTGCTATTGAACTCGTTGC |  |
| NbTPR-F | GCAATGGCAGACAATTAGCATCTCATGGTATTAG | Detection of *NbTPR*::*GUSi* |
| GUS-R | TTCCCACCAACGCTGATCAATTCCAC |  |
| GUS-F | TGAACAGGTATGGAATTTCGCC |  |
| NbTPR-R | GAATACCTCAGGGTTTCCAAAATGC |  |
| NbActin-F | ATGACATGGAGAAGATATGGCATCATACC | Detection of *NbActin*::*GUSi* |
| GUS-R | TTCCCACCAACGCTGATCAATTCCAC |  |
| GUS-F | TGAACAGGTATGGAATTTCGCC |  |
| NbActin-R | GGTTTCAGGATATGGCCATTCTGGAGTTAG |  |
| NbEF1α-F | GCTGACTGTGCTGTCCTGATTATCGA | Detection of *NbEF1α*::*GUSi* |
| GUS-R | TTCCCACCAACGCTGATCAATTCCAC |  |
| GUS-F | TGAACAGGTATGGAATTTCGCC |  |
| NbEF1α-R | CTACTAGTTCGCTTTGCAAACACAGG |  |
| OsAct-F | CGTACCACAGGTGAGCACATTCGAC | Detection of *OsActin*::*GUSi* |
| GUS-R | TTCCCACCAACGCTGATCAATTCCAC |  |
| GUS-F | TGAACAGGTATGGAATTTCGCC |  |
| OsAct-R | GTGATGACATCTGCATAACTTGAAAGATG |  |
| OsAct-F | CGTACCACAGGTGAGCACATTCGAC | Detection of *OsActin*::*DsRed* |
| DsRed-R | CTCGATCTCGAACTCGTGGC |  |
| DsRed-F | CTACTACTACGTGGACGCCAAGC |  |
| OsAct-R | GTGATGACATCTGCATAACTTGAAAGATG |  |

**Table S6. Summary of whole-genome sequencing data.**

| **Lines** | **Genotype of KI** | **Clean reads** | **Mapping rate** | **Depth** | **Genome coverage (4X)** |
| --- | --- | --- | --- | --- | --- |
| OsAct-GUSi1 | *OsActin*::*GUSi* | 121,875,414 | 99.60% | 39.86 | 99.58% |
| OsAct-GUSi2 | *OsActin*::*GUSi* | 121,997,490 | 99.69% | 39.66 | 99.52% |
| OsAct-GUSi3 | *OsActin*::*GUSi* | 121,402,204 | 99.66% | 38.69 | 99.48% |
| OsAct-DsRed1 | *OsActin*::*DsRed* | 124,022,237 | 99.65% | 39.92 | 99.57% |
| OsAct-DsRed2 | *OsActin*::*DsRed* | 123,403,298 | 99.67% | 39.65 | 99.51% |
| OsAct-DsRed3 | *OsActin*::*DsRed* | 123,605,255 | 99.60% | 40.96 | 99.52% |

**Table S7. Detection of knock-in events by WGS.**

| **Sample** | **#Chr1** | **Pos1** | **Orientation1** | **Type** | **Score** | **num_Reads** |
| --- | --- | --- | --- | --- | --- | --- |
| GUSi1 | 3 | 29075698 | 2+106- | CTX | 99 | 104 |
| GUSi1 | 3 | 29075464 | 81+6- | CTX | 99 | 79 |
| GUSi2 | 3 | 29075759 | 0+62- | CTX | 99 | 58 |
| GUSi2 | 3 | 29075456 | 32+0- | CTX | 99 | 32 |
| GUSi3 | 3 | 29075465 | 159+0- | CTX | 99 | 158 |
| GUSi3 | 3 | 29075749 | 2+152- | CTX | 99 | 152 |
| Red1 | 3 | 29075464 | 52+0- | CTX | 99 | 52 |
| Red1 | 3 | 29075690 | 2+61- | CTX | 99 | 6 |
| Red1 | 3 | 29075690 | 2+61- | CTX | 99 | 55 |
| Red2 | 3 | 29075451 | 80+1- | CTX | 99 | 71 |
| Red2 | 3 | 29075824 | 2+63- | CTX | 99 | 51 |
| Red3 | 3 | 29075464 | 71+0- | CTX | 99 | 55 |
| Red3 | 3 | 29075703 | 1+69- | CTX | 99 | 63 |

**Chr1:** Chromosome identifier where the structural variation is located (ID1).

**Pos1:** Genomic start position of the variant on the chromosome (Position 1).

**Orientation1:** Strand orientation (forward/reverse) of reads mapping to this region (Information 1).

**Type:** Type of structural variation (DEL: deletion; INS: insertion; INV: inversion; ITX: intra‑chromosomal translocation; CTX: inter‑chromosomal translocation).

**Score:** Confidence score for the SV call (filter threshold = 30).

**num_Reads:** Number of paired‑end reads supporting the structural variation.

**Table S8. The off-target sites of *OsActin***

| **Target** | **On-or Off-target** | **Spacer with PAM sequence** |
| --- | --- | --- |
| sgRNA-1 | *OsActin*-on | TTGCAGATGTGGATTGCCAAGG |
|  | *OsActin*-off1 | aTGCAGATGTGGATTGCCAAGG |
|  | *OsActin*-off2 | TTGtgGATGTGGATTGCCATGG |
|  | *OsActin*-off3 | TTtCAGATGTGGAcTGCtAGGG |
|  | *OsActin*-off4 | TTGgAGATGTcGATTGCaATGG |
|  | *OsActin*-off5 | TTGCAaATGTtGATaGCCAGGG |
|  | *OsActin*-off6 | TTGgAGgTGTGGcTTGCCATGG |
|  | *OsActin*-off7 | TTGCAGATGTGGgTTaCCtTGG |
|  | *OsActin*-off8 | TTGCAGATGTGGATatCaAAGG |
|  | *OsActin*-off9 | TTGCAGgTtaGGATTGCCATGG |
|  | *OsActin*-off10 | TTcCAGAaGcGGATTGCCAAGG |
|  | *OsActin*-off11 | TTGCAGtaGTGGATTGaCAAGG |
|  | *OsActin*-off12 | TTGCAGATaTGGAagGCCAGGG |
|  | *OsActin*-off13 | TTGCAGgTtTGGgTgGCCAGGG |
|  | *OsActin*-off14 | gTGCtGATGTGGcaTGCCATGG |
|  | *OsActin*-off15 | TTGCAGgTtTGGgTgGCCAAGG |
|  | *OsActin*-off16 | TTGtAGATGTGtAcTGCtATGG |
|  | *OsActin*-off17 | TTGCAGATccGGgTTGtCATGG |
|  | *OsActin*-off18 | TTtCAGgTGaGGATTGCtATGG |
|  | *OsActin*-off19 | TTGCcGATGTGGAaTGgCgAGG |
|  | *OsActin*-off20 | TTGCcGAcGTGGATTGCtcAGG |
|  | *OsActin*-off21 | TcaCAGATGTGGATctCCAAGG |
|  | *OsActin*-off22 | TaGCAGcTGTaGATTGCCtCGG |
|  | *OsActin*-off23 | TTttAGATGTGcATaGCCATGG |
|  | *OsActin*-off24 | TTGCAGgTtTGGgTgGCCAAGG |
|  | *OsActin*-off25 | TTGCAGATGatGAgTGCgAGGG |
|  | *OsActin*-off26 | gTGCAGcTtTGGATTtCCACGG |
|  | *OsActin*-off27 | TTGCActTtTGGATTGCgATGG |
|  | *OsActin*-off28 | TgGCtGATGaGGATcGCCAGGG |
|  | *OsActin*-off29 | gTGCAGgTGgtGATTGCCAAGG |
|  | *OsActin*-off30 | TTttAGAgGTcGATTGCCATGG |
|  | *OsActin*-off31 | gTGCtGATGTGGcaTGCCATGG |
|  | *OsActin*-off32 | TTGCtGATcTGGgTcGCCATGG |
|  | *OsActin*-off33 | TTGgtGATcTGaATTGCCAAGG |
|  | *OsActin*-off34 | aTGCtGATGTGGcaTGCCACGG |
|  | *OsActin*-off35 | TcGCAGATGTGcAgTGCCcAGG |
|  | *OsActin*-off36 | TTGCAGgTtTGGgTgGCCAAGG |
|  | *OsActin*-off37 | TcGCAGATGTGGATatCtAAGG |
|  | *OsActin*-off38 | TTGCAGAaGTtGATcGgCAAGG |
|  | *OsActin*-off39 | TTGCAGgTtTGGgTgGCCAAGG |
|  | *OsActin*-off40 | TTGCAatTGTGGATTtCCcGGG |
|  | *OsActin*-off41 | TccCAGAaGTGGAcTGCCAAGG |
|  | *OsActin*-off42 | TcGCAGATGTcGATgGtCATGG |
|  | *OsActin*-off43 | TcGCAGATGTcGATcGtCATGG |
|  | *OsActin*-off44 | TTGCAcATGcGcATTtCCATGG |
|  | *OsActin*-off45 | TTGaAGgTaaGGATTGCCATGG |
|  | *OsActin*-off46 | TaGCAGAgGTGGAgTGCaAGGG |
|  | *OsActin*-off47 | TTGCAGATaaGGAgTGgCAAGG |
|  | *OsActin*-off48 | TTGCtGcTGgaGATTGCCAAGG |
|  | *OsActin*-off49 | TTGCAGAatTaGATTtCCACGG |
|  | *OsActin*-off50 | TTGCAtATagtGATTGCCACGG |
|  | *OsActin*-off51 | TTcCAGAaGcGGAcTGCCAAGG |
|  | *OsActin*-off52 | TTGCAtcTGTGGAcTtCCATGG |
|  | *OsActin*-off53 | TaGCAGAaGTGGATaGCgATGG |
|  | *OsActin*-off54 | TcaCAGATGTGGATctCCAAGG |
|  | *OsActin*-off55 | gTGCgGATGTGGcaTGCCATGG |
|  | *OsActin*-off56 | TTtCAGgTcaGGATTGCCATGG |
|  | *OsActin*-off57 | TTGCAGAaGctGATTGCgATGG |
|  | *OsActin*-off58 | TTGCtGATtTtGATTGCtATGG |
|  | *OsActin*-off59 | TTGggGtTGTaGATTGCCATGG |
|  | *OsActin*-off60 | TTagAGATGTGaAcTGCCATGG |
|  | *OsActin*-off61 | TgGCAtATtTGcATTGCCATGG |
|  | *OsActin*-off62 | cTGCAGATGTGtAggGCCAGGG |
|  | *OsActin*-off63 | TTGaAGcTGTGcATTGgCAGGG |
|  | *OsActin*-off64 | gTGCtGATGTGGcaTGCCATGG |
|  | *OsActin*-off65 | TTGCAGgTGaGGcTcGCCATGG |
|  | *OsActin*-off66 | gTGCAGgTGaGGcTTGCCATGG |
|  | *OsActin*-off67 | TTGagGAgGTGGATgGCCATGG |
|  | *OsActin*-off68 | TgGCgGAgGTGGATTGCCtTGG |
|  | *OsActin*-off69 | TgGCgGAgGTGGATTGCCtTGG |
|  | *OsActin*-off70 | TTtCAGcgGTtGATTGCCAAGG |
|  | *OsActin*-off71 | TTGCAGAaGTtGATcGgCAAGG |
|  | *OsActin*-off72 | cTGCAGATtgGGATaGCCATGG |
|  | *OsActin*-off73 | TTGCAGAaGTtGATcGgCAAGG |
|  | *OsActin*-off74 | agGaAGATGTGGATcGCCAAGG |
|  | *OsActin*-off75 | TTcaAGATGgGGtTTGCCATGG |
|  | *OsActin*-off76 | aTGCAGgTGcaGATTGCCATGG |
|  | *OsActin*-off77 | TTGggGATGTGGAgTGaCACGG |
|  | *OsActin*-off78 | TgGCgGAgGTGGATTGCCtTGG |
|  | *OsActin*-off79 | gTGCAGATGTGGATctCaAGGG |
|  | *OsActin*-off80 | TTGCcGtTGTGGATgaCCACGG |
|  | *OsActin*-off81 | TTGCAagTGTGGATTttCAAGG |
|  | *OsActin*-off82 | TTGCAaATGTGGAgTaaCAGGG |
|  | *OsActin*-off83 | TaGCAGATaTGGATTcCtAAGG |
|  | *OsActin*-off84 | TTGCAGATGTGGATgGtggGGG |
|  | *OsActin*-off85 | TTGtcGATGTGGATTcCgAGGG |
|  | *OsActin*-off86 | TcGCAGATGgGaATTGCaATGG |
|  | *OsActin*-off87 | aTcCAGATGTGGgTTcCCAAGG |
|  | *OsActin*-off88 | TgGCgGAgGTGGATTGCCtTGG |
|  | *OsActin*-off89 | TgGtgGATGTGGATTGtCATGG |
|  | *OsActin*-off90 | TgGCgGAgGTGGATTGCCtTGG |
|  | *OsActin*-off91 | TTGCAGcTGTGcAaTGtCATGG |
|  | *OsActin*-off92 | TTGCAGATGTGtATcGCttCGG |
| sgRNA-2 | *OsActin*-on | AAGCATTTCCTGTGCACAATGG |
|  | *OsActin*-off1 | cAGCATaTCCTGTGCACAcGGG |
|  | *OsActin*-off2 | cAGCATTTCCTGTGCtgAATGG |
|  | *OsActin*-off3 | AAGCATTTCCTGatgACAAGGG |
|  | *OsActin*-off4 | AAGCtTTTtCTGTcCACAAAGG |
|  | *OsActin*-off5 | AAGCATTTtCTGTGCctAgTGG |
|  | *OsActin*-off6 | gAGCATcTCCgGTGCtCAAGGG |
|  | *OsActin*-off7 | AAGCgTaTCCTaTGCACAcAGG |
|  | *OsActin*-off8 | gAGCATaTCCTaTGCACAcAGG |
|  | *OsActin*-off9 | AAatATTTtCaGTGCACAAAGG |
|  | *OsActin*-off10 | AtGttTTTCCTGTGCtCAATGG |
|  | *OsActin*-off11 | AtGttTTTCCTGTGCtCAATGG |
|  | *OsActin*-off12 | AAGggTaTCCTGTGCACAcGGG |
|  | *OsActin*-off13 | gAGCATTTCCTGTGgtCtAGGG |
|  | *OsActin*-off14 | AAGCtTTTCCTGaaCACAcGGG |
|  | *OsActin*-off15 | AAGCATTTCCTtTtCtCAgTGG |
|  | *OsActin*-off16 | AAGCATTTgtTGTGaAaAAGGG |
|  | *OsActin*-off17 | AAGCAcTTCtTGTGaACgATGG |
|  | *OsActin*-off18 | gAaCATaTCCTGTGCACAcAGG |
|  | *OsActin*-off19 | AAGCcTcTgtTGTGCACAATGG |
|  | *OsActin*-off20 | AAGCAcTaCtTGcGCACAAGGG |
|  | *OsActin*-off21 | AgGCATTTCCTaatCACAAGGG |
|  | *OsActin*-off22 | AgGCATTTCCTaatCACAAGGG |
|  | *OsActin*-off23 | AAGCAgTTCaaGTGCcCAAGGG |
|  | *OsActin*-off24 | tccCATTTCCTGTGCACAgGGG |
|  | *OsActin*-off25 | AAGgATTatCTGTGCAtAAAGG |
|  | *OsActin*-off26 | AAGgATTatCTGTGCAtAAAGG |
|  | *OsActin*-off27 | AAaCATggCCTaTGCACAACGG |
|  | *OsActin*-off28 | gAGCATaTCCTaTGCACAcAGG |
|  | *OsActin*-off29 | cAGCATTTCtTaTGaACAATGG |
|  | *OsActin*-off30 | AAGCATTTCCTtTGaAtAgAGG |
|  | *OsActin*-off31 | cAtCATTTCCTGTaCtCAATGG |
|  | *OsActin*-off32 | AAGCATaTgCTaTaCACAATGG |
|  | *OsActin*-off33 | AAGCgTccCCTGTGCAaAATGG |
|  | *OsActin*-off34 | gAGCATaTCCTaTGCACAcAGG |
|  | *OsActin*-off35 | AAGCATcTtCTtTcCACAAAGG |
|  | *OsActin*-off36 | gAGtcTTTCCTGTcCACAAGGG |
|  | *OsActin*-off37 | gAGCATaTgCcGTGCACAATGG |
|  | *OsActin*-off38 | AAtaATTTCtTGTGCAtAAAGG |
|  | *OsActin*-off39 | gAGCATcTCCgGTGCtCAAGGG |
|  | *OsActin*-off40 | AAtCAaTTCaTGTGCACAcTGG |
|  | *OsActin*-off41 | AAaCgTTTCCTGTGCAttATGG |
|  | *OsActin*-off42 | gAGtATTaCtTGTGCACAAGGG |
|  | *OsActin*-off43 | AAGCcTTTCaTGcGCACAtAGG |
|  | *OsActin*-off44 | AAtaATTTCtTGTGCAtAAAGG |
|  | *OsActin*-off45 | AAGgATagCCTGTGCAgAAGGG |
|  | *OsActin*-off46 | gAGtcTTTCCTGTcCACAAGGG |

**Table S9. Detection of off-target effects by WGS.**

| **Target** | **Mismatch** | **Target site number** | **GUSi1**  **(VAF)** | **GUSi2**  **(VAF)** | **GUSi3**  **(VAF)** | **Red1**  **(VAF)** | **Red2**  **(VAF)** | **Red3**  **(VAF)** |
| --- | --- | --- | --- | --- | --- | --- | --- | --- |
| sgRNA1 | 0 | 1 | 0.53 | 0.46 | 0.62 | 0.48 | 0.37 | 0.7 |
|  | 1 | 1 | 0.47 | 0.17 | 0 | 0.15 | 0.91 | 0.28 |
|  | 2 | 1 | 0.42 | 0 | 0 | 0 | 0.29 | 0.48 |
|  | 3 | 10 | 0 | 0 | 0 | 0 | 0 | 0 |
|  | 4 | 80 | 0 | 0 | 0 | 0 | 0 | 0 |
|  | 5 | 53 | 0 | 0 | 0 | 0 | 0 | 0 |
| sgRNA2 | 0 | 1 | 0.52 | 0.46 | 0.62 | 0.48 | 0.27 | 0.28 |
|  | 1 | 0 | 0 | 0 | 0 | 0 | 0 | 0 |
|  | 2 | 0 | 0 | 0 | 0 | 0 | 0 | 0 |
|  | 3 | 4 | 0 | 0 | 0 | 0 | 0 | 0 |
|  | 4 | 42 | 0 | 0 | 0 | 0 | 0 | 0 |
|  | 5 | 54 | 0 | 0 | 0 | 0 | 0 | 0 |

**Variant Allele Frequency (VAF):** proportion of sequencing reads supporting a specific allele at a given genomic locus, relative to the total sequencing depth at that locus.

**Table S10. Primers used for vector construction in this study.**

| **Primer Name** | **Primer Sequence (5'-3')** | **Application** |
| --- | --- | --- |
| **a. Construction of prokaryotic expression vectors** | | |
| His-F | CTCGAGCACCACCACCACCACCACTGAG | Construction of pET28a(+)-ipB7^RKD^ |
| RBS-R | CATGGTATATCTCCTTCTTAAAGTTAAAC |  |
| RBS-pB-F | AAGAAGGAGATATACCATGGGATCCAGTCTCGACGAC |  |
| His-pB-R | GGTGGTGGTGGTGCTCGAGGGCCGCCGGCACGCCATGAAT |  |
| His-F | CTCGAGCACCACCACCACCACCACTGAG | Construction of pET28a(+)-Cas9-ipB7^RKD^ |
| RBS-R | CATGGTATATCTCCTTCTTAAAGTTAAAC |  |
| Cas9-F | AAGAAGGAGATATACCATGCCC |  |
| Cas9-R | CACCTTCCTCTTCTTCTTGGGGTCACCTCCTAGCTGACTC |  |
| Cas9-pB-F | CCAAGAAGAAGAGGAAGGTGAGCGGAGGATCTTCCGGAGG |  |
| His-pB-R | GGTGGTGGTGGTGCTCGAGGGCCGCCGGCACGCCATGAAT |  |
| His-F | CTCGAGCACCACCACCACCACCACTGAG | Construction of pET28a(+)-ipB7^RKD^-Cas9 |
| RBS-R | CATGGTATATCTCCTTCTTAAAGTTAAAC |  |
| RBS-pB-F | AAGAAGGAGATATACCATGGGATCCAGTCTCGACGAC |  |
| Linker-pB-R | CCTCCGGAAGATCCTCCGCTGGCCGCCGGCACGCCATGAA |  |
| linker-F | AGCGGAGGATCTTCCGGAG |  |
| linker-R | CATAGATCCTCCGGAAGAGCCTC |  |
| Linker-Cas9-F | GCTCTTCCGGAGGATCTATGCCCAAGAAGAAGAGGAAGGTGATGGATAAGAAATACTCA |  |
| His-Cas9-R | TGGTGGTGGTGGTGCTCGAGCACCTTCCTCTTCTTCTTGGGGTCACCTCCTAGCTGACT |  |
| **b. Construction of tobacco/rice knockout vectors** | | |
| EcoRI-Cas9-F | CCGGAATTCCCTAGGCCTACTAGATGGAT | Construction of pEasy-Cas9 |
| SacI-Cas9-R | TCTGAGCTCACTTCTTCTTCTTCG |  |
| S55R-F | GCGCTCTCCTGTTCGACAGAGGCGAGACGGC | Construction of pEasy-vCas9 |
| S55R-R | TCTGTCGAACAGGAGAGCGCCAATGAGATTC |  |
| R976A-F | ATTTCCAGTTCTACAAGGTTGCGGAGATCAAC |  |
| R976A-R | GCAACCTTGTAGAACTGGAAATCCTTCCTGAA |  |
| K1003A-F | CTCTGATCAAGAAGTACCCAGCGCTCGAGAGC |  |
| K1003A-R | GCTGGGTACTTCTTGATCAGAGCTGTGCCGAC |  |
| T1314R-F | AGAATATCATTCATCTCTTCCGCCTGACGAAC |  |
| T1314R-R | CGGAAGAGATGAATGATATTCTCAGCCTGCTC |  |
| MluI-F | ATGACGCGTACCTGAACGCGGTGG | Construction of pHUE-Cas9/vCas9-ipB7^RKD^ |
| AKS-R | TCTGAGCTCGGTACCATTCTTAAGCTTCTTCTTCTTCGCCTGCC |  |
| AflII-ipB7-F | AAGCTTAAGAGCGGAGGATCTTCCGGAGG |  |
| SacI-ipB7-R | TCTGAGCTCTCAGGCCGCCGGCACGCCAT |  |
| NLS-F | GCGCCCAAAAAAAAACGAAAAGTGGGCATTCATGGCGTGCCGGCGGCC | Construction of pEasy-NLS-ipB7^RKD^-Linker |
| NLS-ipB7-F | CATGGCGTGCCGGCGGCCATGGGATCCAGTCTCGACGA |  |
| 1R | ATCCTCCGCTGAAGCAAGACTGGCACATG |  |
| 1F | GCATCTAGATGCGCCCAAAAAAAAACGAAAAGTG |  |
| 1R | ATCCTCCGCTGAAGCAAGACTGGCACATG |  |
| 2F | GTCTTGCTTCAGCGGAGGATCTTCCGGAGG |  |
| 2R | GCTCTTAAACAGATCCTCCGGAAGAGCCTC |  |
| 3F | CGGAGGATCTGTTTAAGAGCTATGCTGGAA |  |
| 3R | TTTTGGGCGCATCTAGATGCATTCGCGAGG |  |
| pBCas9-F1 | AGCCCTAGGATGGCGCCCAAAAAAAAACGAAAAGTGGGC | Construction of pHUE-ipB7^RKD^-Cas9/vCas9 |
| pBCas9-R1 | TCCTCTTCTTCTTCGGAGCAGATCCTCCGGAAGAGCCT |  |
| pBCas9–F2 | AGGCTCTTCCGGAGGATCTGCTCCGAAGAAGAAGAGGA |  |
| pBCas9–R2 | TCTCCTGCAGGTAGCAGATGCGATTC |  |
| T-NbAct*-*sg1-F | ATTGCACTTCCTGTGGACAATGGA | Target-specific primers |
| T-NbAct*-*sg1-R | AAACTCCATTGTCCACAGGAAGTG |  |
| T-NbAct*-*sg2-F | ATTGATTATGTCGACAGTGAACTC |  |
| T-NbAct*-*sg2-R | AAACGAGTTCACTGTCGACATAAT |  |
| T-NbEF1*-*sg1-F | ATTGAACTGGTGCCAAGGTTACCA |  |
| T-NbEF1*-*sg1-R | AAACTGGTAACCTTGGCACCAGTT |  |
| T-NbEF1*-*sg2-F | ATTGTTGACGATAATAGATAGCAC |  |
| T-NbEF1*-*sg2-R | AAACGTGCTATCTATTATCGTCAA |  |
| T-OsAct1-sg1-F | GGCGTTCCATTATCGCAGACGAG |  |
| T-OsAct1-sg1-R | AAACCTCGTCTGCGATAATGGAA |  |
| T-OsSPP2-sg1-F | GGCGGGGGTTTCCTTATGTTAAGG |  |
| T-OsSPP2-sg1-R | AAACCCTTAACATAAGGAAACCCC |  |
| **c. *NbPGK*::*Gus* KI constructs** | | |
| PGKDT1-BsF | ATATATGGTCTCGATTGGCCACATATCCACTGGTGGGTT | Construction of the *NbPGK* knockout system |
| PGKDT1-F0 | TGGCCACATATCCACTGGTGGGTTTTAGAGCTAGAAATAGC |  |
| PGKDT2-R0 | AACCTGATTATGCTGATCACCGCAATCTCTTAGTCGACTCTAC |  |
| PGKDT2-BsR | ATTATTGGTCTCGAAACCTGATTATGCTGATCACCGC |  |
| NbPGK-LHA-F | TGACGCATGGAATTCTGCTATTGTGGGTGGTTCAA | Construction of pEasy-TIR-*PGK-*Donor |
| NbPGK-LHA-R | TACAGGACGTAACATTGACACAGCAACAGGGGCAT |  |
| NbPGK-GUS-F | CCTGTTGCTGTGTCAATGTTACGTCCTGTAGAAAC |  |
| NbPGK-GUS-R | TTAGTACAAATTGTTTCATTGTTTGCCTCCCTGCT |  |
| NbPGK-RHA-F | GGAGGCAAACAATGAAACAATTTGTACTAATTCTT |  |
| NbPGK-RHA-R | CTTTCTAGGGAAGCTTTAACACGAGTTACTCAAAG |  |
| NbPGK-sg1-F | CATATCCACTGGAGGTGCTGCCAGTTTGGAGCT | Generation of pEasy-TIR-*PGK-*Donor* by site-directed mutagenesis |
| NbPGK-sg1-R | GCACCTCCAGTGGATATGTGGCTCATCACGCTA |  |
| NbPGK-sg2-F | TCTTTTTTCTGCGGTCATCAGCATAATCAGTGGTAA |  |
| NbPGK-sg2-R | GACCGCAGAAAAAAGAATTAGTACAAATTGTTTGAC |  |
| U6-*NbPGK*-D-F | TCAGACTTGGGATCCTGCTATTGTGGGTGGTTCAA | Construction of pEasy-TIR-Donor*-promoter |
| U6-*NbPGK*-D-R | CTTTCTAGGGAAGCTTTAACACGAGTTACTCAAAG |  |
| PGK-D-AntiU6-F | TGACGCATGGAATTCTGCTATTGTGGGTGGTTCAA | Construction of pEasy-TIR-Donor*- antiU6 |
| PGK-D-AntiU6-R | ATCAGACTTGGGATCCTAACACGAGTTACTCAAAG |  |
| PGK-D1-F | CTGTCAAACACTGATAGTTTAGCCACATATCCACTGGTGGTGGTGCTATTGTGGGTGGT | Amplification of the corresponding donor sequence |
| PGK-D1-R | TCGTTTCCCGCCTTCAGTTTCCACTGATTATGCTGATCACCGGTAACACGAGTTACTCA |  |
| PGK-D2-F | CTGTCAAACACTGATAGTTTAGCCACATATCCACTGGTGGTGGTTAACCCTAGAAAGAT |  |
| PGK-D2-R | TCGTTTCCCGCCTTCAGTTTCCACTGATTATGCTGATCACCGGTTAACATGCGTCAATT |  |
| NbPGK-D3-F | GTCAAACACTGATAGTTTAGCCACATATCCACTGGTGGTGGGACCAAGCCCGTTATTCT |  |
| NbPGK-D3-R | GTTTCCCGCCTTCAGTTTCCACTGATTATGCTGATCACCGGTAACACGAGTTACTCAAA |  |
| NbPGK-D4-F | GTCAAACACTGATAGTTTAGCCACATATCCACTGGTGGTGGTGCTATTGTGGGTGGTTC |  |
| NbPGK-D4-R | GTTTCCCGCCTTCAGTTTCCACTGATTATGCTGATCACCGGGACCAAGCCCGTTATTCT |  |
| **d. *NbTPR*::*Gus* KI constructs** | | |
| TPRDT1-BsF | ATATATGGTCTCGATTGGTAGCTACAGCGTCTATAAGTT | Construction of the *NbTPR* knockout system |
| TPRDT1-F0 | TGGTAGCTACAGCGTCTATAAGTTTTAGAGCTAGAAATAGC |  |
| TPRDT2-R0 | AACCTGTCGTCGCCGACTGACTCAATCTCTTAGTCGACTCTAC |  |
| TPRDT2-BsR | ATTATTGGTCTCGAAACCTGTCGTCGCCGACTGACTC |  |
| NbTPR-LHA-F | TGACGCATGGAATTCGAGTTCCATTCGGACGACGA | Construction of pEasy-TIR-*TPR-*Donor |
| NbTPR-LHA-R | ACAGGACGTAACATTGCGGTAATTGACGCTGTAGC |  |
| NbTPR-GUS-F | GCGTCAATTACCGCAATGTTACGTCCTGTAGAAACCC |  |
| NbTPR-GUS-R | GATTTTCTTTCAAGCTCATTGTTTGCCTCCCTGCTGC |  |
| NbTPR-RHA-F | GAGGCAAACAATGAGCTTGAAAGAAAATCATTATTC |  |
| NbTPR-RHA-R | TCTTTCTAGGGAAGCTTAGTAGTAAATCGCATTAGT |  |
| NbTPR-sg2-F | ATTTCTTGCGCTCTCCTCGCCGACTGACTAACCCTG | Generation of pEasy-TIR-*TPR-*Donor* by site-directed mutagenesis |
| NbTPR-sg2-R | GGAGAGCGCAAGAAATACAGAAGTCCTGTGCATCAG |  |
| NbTPR-D-AntiU6-F | TGACGCATGGAATTCGAGTTCCATTCGGACGACGA | Construction of pEasy-TIR-Donor*- antiU6 |
| NbTPR-D-AntiU6-R | ATCAGACTTGGGATCCAGTAGTAAATCGCATTAGT |  |
| TPR-D1-F | CTGTCAAACACTGATAGTTTCGTAGCTACAGCGTCTATAACGGGAGTTCCATTCGGACG | Amplification of the corresponding donor sequence |
| TPR-D1-R | TCGTTTCCCGCCTTCAGTTTTAGTCAGTCGGCGACGACAGCGGAGTAGTAAATCGCATT |  |
| TPR-D2-F | CTGTCAAACACTGATAGTTTCGTAGCTACAGCGTCTATAACGGTTAACCCTAGAAAGAT |  |
| TPR-D2-R | TCGTTTCCCGCCTTCAGTTTTAGTCAGTCGGCGACGACAGCGGTTAACATGCGTCAATT |  |
| NbTPR-D4-F | GTCAAACACTGATAGTTTCGTAGCTACAGCGTCTATAACGGGAGTTCCATTCGGACGAC |  |
| NbTPR-D4-R | GTTTCCCGCCTTCAGTTTTAGTCAGTCGGCGACGACAGCGGGACCAAGCCCGTTATTCT |  |
| **e. *NbActin*::Gus KI constructs** | | |
| NbActin2 DT1-BsF | ATATATGGTCTCGATTGACTTCCTGTGGACAATGGAGTT | Construction of the *NbActin* knockout system |
| NbActin2 DT1-F0 | TGACTTCCTGTGGACAATGGAGTTTTAGAGCTAGAAATAGC |  |
| NbActin2 DT2-R0 | AACGAGTTCACTGTCGACATAACAATCTCTTAGTCGACTCTAC |  |
| NbActin2 DT2-BsR | ATTATTGGTCTCGAAACGAGTTCACTGTCGACATAAC |  |
| NbActin-F | TATGCTAGTGGTCGTACAACTG | Construction of pEasy-*NbAct* |
| NbActin-R | CACACTTCACTTAACAAGGACATG |  |
| NbActin-sg1-F tac | GACGAGTCTGGTCCATCTATCGTACACAGGAAGTGCTTCTAAGG | Point mutation construction of pEasy-*NbAct** |
| NbActin-sg1-R tac | TACGATAGATGGACCAGATTCATCATACTCACCCTTTGAAAT |  |
| NbActin-sg2-F ac | ATTATGTCGACAGTGAACACACGTTTGCTGTCGTAGACGAGTCT |  |
| NbActin-sg2-R ac | CGTGTGTTCACTGTCGACATAATAGCAAAAGCAGAAAAACAAAAAG |  |
| NbActin2-LHA-F | AAAATTGACGCATGGAATTCTCTGAAATTATATCTTATGACAG | Construction of pEasy-TIR-*NbAct-*Donor* |
| NbActin2 LHA-T2A-1R | ACGTCACCGCATGTTAGAAGACTTCCTCTGCCCTCGAAGCACTTCCTGTGTACGATAG |  |
| NbActin2 LHA-T2A-2R | GTTTCTACAGGACGTAACATAGGGCCGGGATTCTCCTCCACGTCACCGCATGTTAGAA |  |
| T2A-GUS-F | TGGAGGAGAATCCCGGCCCTATGTTACGTCCTGTAGAAAC |  |
| NbActin2-RHA-F | CAGCAGGGAGGCAAACAATGAGGTGCTGCTGCTGCTATTTTTA |  |
| NbActin2-RHA-R | TTATCTTTCTAGGGAAGCTTCTACCCTTGAATAGGGAGGCT |  |
| NbAct-D-AntiU6-F | AAAATTGACGCATGGAATTCTCTGAAATTATATCTTATGACAG | Construction of pEasy-TIR-Donor*- antiU6 |
| NbAct-D-AntiU6-R | GCTGCATCAGACTTGGGATCCCTACCCTTGAATAGGGAGGCT |  |
| NbAct-D1-F | TGTCAAACACTGATAGTTTCCATCCATTGTCCACAGGAAGTTCTGAAATTATATCTTAT | Amplification of the corresponding donor sequence |
| NbAct-D1-R | CGTTTCCCGCCTTCAGTTTCCTGAGTTCACTGTCGACATAACTACCCTTGAATAGGGAG |  |
| NbAct-D2-F | TGTCAAACACTGATAGTTTCCATCCATTGTCCACAGGAAGTTTAACCCTAGAAAGATAG |  |
| NbAct-D2-R | CGTTTCCCGCCTTCAGTTTCCTGAGTTCACTGTCGACATAATTAACATGCGTCAATTTT |  |
| NbAct-D4-F | TGTCAAACACTGATAGTTTCCATCCATTGTCCACAGGAAGTTCTGAAATTATATCTTAT |  |
| NbAct-D4-R | CGTTTCCCGCCTTCAGTTTCCTGAGTTCACTGTCGACATAAAAGCTTGACCAAGCCCGT |  |
| **f. *NbEF1α*::Gus KI constructs** | | |
| NbEF1*α* DT1-BsF | ATATATGGTCTCGATTGACTGGTGCCAAGGTTACCAGTT | Construction of the *NbEF1α* knockout system |
| NbEF1*α* DT1-F0 | TGACTGGTGCCAAGGTTACCAGTTTTAGAGCTAGAAATAGC |  |
| NbEF1*α* DT2-R0 | AACGTGCTATCTATTATCGTCAACAATCTCTTAGTCGACTCTAC |  |
| NbEF1*α* DT2-BsR | ATTATTGGTCTCGAAACGTGCTATCTATTATCGTCAAC |  |
| NbEF1*α*-F | TGACTGTGCTGTCCTGATTATCGA | Construction of pEasy-*NbEF1α* |
| NbEF1*α*-R | CTAGAGTTAAGCTTGTACAATTTAAAGGTG |  |
| NbEF1*α*-sg1-F ga | CTGGTGCCAAGGTTACGAAAGCCGCCCAGAAGAAAAAGTGAA | Point mutation construction of pEasy-*NbEF1α** |
| NbEF1*α*-sg1-R ga | CTTTCGTAACCTTGGCACCAGTTGGGTCCTTCTTGTCAACACT |  |
| NbEF1*α*-sg2-F gc | TGTAATGCTGGTATTTCGAGTCCTATCTATTATCGTCAAGTGT |  |
| NbEF1*α*-sg2-R gc | GACTCGAAATACCAGCATTACACTGCACTGTTCACTTTTTCT |  |
| NbEF1*α*-LHA-F | AAAATTGACGCATGGAATTCGTTGTTTTGTGACTTGTGCAG | Construction of pEasy-TIR-*NbEF1α-Donor** |
| NbEF1*α*-LHA-T2A-1R | CCACGTCACCGCATGTTAGAAGACTTCCTCTGCCCTCCTTTTTCTTCTGGGCGGCTTT |  |
| NbEF1*α*-LHA-T2A-2R | GGTTTCTACAGGACGTAACATAGGGCCGGGATTCTCCTCCACGTCACCGCATGTTAGAA |  |
| T2A-GUS-F | TGGAGGAGAATCCCGGCCCTATGTTACGTCCTGTAGAAAC |  |
| NbEF1*α-*GUS-R | CCAGCATTACACTGCACTGTTCATTGTTTGCCTCCCTGCT |  |
| NbEF1*α-*RHA-F | AGCAGGGAGGCAAACAATGAACAGTGCAGTGTAATGCTGG |  |
| NbEF1*α-*RHA-R | TTATCTTTCTAGGGAAGCTTCTAGAGTTAAGCTTGTACAATTTAAAG |  |
| NbEF1*α*-LHA-F | AAAATTGACGCATGGAATTCGTTGTTTTGTGACTTGTGCAG |  |
| NbEF1*α*-D-AntiU6-F | AAAATTGACGCATGGAATTCGTTGTTTTGTGACTTGTGCAG | Construction of pEasy-TIR-Donor*- antiU6 |
| NbEF1*α*-D-AntiU6-R | GCTGCATCAGACTTGGGATCCCTAGAGTTAAGCTTGTACAATTTAAAG |  |
| NbEF1-D1-F | TGTCAAACACTGATAGTTTACTGGTGCCAAGGTTACCAAGGGTTGTTTTGTGACTTGTG | Amplification of the corresponding donor sequence |
| NbEF1-D1-R | CGTTTCCCGCCTTCAGTTTTTGACGATAATAGATAGCACTGGCTAGAGTTAAGCTTGTA |  |
| NbEF1-D2-F | TGTCAAACACTGATAGTTTACTGGTGCCAAGGTTACCAAGGTTAACCCTAGAAAGATAG |  |
| NbEF1-D2-R | CGTTTCCCGCCTTCAGTTTTTGACGATAATAGATAGCACTGGTTAACATGCGTCAATTT |  |
| NbEF1-D4-F | TGTCAAACACTGATAGTTTACTGGTGCCAAGGTTACCAAGGGTTGTTTTGTGACTTGTG |  |
| NbEF1-D4-R | CGTTTCCCGCCTTCAGTTTTTGACGATAATAGATAGCACTGGGACCAAGCCCGTTATTC |  |
| **g. *OsAct*::Gus KI constructs** | | |
| OsAct*-*MT1-BsF | ATATATGGTCTCGATTGTTGCAGATGTGGATTGCCAGTT | Construction of the *OsActin* knockout system |
| OsAct*-*MT1-F0 | TGTTGCAGATGTGGATTGCCAGTTTTAGAGCTAGAAATAGC |  |
| OsAct*-*MT2-R0 | AACTTGTGCACAGGAAATGCTTCAATCTCTTAGTCGACTCTAC |  |
| OsAct*-*MT2-BsR | ATTATTGGTCTCGAAACTTGTGCACAGGAAATGCTTC |  |
| OsAct-F | TACCACAGGTGAGCACATTCGAC | Construction of pEasy-*OsActin* |
| OsAct-R | GTCAAGTCAAAACGTACAGAAGGC |  |
| OsAct-M1-F | CGTTGCAGATGTGGATTGCGAAAGCTGAGTACGACGAGTCTGGC | Point mutation construction pEasy- *OsActin** |
| OsAct-M1-R | TTTCGCAATCCACATCTGCAACGGCAAGCAACATTGTAAGCAA |  |
| OsAct-M2-F | CGACGAGTCTGGCCCATCTATCGTGCACAGGAAATGCTTCTAAT |  |
| OsAct-M2-R | GATAGATGGGCCAGACTCGTCGTACTCAGCTTTCGCAATCCA |  |
| OsAct-LHA-F | AAAATTGACGCATGGAATTCGATGAGATTTAGTTCTGCAATCTTCAA | Construction of pEasy-TIR-*OsActin*-*Donor** (*GUSi*) |
| OsAct-LHA-R | TCCACGTCACCGCATGTTAGAAGACTTCCTCTGCCCTCGAAGCATTTCCTGTGCACGA |  |
| NbActin2 LHA-T2A-2R | GTTTCTACAGGACGTAACATAGGGCCGGGATTCTCCTCCACGTCACCGCATGTTAGAAG |  |
| T2A-GUS-F | TGGAGGAGAATCCCGGCCCTATGTTACGTCCTGTAGAAAC |  |
| OsAct-GUS-R | TAGCATTCTTGGGTCCGAAGAATCATTGTTTGCCTCCCTGCTG |  |
| OsAct-RHA-F | TTCTTCGGACCCAAGAATGCTAAGCCAAGAGGAGCTGTTA |  |
| OsAct-RHA-R | ATTATCTTTCTAGGGAAGCTTACACAACGCGCACACAAACCATA |  |
| OsAct-D-AntiU6-F | AAAATTGACGCATGGAATTCGATGAGATTTAGTTCTGCAATCTTCAA | Construction of pEasy-TIR-Donor*- antiU6 |
| OsAct-D-AntiU6-R | GCTGCATCAGACTTGGGATCCACACAACGCGCACACAAACCATA |  |
| OsAct-D1-F | GTCAAACACTGATAGTTTTTGCAGATGTGGATTGCCAAGGGATGAGATTTAGTTCTGCA | Amplification of the corresponding donor sequence |
| OsAct-D1-R | GTTTCCCGCCTTCAGTTTAAGCATTTCCTGTGCACAATGGACACAACGCGCACACAAAC |  |
| OsAct-D2-F | GTCAAACACTGATAGTTTTTGCAGATGTGGATTGCCAAGGTTAACCCTAGAAAGATAGT |  |
| OsAct-D2-R | GTTTCCCGCCTTCAGTTTAAGCATTTCCTGTGCACAATGGTTAACATGCGTCAATTTTA |  |
| OsAct-D4-F | GTCAAACACTGATAGTTTTTGCAGATGTGGATTGCCAAGGGATGAGATTTAGTTCTGCA |  |
| OsAct-D4-R | GTTTCCCGCCTTCAGTTTAAGCATTTCCTGTGCACAATGGGACCAAGCCCGTTATTCTG |  |
| **h. *OsAct*::DsRed KI constructs** | | |
| OsAct-LHA-F | AAAATTGACGCATGGAATTCGATGAGATTTAGTTCTGCAATCTTCAA | Construction of pEasy-TIR-*OsActin*-*Donor** (*DsRed*) |
| OsAct-LHA-R | TCCACGTCACCGCATGTTAGAAGACTTCCTCTGCCCTCGAAGCATTTCCTGTGCACGA |  |
| LHA-T2A-Red-R | GACGTTCTCGGAGGAGGCCATAGGGCCGGGATTCTCCTCCACGTCACCGCATGTTAGA |  |
| T2A-Red-F | TGGAGGAGAATCCCGGCCCTATGGCCTCCTCCGAGAACGT |  |
| OsAct-Red-R | AGCATTCTTGGGTCCGAAGAATTAACCCTGAGACTGTTGGACA |  |
| OsAct-RHA-F | TTCTTCGGACCCAAGAATGCTAAGCCAAGAGGAGCTGTTA |  |
| OsAct-RHA-R | ATTATCTTTCTAGGGAAGCTTACACAACGCGCACACAAACCATA |  |

**Supplementary sequences**

**Donor NbPGK-GUS: 5′HA-1001 bp; 3′HA-999 bp)**

**sgRNA-target1 (PAM); 5′HA(1002 bp);** **mutant stop codon; GUS; 3′HA(999 bp); sgRNA-target2 (PAM). Mutations to prevent sgRNA cleavage (PAM).**

AGCCACATATCCACTGGTGGTGGTGCTATTGTGGGTGGTTCAAAGGTTTCATCCAAGATTGGAGTGATCGAATCACTTTTAGAGAAATGTGATATATTGCTTTTGGGTGGAGGAATGATCTTTACCTTCTACAAGGCTCAGGGTCTTTCAGTTGGTTCCTCCTTGGTTGAGGAAGACAAACTAGAACTCGCTACATCACTCCTAGAGAAGGCCAAGGCGAAAGGAGTCAGTCTCTTGTTACCATCTGATGTTGTGATTGCAGATAAATTTGCTCCTGATGCAAACAGCAAGGTTTGCATGCTAAGTTTTCTCATATAAACCTATCTGACCTTAGAGCTTTTTGCTCTTGAGATTCTTTAGACTTTCCATCTGAAATCTGTACTGTAATTGGCTCTTAATATCAGAGTTTGTTACTTATGGATTGTGTTGAAAATGCAATTTTGTTTTGGTTACTGCAGATTGTGCCGGCATCTGCTATCCCAGATGGTTGGATGGGGTTGGACATTGGACCAGACTCTGTTAAGACTTTCAACGATGCCTTGGATACCACAAAAACAGTGATCTGGAATGGACCTATGGGGGTGTTTGAATTTGACAAGTTTGCTGTTGGAACAGAGGTACCAATTACCATTCTTCTCTTCATATTTGTTTTACCTTACCGAATGCTGAGCTTTATAAAAGAAATAAAAAAGGGAATAAAGCTGGTTTTACATAGCTTTAAAAGTAAAGGAAGAGGAATAATCTGGTTGGATATGTCACTTTGTGTGTTTACCTGAGAGTAAATAGTAATAAGAATGTTGTTGTGGTGATAGGCAATTGCAAAGAAGCTCGCGGACTTAAGTGGGAAAGGAGTGACAACTATCATTGGAGGTGGAGATTCTGTTGCAGCTGTTGAGAAAGTTGGAGTTGCTAGCGTGATG**AGCCACATATCCACTGGAGGTGC**TGCCAGTTTGGAGCTACTGGAAGGCAAGGTGCTCCCTGGTGTCGTTGCTCTAGATGAAGCAGATGCCCCTGTTGCTGTGTCAATGTTACGTCCTGTAGAAACCCCAACCCGTGAAATCAAAAAACTCGACGGCCTGTGGGCATTCAGTCTGGATCGCGAAAACTGTGGAATTGATCAGCGTTGGTGGGAAAGCGCGTTACAAGAAAGCCGGGCAATTGCTGTGCCAGGCAGTTTTAACGATCAGTTCGCCGATGCAGATATTCGTAATTATGCGGGCAACGTCTGGTATCAGCGCGAAGTCTTTATACCGAAAGGTAAGTCTTACTCTCTCTTTTTTGGTCTGTATTTTTAATTTTTTGAAGTATACTATTTGTACTGACGCTAATAATCTTTTTTCAGGTTGGGCAGGCCAGCGTATCGTGCTGCGTTTCGATGCGGTCACTCATTACGGCAAAGTGTGGGTCAATAATCAGGAAGTGATGGAGCATCAGGGCGGCTATACGCCATTTGAAGCCGATGTCACGCCGTATGTTATTGCCGGGAAAAGTGTACGTATCACCGTTTGTGTGAACAACGAACTGAACTGGCAGACTATCCCGCCGGGAATGGTGATTACCGACGAAAACGGCAAGAAAAAGCAGTCTTACTTCCATGATTTCTTTAACTATGCCGGAATCCATCGCAGCGTAATGCTCTACACCACGCCGAACACCTGGGTGGACGATATCACCGTGGTGACGCATGTCGCGCAAGACTGTAACCACGCGTCTGTTGACTGGCAGGTACTTCATGCTTCAACGTGTAACTTAAGAGATACTGTGTGAAATTTTATATTTCCATACATTTGCTTGACCTTTGCTTTTTGTCAATTTTTTTCCCCTTACAGGTGGTGGCCAATGGTGATGTCAGCGTTGAACTGCGTGATGCGGATCAACAGGTGGTTGCAACTGGACAAGGCACTAGCGGGACTTTGCAAGTGGTGAATCCGCACCTCTGGCAACCGGGTGAAGGTTATCTCTATGAACTGTGCGTCACAGCCAAAAGCCAGACAGAGTGTGATATCTACCCGCTTCGCGTCGGCATCCGGTCAGTGGCAGTGAAGGGCGAACAGTTCCTGATTAACCACAAACCGTTCTACTTTACTGGCTTTGGTCGTCATGAAGATGCGGACTTGCGTGGCAAAGGATTCGATAACGTGCTGATGGTGCACGACCACGCATTAATGGACTGGATTGGGGCCAACTCCTACCGTACCTCGCATTACCCTTACGCTGAAGAGATGCTCGACTGGGCAGATGAACATGGCATCGTGGTGATTGATGAAACTGCTGCTGTCGGCTTTAACCTCTCTTTAGGCATTGGTTTCGAAGCGGGCAACAAGCCGAAAGAACTGTACAGCGAAGAGGCAGTCAACGGGGAAACTCAGCAAGCGCACTTACAGGCGATTAAAGAGCTGATAGCGCGTGACAAAAACCACCCAAGCGTGGTGATGTGGAGTATTGCCAACGAACCGGATACCCGTCCGCAAGGTGCACGGGAATATTTCGCGCCACTGGCGGAAGCAACGCGTAAACTCGACCCGACGCGTCCGATCACCTGCGTCAATGTAATGTTCTGCGACGCTCACACCGATACCATCAGCGATCTCTTTGATGTGCTGTGCCTGAACCGTTATTACGGATGGTATGTCCAAAGCGGCGATTTGGAAACGGCAGAGAAGGTACTGGAAAAAGAACTTCTGGCCTGGCAGGAGAAACTGCATCAGCCGATTATCATCACCGAATACGGCGTGGATACGTTAGCCGGGCTGCACTCAATGTACACCGACATGTGGAGTGAAGAGTATCAGTGTGCATGGCTGGATATGTATCACCGCGTCTTTGATCGCGTCAGCGCCGTCGTCGGTGAACAGGTATGGAATTTCGCCGATTTTGCGACCTCGCAAGGCATATTGCGCGTTGGCGGTAACAAGAAAGGGATCTTCACTCGCGACCGCAAACCGAAGTCGGCGGCTTTTCTGCTGCAAAAACGCTGGACTGGCATGAACTTCGGTGAAAAACCGCAGCAGGGAGGCAAACAATGAAACAATTTGTACTAATTCTTTTTTCT**GCGGTCATCAGCATAATCAGTGG**TAATTTCCAGTTGGGAAGCATTGAGTTGATGTTGTAGATTTTTCAGGTTATATTGTTATATAATGTCCCCTTTCTTTAACCCATGTTATTTTGTCTAAATAAAGGGCGAGTATATCAGTTATAGACAGCTATCCTTTTGATGTCCTTCAACAAACTCTATCCTTGATTTGGTTCAGTTTGGAGGAACTTCTTTAGACATAAGAACTTTTGCCATGTGAACAAACTCATGCTGCGTGTTAATGTTCACCTGCCCTTCGCATTAAATGACGCCTCTAATACGTGGTGGTCTATGTAAAGTGAGATTACTGTTAGTTATGCAGTTATGAAACTTCTGGAAAACTTGAGACACCTGATCTTTGATTTTCAAAATATAAACCCATAATTTTCGTGAAGCATCATCAATAAAAGTAACAAAATATTTGTTACCGTACATTTATTCAATTTCCATTGGGCCACAAACATCAGAATATACTAAATCAAGTATATTCAATTTTCTTTCAAACGATGTCTGAAATGAGACTCTATGCTGCTTACCAAATAAACAGTAGTCACAGGGTTTTATTGTTGTACCTTTGGCATAAGAAATAAGTGATTTTTTGGCAAGAATCTGCAATCCCTTGTCGCTCATATGACCCATTCTTTTTATGCCACAAATTTGCAGAAATCTCATCTTGCATCGCATTCAATTCACCTTGGCATATTACTGCATTTGTCCTGTACAACGTGCCACGAGTAACTCCCTTTGCAATCACCAACGATCCCTTGGTGAGTCTCCATTTTTGATTTGCAAAATAGTTCTCGTATCCATCTCGGTCCAAAGTAATCCCCGAGATCAAGTTCATCCACAAAACAGGTACATGCCGCACATCCTTTAGAACCAATGTGCATCCGACATTTGTCTTGATACAAATGTCACCAATCCCCCTGCAATCTTTGAGTAACTCGTGTTACCGGTGATCAGCATAATCAGTGG

**Donor NbTPR-GUS: 5′HA-896 bp; 3′HA-1000 bp**

**sgRNA-target1 (PAM); 5′HA(896 bp); GUS; 3′HA(1000 bp); sgRNA-target2 (PAM). Mutations to prevent sgRNA cleavage (PAM).**

CGTAGCTACAGCGTCTATAACGGGAGTTCCATTCGGACGACGACAGCTATGATGAGGAAGATGAAGATAATGACTGTGGTTACATTAGTAAGCAGTACAGTTTGGGAAATAATGGTGTAAAAGAGGAAATGAGTTATGTGAGTCAAAATTCAAAGTTAGAAATTGTTGAAGGCAGAGAAGAAATGTACCTAGCAAGAGGAATTGGGATTGCTGATATTGGTTGTTTTGATGATGGTGGCCCTTATGGAGGCTGGGGCAACGGAGGAGGAGGAGGCGGCGGTTATCCGCCAGTAGCCTTCGACAGAGAAGGTGGTGGCGACAGTCAGGGGCTCCACATAGAAGAGTATTACAAGAGGATGTTGGAACAGAATCCTGGCAATTCCCTATTCTTGAGGAATTATGCCAACTTTTTATACCAAGTGAGTCTTTAACCTTCCTCAAGCTACTCTTATTTAGCATATTTTACAGGCATCTTGTGAATCCACAATAGCTAACTTTGCTTTTGCTAATGTTGGGGCAGACAAAGAAAGATCTTAAGGGGGCAGAGGAATACTACTCAAGAGCTATTTTAGCAGATCCAAGTGATGGTGAAATTCTTTCACAGTACGCTAAACTAATATGGGAGCTTCACCGCGATGAAGATAGAGCCACGAGCTATTTTGAAAGAGCAGTTCAAGCTGCCTCTAGTGACAGGTTCGCTTCTCATACACTACTGAGAATAAGCTTTATTATAGAAAATACAATTCAAGAACGGTGTTATTTATTTCAAGAACTGACCTCTTCATCTTTGATGTGCTGACAGCCACATACATGCAGCTTATGCTAATTTCCTCTGGGAGATAGAAGATGAAGAAGATGAGAATGATGATATACAAGCACCACGAATGCTGCATAC**CGTAGCTACAGCGTCAATTACCG**CAATGTTACGTCCTGTAGAAACCCCAACCCGTGAAATCAAAAAACTCGACGGCCTGTGGGCATTCAGTCTGGATCGCGAAAACTGTGGAATTGATCAGCGTTGGTGGGAAAGCGCGTTACAAGAAAGCCGGGCAATTGCTGTGCCAGGCAGTTTTAACGATCAGTTCGCCGATGCAGATATTCGTAATTATGCGGGCAACGTCTGGTATCAGCGCGAAGTCTTTATACCGAAAGGTAAGTCTTACTCTCTCTTTTTTGGTCTGTATTTTTAATTTTTTGAAGTATACTATTTGTACTGACGCTAATAATCTTTTTTCAGGTTGGGCAGGCCAGCGTATCGTGCTGCGTTTCGATGCGGTCACTCATTACGGCAAAGTGTGGGTCAATAATCAGGAAGTGATGGAGCATCAGGGCGGCTATACGCCATTTGAAGCCGATGTCACGCCGTATGTTATTGCCGGGAAAAGTGTACGTATCACCGTTTGTGTGAACAACGAACTGAACTGGCAGACTATCCCGCCGGGAATGGTGATTACCGACGAAAACGGCAAGAAAAAGCAGTCTTACTTCCATGATTTCTTTAACTATGCCGGAATCCATCGCAGCGTAATGCTCTACACCACGCCGAACACCTGGGTGGACGATATCACCGTGGTGACGCATGTCGCGCAAGACTGTAACCACGCGTCTGTTGACTGGCAGGTACTTCATGCTTCAACGTGTAACTTAAGAGATACTGTGTGAAATTTTATATTTCCATACATTTGCTTGACCTTTGCTTTTTGTCAATTTTTTTCCCCTTACAGGTGGTGGCCAATGGTGATGTCAGCGTTGAACTGCGTGATGCGGATCAACAGGTGGTTGCAACTGGACAAGGCACTAGCGGGACTTTGCAAGTGGTGAATCCGCACCTCTGGCAACCGGGTGAAGGTTATCTCTATGAACTGTGCGTCACAGCCAAAAGCCAGACAGAGTGTGATATCTACCCGCTTCGCGTCGGCATCCGGTCAGTGGCAGTGAAGGGCGAACAGTTCCTGATTAACCACAAACCGTTCTACTTTACTGGCTTTGGTCGTCATGAAGATGCGGACTTGCGTGGCAAAGGATTCGATAACGTGCTGATGGTGCACGACCACGCATTAATGGACTGGATTGGGGCCAACTCCTACCGTACCTCGCATTACCCTTACGCTGAAGAGATGCTCGACTGGGCAGATGAACATGGCATCGTGGTGATTGATGAAACTGCTGCTGTCGGCTTTAACCTCTCTTTAGGCATTGGTTTCGAAGCGGGCAACAAGCCGAAAGAACTGTACAGCGAAGAGGCAGTCAACGGGGAAACTCAGCAAGCGCACTTACAGGCGATTAAAGAGCTGATAGCGCGTGACAAAAACCACCCAAGCGTGGTGATGTGGAGTATTGCCAACGAACCGGATACCCGTCCGCAAGGTGCACGGGAATATTTCGCGCCACTGGCGGAAGCAACGCGTAAACTCGACCCGACGCGTCCGATCACCTGCGTCAATGTAATGTTCTGCGACGCTCACACCGATACCATCAGCGATCTCTTTGATGTGCTGTGCCTGAACCGTTATTACGGATGGTATGTCCAAAGCGGCGATTTGGAAACGGCAGAGAAGGTACTGGAAAAAGAACTTCTGGCCTGGCAGGAGAAACTGCATCAGCCGATTATCATCACCGAATACGGCGTGGATACGTTAGCCGGGCTGCACTCAATGTACACCGACATGTGGAGTGAAGAGTATCAGTGTGCATGGCTGGATATGTATCACCGCGTCTTTGATCGCGTCAGCGCCGTCGTCGGTGAACAGGTATGGAATTTCGCCGATTTTGCGACCTCGCAAGGCATATTGCGCGTTGGCGGTAACAAGAAAGGGATCTTCACTCGCGACCGCAAACCGAAGTCGGCGGCTTTTCTGCTGCAAAAACGCTGGACTGGCATGAACTTCGGTGAAAAACCGCAGCAGGGAGGCAAACAATGAGCTTGAAAGAAAATCATTATTCAAATGTTTAACAGCAGTATTAGCTGAAGTTTGGTGAGCAATTATGAAGAACCTGTAATTTAGCCTCTAAGGTAGTAATAAAACTGATGCACAGGACTTCTGTATTTCTT**GCGCTCTCCTCGCCGACTGACTA**ACCCTGTGAATGGTGAAACAGAAGCACTTGTCAAACTCCAATTGTGCTAAGCGTTTATATTGACAAGTTCTTCGCGACTTTCATTGCTTGCAAAACTTACATGTTTAATACCATATTACAATCAGGAGAGTTGGGGGACATGGATTATGAAGAAGCAGCTTGACGTCACCACCATACCGTTTAGCTTTGCTCAAGACATTCTGACATGCTGCATTGAACAACAAAAATAACAGTATTTCATCTTGGATTGTAGTACCATCATCGTAGGGAGGAATTAGCATTAACTGTGACTAGGGCATTAGTCCAAATGAAACAGGAAATCTTCGTACACAGCCTAACTAAAGAATCACGACCAAGAGATTATATCCTGCATACAAGGATAATGTTAATTGATTCAACCAAAGAACTACTCAAGCAAGCAAAAGATAGAACAAATCTTTAAGAAAAGCTTAAAGAGAAATAAGTACCACAATGTGAAATTGAAATTAATTTAAGCTACAGACTATCTCCACGAGAAACGGTACAGCTGATTGATCTACTTTCCTCAATGTAAACTTGCCTTCAAGCCAATGGCACGGGTAATGCCTAAGAACAGGGAAGAGAAAGCATCATATACTAGGTATACAACGGATGAGATAACAAAGTAATGTATGAATATGAGATTGTGGCAATGATGACGTACACCTTTCACTTGCAACATTACTCAAGACAACTAACTGCATCATATTTCTTCTGAAAAAATGTTTTATCTACTGAATGCAAGTTCCGCAACCATTAAATAAAAGATCATCGATCTATGTGATCAGAAATATTTCAAGCACGCACCTTACTAATGCGATTTACTACTCCGCTGTCGTCGCCGACTGACTA

**Donor NbActin-GUS: 5′HA-800 bp; 3′HA-800 bp**

**sgRNA-target1 (PAM); 5′HA(800 bp); T2A; GUS; 3′HA(1000 bp); sgRNA-target2 (PAM). Mutations to prevent sgRNA cleavage (PAM).**

CCATCCATTGTCCACAGGAAGTTCTGAAATTATATCTTATGACAGGTATTGTGTTGGATTCTGGTGATGGTGTGAGTCACACTGTCCCCATCTACGAGGGTTATGCTTTGCCTCATGCCATTCTTCGTTTGGACCTTGCTGGCCGTGACCTAACTGATAACCTGATGAAGATCCTCACTGAGAGAGGTTACATGTTCACCACCACTGCTGAACGGGAAATTGTCCGTGACATGAAGGAGAAGCTTGCTTATGTGGCTCTTGACTACGAGCAGGAGCTTGACACTGCCAAGAGCAGCTCCTCCGTCGAGAAGAACTATGAATTGCCTGATGGACAAGTTATTACCATTGGTGCTGAGAGGTTCCGTTGCCCAGAAGTCCTCTTCCAGCCATCCATGATCGGAATGGAAGCTGCAGGTATCCATGAGACTACCTACAACTCCATTATGAAGTGTGATGTTGATATCAGGAAGGACCTCTACGGTAACATTGTGCTCAGTGGTGGCTCAACCATGTTCCCCGGCATTGCTGATCGTATGAGCAAGGAAATCACTGCTTTGGCTCCTAGCAGCATGAAGATTAAGGTTGTTGCTCCACCGGAGAGAAAGTACAGTGTCTGGATAGGAGGATCCATCCTCGCATCCCTTAGCACATTCCAGCAGGCAAGCCTTATATATTTATCTTCACTTATTGTTTTGCCATTTTAATAAGCTGCATTGTATTGTGGTATTAAACTTTACTTGTTCTTCCATGCTTAATCAGATGTGGATTTCAAAGGGTGAGTACGACGAGTCTGGT**CCATCTATCGTACACAGGAAGT**GCTTCGAGGGCAGAGGAAGTCTTCTAACATGCGGTGACGTGGAGGAGAATCCCGGCCCTATGTTACGTCCTGTAGAAACCCCAACCCGTGAAATCAAAAAACTCGACGGCCTGTGGGCATTCAGTCTGGATCGCGAAAACTGTGGAATTGATCAGCGTTGGTGGGAAAGCGCGTTACAAGAAAGCCGGGCAATTGCTGTGCCAGGCAGTTTTAACGATCAGTTCGCCGATGCAGATATTCGTAATTATGCGGGCAACGTCTGGTATCAGCGCGAAGTCTTTATACCGAAAGGTAAGTCTTACTCTCTCTTTTTTGGTCTGTATTTTTAATTTTTTGAAGTATACTATTTGTACTGACGCTAATAATCTTTTTTCAGGTTGGGCAGGCCAGCGTATCGTGCTGCGTTTCGATGCGGTCACTCATTACGGCAAAGTGTGGGTCAATAATCAGGAAGTGATGGAGCATCAGGGCGGCTATACGCCATTTGAAGCCGATGTCACGCCGTATGTTATTGCCGGGAAAAGTGTACGTATCACCGTTTGTGTGAACAACGAACTGAACTGGCAGACTATCCCGCCGGGAATGGTGATTACCGACGAAAACGGCAAGAAAAAGCAGTCTTACTTCCATGATTTCTTTAACTATGCCGGAATCCATCGCAGCGTAATGCTCTACACCACGCCGAACACCTGGGTGGACGATATCACCGTGGTGACGCATGTCGCGCAAGACTGTAACCACGCGTCTGTTGACTGGCAGGTACTTCATGCTTCAACGTGTAACTTAAGAGATACTGTGTGAAATTTTATATTTCCATACATTTGCTTGACCTTTGCTTTTTGTCAATTTTTTTCCCCTTACAGGTGGTGGCCAATGGTGATGTCAGCGTTGAACTGCGTGATGCGGATCAACAGGTGGTTGCAACTGGACAAGGCACTAGCGGGACTTTGCAAGTGGTGAATCCGCACCTCTGGCAACCGGGTGAAGGTTATCTCTATGAACTGTGCGTCACAGCCAAAAGCCAGACAGAGTGTGATATCTACCCGCTTCGCGTCGGCATCCGGTCAGTGGCAGTGAAGGGCGAACAGTTCCTGATTAACCACAAACCGTTCTACTTTACTGGCTTTGGTCGTCATGAAGATGCGGACTTGCGTGGCAAAGGATTCGATAACGTGCTGATGGTGCACGACCACGCATTAATGGACTGGATTGGGGCCAACTCCTACCGTACCTCGCATTACCCTTACGCTGAAGAGATGCTCGACTGGGCAGATGAACATGGCATCGTGGTGATTGATGAAACTGCTGCTGTCGGCTTTAACCTCTCTTTAGGCATTGGTTTCGAAGCGGGCAACAAGCCGAAAGAACTGTACAGCGAAGAGGCAGTCAACGGGGAAACTCAGCAAGCGCACTTACAGGCGATTAAAGAGCTGATAGCGCGTGACAAAAACCACCCAAGCGTGGTGATGTGGAGTATTGCCAACGAACCGGATACCCGTCCGCAAGGTGCACGGGAATATTTCGCGCCACTGGCGGAAGCAACGCGTAAACTCGACCCGACGCGTCCGATCACCTGCGTCAATGTAATGTTCTGCGACGCTCACACCGATACCATCAGCGATCTCTTTGATGTGCTGTGCCTGAACCGTTATTACGGATGGTATGTCCAAAGCGGCGATTTGGAAACGGCAGAGAAGGTACTGGAAAAAGAACTTCTGGCCTGGCAGGAGAAACTGCATCAGCCGATTATCATCACCGAATACGGCGTGGATACGTTAGCCGGGCTGCACTCAATGTACACCGACATGTGGAGTGAAGAGTATCAGTGTGCATGGCTGGATATGTATCACCGCGTCTTTGATCGCGTCAGCGCCGTCGTCGGTGAACAGGTATGGAATTTCGCCGATTTTGCGACCTCGCAAGGCATATTGCGCGTTGGCGGTAACAAGAAAGGGATCTTCACTCGCGACCGCAAACCGAAGTCGGCGGCTTTTCTGCTGCAAAAACGCTGGACTGGCATGAACTTCGGTGAAAAACCGCAGCAGGGAGGCAAACAATGAGGTGCTGCTGCTGCTATTTTTACTTTGGTCGAGATTTTCCTTTTTGTTTTTCTGCTTTTGCTA**TTATGTCGACAGTGAACACACG**TTTGCTGTCGTAGACGAGTCTTTGAAGGTGGGGCTGTGAAGGAAGGGCGATAAAAAAACATTGTACTGCTGATACTGTTTCATGTTTACAATGCAATGTGGATTATATTCTGAGCACATTGAGAGAGTGCAAGCCTCCTTTGTCTGGAAATATCCCTTCTTGAACTTCTGTATGCTTGTAGTTGGGGAGAGATTGTGATGTTTTTCTTTGAGTCTTTTCCTTCATTTTCTCAGTGTTCTTGTTTTGTTATTCTATGGTACTTTTCTTTCTTGGGAACATATGTCAATATTTATTGAATGGGAATTTTTGGTAGTATTTTCAGTTTTCGGATAGTTCTAGTATTTTCTGTGGATGTGGGAATTGACATTTCTGTGGATCATTTTTGGTGCAGCGTTGTTTTCATCTCTGTTATGTATTCCTGTTGCAACCAGTGTCCTATTCCTCGTGGCGTTTTCAGCAAACGTATGAGGCTATAAAAAACAAAATGCCTTGCCCTTCCGGTTGGTGTACAAAATTTTGCTGTTGTACATATTTGGTTTCAACCCCCAAAATTTTGCTGTTGTATAAAATGACAAGAGTAGTTGCTATGATGGTAAGCAACCCCAGTTCCAACCGAGAGGTTGTGAGTTCGATTCTCCCCAAGAGCAAGGTGGGAAGTTCTTGGAGGGAAGTATGCCGGGGTCTATTCGGAAACAGCCTCCCTATTCAAGGGTAGTTATGTCGACAGTGAACTCAGG

**Donor NbEF1α-GUS: 5′HA-900 bp; 3′HA-874 bp**

**sgRNA-target1 (PAM); 5′HA(900 bp); T2A; GUS; 3′HA(874 bp); sgRNA-target2 (PAM). Mutations to prevent sgRNA cleavage (PAM).**

ACTGGTGCCAAGGTTACCAAGGGTTGTTTTGTGACTTGTGCAGATGGATGCTACCACACCCAAGTACTCCAAGGCTAGGTATGATGAAATCGTGAAGGAAGTTTCTTCCTACCTCAAGAAGGTTGGTTACAACCCTGACAAGATTCCCTTCGTCCCCATCTCTGGTTTTGAGGGGGACAATATGATTGAGAGGTCTACCAACCTTGACTGGTACAAGGGGCCAACCCTCCTTGAGGCTCTTGACCAGATTAATGAGCCCAAGAGGCCCACAGACAAACCCCTACGTCTTCCACTTCAGGACGTTTACAAGATTGGTGGTATTGGTACTGTCCCTGTTGGTCGTGTTGAAACTGGTGTCCTCAAGCCTGGTATGGTTGTGACCTTTGGACCTACTGGTCTGACAACTGAAGTCAAGTCTGTAGAGATGCACCACGAAGCTCTCCAGGAGGCACTCCCTGGTGACAATGTTGGGTTCAATGTTAAGAATGTTGCTGTTAAGGATCTCAAGCGTGGTTATGTTGCCTCAAACTCCAAGGATGACCCAGCCAAAGGGGCTGCCAGTTTCACCTCCCAGGTCATCATCATGAACCATCCTGGTCAGATTGGAAATGGATATGCACCAGTTCTTGACTGCCACACTTCCCACATTGCTGTCAAGTTCGCTGAGATCTTGACCAAGATTGACAGACGTTCTGGTAAGGAACTTGAGAAGGAGCCTAAGTTCTTGAAGAATGGTGATGCTGGTATGGTTAAGATGATGCCGACCAAGCCTATGGTTGTGGAGACCTTTGCCGAGTACCCACCATTGGGTCGTTTTGCTGTGAGGGACATGCGCCAGACTGTTGCTGTTGGTGTCATCAAGAGTGTTGACAAGAAGGACCCA**ACTGGTGCCAAGGTTACGAAAG**CCGCCCAGAAGAAAAAGGAGGGCAGAGGAAGTCTTCTAACATGCGGTGACGTGGAGGAGAATCCCGGCCCTATGTTACGTCCTGTAGAAACCCCAACCCGTGAAATCAAAAAACTCGACGGCCTGTGGGCATTCAGTCTGGATCGCGAAAACTGTGGAATTGATCAGCGTTGGTGGGAAAGCGCGTTACAAGAAAGCCGGGCAATTGCTGTGCCAGGCAGTTTTAACGATCAGTTCGCCGATGCAGATATTCGTAATTATGCGGGCAACGTCTGGTATCAGCGCGAAGTCTTTATACCGAAAGGTAAGTCTTACTCTCTCTTTTTTGGTCTGTATTTTTAATTTTTTGAAGTATACTATTTGTACTGACGCTAATAATCTTTTTTCAGGTTGGGCAGGCCAGCGTATCGTGCTGCGTTTCGATGCGGTCACTCATTACGGCAAAGTGTGGGTCAATAATCAGGAAGTGATGGAGCATCAGGGCGGCTATACGCCATTTGAAGCCGATGTCACGCCGTATGTTATTGCCGGGAAAAGTGTACGTATCACCGTTTGTGTGAACAACGAACTGAACTGGCAGACTATCCCGCCGGGAATGGTGATTACCGACGAAAACGGCAAGAAAAAGCAGTCTTACTTCCATGATTTCTTTAACTATGCCGGAATCCATCGCAGCGTAATGCTCTACACCACGCCGAACACCTGGGTGGACGATATCACCGTGGTGACGCATGTCGCGCAAGACTGTAACCACGCGTCTGTTGACTGGCAGGTACTTCATGCTTCAACGTGTAACTTAAGAGATACTGTGTGAAATTTTATATTTCCATACATTTGCTTGACCTTTGCTTTTTGTCAATTTTTTTCCCCTTACAGGTGGTGGCCAATGGTGATGTCAGCGTTGAACTGCGTGATGCGGATCAACAGGTGGTTGCAACTGGACAAGGCACTAGCGGGACTTTGCAAGTGGTGAATCCGCACCTCTGGCAACCGGGTGAAGGTTATCTCTATGAACTGTGCGTCACAGCCAAAAGCCAGACAGAGTGTGATATCTACCCGCTTCGCGTCGGCATCCGGTCAGTGGCAGTGAAGGGCGAACAGTTCCTGATTAACCACAAACCGTTCTACTTTACTGGCTTTGGTCGTCATGAAGATGCGGACTTGCGTGGCAAAGGATTCGATAACGTGCTGATGGTGCACGACCACGCATTAATGGACTGGATTGGGGCCAACTCCTACCGTACCTCGCATTACCCTTACGCTGAAGAGATGCTCGACTGGGCAGATGAACATGGCATCGTGGTGATTGATGAAACTGCTGCTGTCGGCTTTAACCTCTCTTTAGGCATTGGTTTCGAAGCGGGCAACAAGCCGAAAGAACTGTACAGCGAAGAGGCAGTCAACGGGGAAACTCAGCAAGCGCACTTACAGGCGATTAAAGAGCTGATAGCGCGTGACAAAAACCACCCAAGCGTGGTGATGTGGAGTATTGCCAACGAACCGGATACCCGTCCGCAAGGTGCACGGGAATATTTCGCGCCACTGGCGGAAGCAACGCGTAAACTCGACCCGACGCGTCCGATCACCTGCGTCAATGTAATGTTCTGCGACGCTCACACCGATACCATCAGCGATCTCTTTGATGTGCTGTGCCTGAACCGTTATTACGGATGGTATGTCCAAAGCGGCGATTTGGAAACGGCAGAGAAGGTACTGGAAAAAGAACTTCTGGCCTGGCAGGAGAAACTGCATCAGCCGATTATCATCACCGAATACGGCGTGGATACGTTAGCCGGGCTGCACTCAATGTACACCGACATGTGGAGTGAAGAGTATCAGTGTGCATGGCTGGATATGTATCACCGCGTCTTTGATCGCGTCAGCGCCGTCGTCGGTGAACAGGTATGGAATTTCGCCGATTTTGCGACCTCGCAAGGCATATTGCGCGTTGGCGGTAACAAGAAAGGGATCTTCACTCGCGACCGCAAACCGAAGTCGGCGGCTTTTCTGCTGCAAAAACGCTGGACTGGCATGAACTTCGGTGAAAAACCGCAGCAGGGAGGCAAACAATGAACAGTGCAGTGTAATGCTGGTATTT**CGAGTCCTATCTATTATCGTCAA**GTGTGTTGTGTAGACTCTGTTTTATTCAGTTCATCTATAGTTTTGTCTTTGATTTTAGGTTCCAGTTCTCTGCCTTGTATGTCAGGTAGCCATTCTCAGAACTGGGTACTCGACAGGCGGTGGCGAGCATTACTTTGAGTCTGTCTTCATTGTGTTGAGGAGGTGCCTTGGATTTCCATGGCAGCTCAGTATTTTTTCTATTTATGTTGCCTTAAGTACTTAATTTGAGCTTTTTAAAAATTTCTACTTTTTGCCTTCTTTGAGTGGAAGATTAGATGCCTTATACAATGCGTTTGTTGAAGATACTTTATTTGTTAAAGTAGGGGACGTCCAATGCTGAGCTCATATTTAAACTCTAGTCATGTGGTGGTTTTTCTTACTCTCTTTGTTAGGGTATGCAAGGATTGAGTGTGTCAAAAACCCACAATTTATATTAGTGTACGCCAAGGGTTTTTGGCACTGCAATGCACCATGTGTTAAACCACACATAATTTATGTATTATCACTACTTTAACCTTGTATAGCTCAAGATTCTCATACCAACTTCAGAGAGGTGCGTTCTGATAGGTCTTATCCCCTCCCTCAGTACCGTGATAGCACAAAATATTTCACTTGTTAGGCAAGCCAACGTTAGTTATATTTATTAATCATTTTTAACAATTTTTAAATTAAATGATAATCCAATTAGAATGCTTGGTACTTTGTAGTTGTACATAAGCAAAGTATTGAAAATCATATTTACTCTTTCTATTTCTAAGCTGTGATGCTTAGGTTTGGTTTTTCAATATTGTGTAAGCACCTTTAAATTGTACAAGCTTAACTCTAGCCAGTGCTATCTATTATCGTCAA

**Donor OsActin-GUS: 5′HA-941 bp; 3′HA-962 bp**

**sgRNA-target1 (PAM); 5′HA(900 bp); T2A; GUS; 3′HA(874 bp); sgRNA-target2 (PAM). Mutations to prevent sgRNA cleavage (PAM).**

TTGCAGATGTGGATTGCCAAGGGATGAGATTTAGTTCTGCAATCTTCAATTGTCATACAGCAAGACTATATAATAGCTTTCAAAATAAAATCATAGGCAGTTCTCATAAATGGAATCATGTTTGAACATCCTAATTCTGTTGGCATGGAGTGCTTTGACATTTTGATGTGCTACAGTTGTGAATAACTGAATTTCCTTTTCCCAGGTATTGTGTTGGACTCTGGTGATGGTGTCAGCCACACTGTCCCCATCTATGAAGGATATGCTCTCCCCCATGCTATCCTTCGTCTCGACCTTGCTGGGCGTGATCTCACTGATTACCTCATGAAGATCCTGACGGAGCGTGGTTACTCATTCACCACAACGGCCGAGCGGGAAATTGTGAGGGACATGAAGGAGAAGCTTTCCTACATCGCCCTGGACTATGACCAGGAAATGGAGACTGCCAAGACCAGCTCCTCCGTGGAGAAGAGCTACGAGCTTCCTGATGGACAGGTTATCACCATTGGTGCTGAGCGTTTCCGCTGCCCTGAGGTCCTCTTCCAGCCTTCCTTCATAGGAATGGAAGCTGCGGGTATCCATGAGACTACATACAACTCCATCATGAAGTGCGACGTGGATATTAGGAAGGATCTATATGGCAACATCGTTCTCAGTGGTGGTACCACTATGTTCCCTGGCATTGCTGACAGGATGAGCAAGGAGATCACTGCCTTGGCTCCTAGCAGCATGAAGATCAAGGTGGTCGCCCCTCCTGAAAGGAAGTACAGTGTCTGGATTGGAGGATCCATCTTGGCATCTCTCAGCACATTCCAGCAGGTAAATATACAAATGCAGCAATGTAGTGTGTTTACCTCATGAACTTGATCAATTTGCTTACAATGTTGCTTGCCG**TTGCAGATGTGGATTGCGAAAG**CTGAGTACGACGAGTCTGGCCCAT**CTATCGTGCACAGGAAATGCTT**CGAGGGCAGAGGAAGTCTTCTAACATGCGGTGACGTGGAGGAGAATCCCGGCCCTATGTTACGTCCTGTAGAAACCCCAACCCGTGAAATCAAAAAACTCGACGGCCTGTGGGCATTCAGTCTGGATCGCGAAAACTGTGGAATTGATCAGCGTTGGTGGGAAAGCGCGTTACAAGAAAGCCGGGCAATTGCTGTGCCAGGCAGTTTTAACGATCAGTTCGCCGATGCAGATATTCGTAATTATGCGGGCAACGTCTGGTATCAGCGCGAAGTCTTTATACCGAAAGGTAAGTCTTACTCTCTCTTTTTTGGTCTGTATTTTTAATTTTTTGAAGTATACTATTTGTACTGACGCTAATAATCTTTTTTCAGGTTGGGCAGGCCAGCGTATCGTGCTGCGTTTCGATGCGGTCACTCATTACGGCAAAGTGTGGGTCAATAATCAGGAAGTGATGGAGCATCAGGGCGGCTATACGCCATTTGAAGCCGATGTCACGCCGTATGTTATTGCCGGGAAAAGTGTACGTATCACCGTTTGTGTGAACAACGAACTGAACTGGCAGACTATCCCGCCGGGAATGGTGATTACCGACGAAAACGGCAAGAAAAAGCAGTCTTACTTCCATGATTTCTTTAACTATGCCGGAATCCATCGCAGCGTAATGCTCTACACCACGCCGAACACCTGGGTGGACGATATCACCGTGGTGACGCATGTCGCGCAAGACTGTAACCACGCGTCTGTTGACTGGCAGGTACTTCATGCTTCAACGTGTAACTTAAGAGATACTGTGTGAAATTTTATATTTCCATACATTTGCTTGACCTTTGCTTTTTGTCAATTTTTTTCCCCTTACAGGTGGTGGCCAATGGTGATGTCAGCGTTGAACTGCGTGATGCGGATCAACAGGTGGTTGCAACTGGACAAGGCACTAGCGGGACTTTGCAAGTGGTGAATCCGCACCTCTGGCAACCGGGTGAAGGTTATCTCTATGAACTGTGCGTCACAGCCAAAAGCCAGACAGAGTGTGATATCTACCCGCTTCGCGTCGGCATCCGGTCAGTGGCAGTGAAGGGCGAACAGTTCCTGATTAACCACAAACCGTTCTACTTTACTGGCTTTGGTCGTCATGAAGATGCGGACTTGCGTGGCAAAGGATTCGATAACGTGCTGATGGTGCACGACCACGCATTAATGGACTGGATTGGGGCCAACTCCTACCGTACCTCGCATTACCCTTACGCTGAAGAGATGCTCGACTGGGCAGATGAACATGGCATCGTGGTGATTGATGAAACTGCTGCTGTCGGCTTTAACCTCTCTTTAGGCATTGGTTTCGAAGCGGGCAACAAGCCGAAAGAACTGTACAGCGAAGAGGCAGTCAACGGGGAAACTCAGCAAGCGCACTTACAGGCGATTAAAGAGCTGATAGCGCGTGACAAAAACCACCCAAGCGTGGTGATGTGGAGTATTGCCAACGAACCGGATACCCGTCCGCAAGGTGCACGGGAATATTTCGCGCCACTGGCGGAAGCAACGCGTAAACTCGACCCGACGCGTCCGATCACCTGCGTCAATGTAATGTTCTGCGACGCTCACACCGATACCATCAGCGATCTCTTTGATGTGCTGTGCCTGAACCGTTATTACGGATGGTATGTCCAAAGCGGCGATTTGGAAACGGCAGAGAAGGTACTGGAAAAAGAACTTCTGGCCTGGCAGGAGAAACTGCATCAGCCGATTATCATCACCGAATACGGCGTGGATACGTTAGCCGGGCTGCACTCAATGTACACCGACATGTGGAGTGAAGAGTATCAGTGTGCATGGCTGGATATGTATCACCGCGTCTTTGATCGCGTCAGCGCCGTCGTCGGTGAACAGGTATGGAATTTCGCCGATTTTGCGACCTCGCAAGGCATATTGCGCGTTGGCGGTAACAAGAAAGGGATCTTCACTCGCGACCGCAAACCGAAGTCGGCGGCTTTTCTGCTGCAAAAACGCTGGACTGGCATGAACTTCGGTGAAAAACCGCAGCAGGGAGGCAAACAATGATTCTTCGGACCCAAGAATGCTAAGCCAAGAGGAGCTGTTATCGCCGTCCTCCTGCTTGTTTCTCTCTTTTTGTTGCTGTTTCTTCATTAGCGTGGACAAAGTTTTCAACCGGCCTATCTGTTATCATTTTCTTCTATTCAAAGACTGTAATACCTATTGCTACCTGTGGTTCTCACTTGTGATTTTGGACACATATGTTCGGTTTATTCAAATTTAATCAGATGCCTGATGAGGGTACCAGAAAAAATACGTGTTCTGGTTGTTTTTGAGTTGCGATTATTCTATGAAATGAATAACATCGAAGTTATCATCCCAGTATTTTCGCATGAATGTTCTTTTCTTCTGTCTTGTGCATCAGTGATCTAGTGCATGGGAGTTTGTATTGTGATGTTCGACATCACGTAACTTCCACTTTGCCTTTGCTGTTCGATATTTTAATGACATGTCACACACACTTCTGATACTTTTCTTTCTTGGCTATTGTGCCAGCATGATGCAAGATGCATCACAGCATCAGATATATTCTCATCGTCAGGCTTTAGCAGCACACGAGCACGCTTTGCCGCTTAAAAGTTGTACGGCGCAGCTTAGACATCCCCTGTAGAAGTGATAATCTTTTCACTTTTCCTTAAACAAATTGAGAGGGGAAATGGAACCATGTGGATCAGAGAAGCTTTTGTTTCTTTACACAAGAATATTTGGTACAGTGGGGGTCCTATGTTCGTGGGTTCGTGGCTTGGCTGCCTGTCTTCAACCAAGTGTTTTCAGTTCAACATGTTAGCGTGTAGAAAGAGCACAATTCTGTTTATCTCCAAGGTAAAATGTGGCATTCTGTTAAAGAACATGATCCTGCCAATTTTTTAAGTTTCAATGGAAGAGGAATGTAAAGCTTTCTATGGTTTGTGTACACAACACAGTGGAAGAGGAGTGCAAGCTTTCTATGGTTTGTGTGCGCGTTGTGTCCATTGTGCACAGGAAATGCTT

**Donor OsActin-DsRed: 5′HA-941 bp; 3′HA-962 bp**

**sgRNA-target1 (PAM); 5′HA(900 bp); T2A; DsRed; 3′HA(874 bp); sgRNA-target2 (PAM). Mutations to prevent sgRNA cleavage (PAM).**

TTGCAGATGTGGATTGCCAAGGGATGAGATTTAGTTCTGCAATCTTCAATTGTCATACAGCAAGACTATATAATAGCTTTCAAAATAAAATCATAGGCAGTTCTCATAAATGGAATCATGTTTGAACATCCTAATTCTGTTGGCATGGAGTGCTTTGACATTTTGATGTGCTACAGTTGTGAATAACTGAATTTCCTTTTCCCAGGTATTGTGTTGGACTCTGGTGATGGTGTCAGCCACACTGTCCCCATCTATGAAGGATATGCTCTCCCCCATGCTATCCTTCGTCTCGACCTTGCTGGGCGTGATCTCACTGATTACCTCATGAAGATCCTGACGGAGCGTGGTTACTCATTCACCACAACGGCCGAGCGGGAAATTGTGAGGGACATGAAGGAGAAGCTTTCCTACATCGCCCTGGACTATGACCAGGAAATGGAGACTGCCAAGACCAGCTCCTCCGTGGAGAAGAGCTACGAGCTTCCTGATGGACAGGTTATCACCATTGGTGCTGAGCGTTTCCGCTGCCCTGAGGTCCTCTTCCAGCCTTCCTTCATAGGAATGGAAGCTGCGGGTATCCATGAGACTACATACAACTCCATCATGAAGTGCGACGTGGATATTAGGAAGGATCTATATGGCAACATCGTTCTCAGTGGTGGTACCACTATGTTCCCTGGCATTGCTGACAGGATGAGCAAGGAGATCACTGCCTTGGCTCCTAGCAGCATGAAGATCAAGGTGGTCGCCCCTCCTGAAAGGAAGTACAGTGTCTGGATTGGAGGATCCATCTTGGCATCTCTCAGCACATTCCAGCAGGTAAATATACAAATGCAGCAATGTAGTGTGTTTACCTCATGAACTTGATCAATTTGCTTACAATGTTGCTTGCCG**TTGCAGATGTGGATTGCGAAAG**CTGAGTACGACGAGTCTGGCCCAT**CTATCGTGCACAGGAAATGCTT**CGAGGGCAGAGGAAGTCTTCTAACATGCGGTGACGTGGAGGAGAATCCCGGCCCTATGGCCTCCTCCGAGAACGTCATCACCGAGTTCATGCGCTTCAAGGTGCGCATGGAGGGCACCGTGAACGGCCACGAGTTCGAGATCGAGGGCGAGGGCGAGGGCCGCCCCTACGAGGGCCACAACACCGTGAAGCTGAAGGTGACCAAGGGCGGCCCCCTGCCCTTCGCCTGGGACATCCTGTCCCCCCAGTTCCAGTACGGCTCCAAGGTGTACGTGAAGCACCCCGCCGACATCCCCGACTACAAGAAGCTGTCCTTCCCCGAGGGCTTCAAGTGGGAGCGCGTGATGAACTTCGAGGACGGCGGCGTGGCGACCGTGACCCAGGACTCCTCCCTGCAGGACGGCTGCTTCATCTACAAGGTGAAGTTCATCGGCGTGAACTTCCCCTCCGACGGCCCCGTGATGCAGAAGAAGACCATGGGCTGGGAGGCCTCCACCGAGCGCCTGTACCCCCGCGACGGCGTGCTGAAGGGCGAGACCCACAAGGCCCTGAAGCTGAAGGACGGCGGCCACTACCTGGTGGAGTTCAAGTCCATCTACATGGCCAAGAAGCCCGTGCAGCTGCCCGGCTACTACTACGTGGACGCCAAGCTGGACATCACCTCCCACAACGAGGACTACACCATCGTGGAGCAGTACGAGCGCACCGAGGGCCGCCACCACCTGTTCCTGGTACCAATGAGCTCTGTCCAACAGTCTCAGGGTTAATTCTTCGGACCCAAGAATGCTAAGCCAAGAGGAGCTGTTATCGCCGTCCTCCTGCTTGTTTCTCTCTTTTTGTTGCTGTTTCTTCATTAGCGTGGACAAAGTTTTCAACCGGCCTATCTGTTATCATTTTCTTCTATTCAAAGACTGTAATACCTATTGCTACCTGTGGTTCTCACTTGTGATTTTGGACACATATGTTCGGTTTATTCAAATTTAATCAGATGCCTGATGAGGGTACCAGAAAAAATACGTGTTCTGGTTGTTTTTGAGTTGCGATTATTCTATGAAATGAATAACATCGAAGTTATCATCCCAGTATTTTCGCATGAATGTTCTTTTCTTCTGTCTTGTGCATCAGTGATCTAGTGCATGGGAGTTTGTATTGTGATGTTCGACATCACGTAACTTCCACTTTGCCTTTGCTGTTCGATATTTTAATGACATGTCACACACACTTCTGATACTTTTCTTTCTTGGCTATTGTGCCAGCATGATGCAAGATGCATCACAGCATCAGATATATTCTCATCGTCAGGCTTTAGCAGCACACGAGCACGCTTTGCCGCTTAAAAGTTGTACGGCGCAGCTTAGACATCCCCTGTAGAAGTGATAATCTTTTCACTTTTCCTTAAACAAATTGAGAGGGGAAATGGAACCATGTGGATCAGAGAAGCTTTTGTTTCTTTACACAAGAATATTTGGTACAGTGGGGGTCCTATGTTCGTGGGTTCGTGGCTTGGCTGCCTGTCTTCAACCAAGTGTTTTCAGTTCAACATGTTAGCGTGTAGAAAGAGCACAATTCTGTTTATCTCCAAGGTAAAATGTGGCATTCTGTTAAAGAACATGATCCTGCCAATTTTTTAAGTTTCAATGGAAGAGGAATGTAAAGCTTTCTATGGTTTGTGTACACAACACAGTGGAAGAGGAGTGCAAGCTTTCTATGGTTTGTGTGCGCGTTGTGTCCATTGTGCACAGGAAATGCT

**pKSE-vCas9-ipB7^RKD^(*NbPGK*::*Gusi*)**

Right Border; Donor (sgRNA1-LE TIR-LHA-GOI-RHA-*U6*-RE TIR-sgRNA2); U6-26p-(Spacer1) gRNA scaffold-polyT-U6-29p-(Spacer2) gRNA scaffold-polyT; 2xCaMV35s-3xFLAG-NLS-vCas9-NLS-Linker-ipB7^RKD^- NLS; CaMV35s (enhanced)-Kana; Left Border

GTTTACCCGCCAATATATCCTGTCAAACACTGATAGTTTAGCCACATATCCACTGGTGGTGGTTAACCCTAGAAAGATAGTCTGCGTAAAATTGACGCATGGAATTCTGCTATTGTGGGTGGTTCAAAGGTTTCATCCAAGATTGGAGTGATCGAATCACTTTTAGAGAAATGTGATATATTGCTTTTGGGTGGAGGAATGATCTTTACCTTCTACAAGGCTCAGGGTCTTTCAGTTGGTTCCTCCTTGGTTGAGGAAGACAAACTAGAACTCGCTACATCACTCCTAGAGAAGGCCAAGGCGAAAGGAGTCAGTCTCTTGTTACCATCTGATGTTGTGATTGCAGATAAATTTGCTCCTGATGCAAACAGCAAGGTTTGCATGCTAAGTTTTCTCATATAAACCTATCTGACCTTAGAGCTTTTTGCTCTTGAGATTCTTTAGACTTTCCATCTGAAATCTGTACTGTAATTGGCTCTTAATATCAGAGTTTGTTACTTATGGATTGTGTTGAAAATGCAATTTTGTTTTGGTTACTGCAGATTGTGCCGGCATCTGCTATCCCAGATGGTTGGATGGGGTTGGACATTGGACCAGACTCTGTTAAGACTTTCAACGATGCCTTGGATACCACAAAAACAGTGATCTGGAATGGACCTATGGGGGTGTTTGAATTTGACAAGTTTGCTGTTGGAACAGAGGTACCAATTACCATTCTTCTCTTCATATTTGTTTTACCTTACCGAATGCTGAGCTTTATAAAAGAAATAAAAAAGGGAATAAAGCTGGTTTTACATAGCTTTAAAAGTAAAGGAAGAGGAATAATCTGGTTGGATATGTCACTTTGTGTGTTTACCTGAGAGTAAATAGTAATAAGAATGTTGTTGTGGTGATAGGCAATTGCAAAGAAGCTCGCGGACTTAAGTGGGAAAGGAGTGACAACTATCATTGGAGGTGGAGATTCTGTTGCAGCTGTTGAGAAAGTTGGAGTTGCTAGCGTGATGAGCCACATATCCACTGGAGGTGCTGCCAGTTTGGAGCTACTGGAAGGCAAGGTGCTCCCTGGTGTCGTTGCTCTAGATGAAGCAGATGCCCCTGTTGCTGTGTCAATGTTACGTCCTGTAGAAACCCCAACCCGTGAAATCAAAAAACTCGACGGCCTGTGGGCATTCAGTCTGGATCGCGAAAACTGTGGAATTGATCAGCGTTGGTGGGAAAGCGCGTTACAAGAAAGCCGGGCAATTGCTGTGCCAGGCAGTTTTAACGATCAGTTCGCCGATGCAGATATTCGTAATTATGCGGGCAACGTCTGGTATCAGCGCGAAGTCTTTATACCGAAAGGTAAGTCTTACTCTCTCTTTTTTGGTCTGTATTTTTAATTTTTTGAAGTATACTATTTGTACTGACGCTAATAATCTTTTTTCAGGTTGGGCAGGCCAGCGTATCGTGCTGCGTTTCGATGCGGTCACTCATTACGGCAAAGTGTGGGTCAATAATCAGGAAGTGATGGAGCATCAGGGCGGCTATACGCCATTTGAAGCCGATGTCACGCCGTATGTTATTGCCGGGAAAAGTGTACGTATCACCGTTTGTGTGAACAACGAACTGAACTGGCAGACTATCCCGCCGGGAATGGTGATTACCGACGAAAACGGCAAGAAAAAGCAGTCTTACTTCCATGATTTCTTTAACTATGCCGGAATCCATCGCAGCGTAATGCTCTACACCACGCCGAACACCTGGGTGGACGATATCACCGTGGTGACGCATGTCGCGCAAGACTGTAACCACGCGTCTGTTGACTGGCAGGTACTTCATGCTTCAACGTGTAACTTAAGAGATACTGTGTGAAATTTTATATTTCCATACATTTGCTTGACCTTTGCTTTTTGTCAATTTTTTTCCCCTTACAGGTGGTGGCCAATGGTGATGTCAGCGTTGAACTGCGTGATGCGGATCAACAGGTGGTTGCAACTGGACAAGGCACTAGCGGGACTTTGCAAGTGGTGAATCCGCACCTCTGGCAACCGGGTGAAGGTTATCTCTATGAACTGTGCGTCACAGCCAAAAGCCAGACAGAGTGTGATATCTACCCGCTTCGCGTCGGCATCCGGTCAGTGGCAGTGAAGGGCGAACAGTTCCTGATTAACCACAAACCGTTCTACTTTACTGGCTTTGGTCGTCATGAAGATGCGGACTTGCGTGGCAAAGGATTCGATAACGTGCTGATGGTGCACGACCACGCATTAATGGACTGGATTGGGGCCAACTCCTACCGTACCTCGCATTACCCTTACGCTGAAGAGATGCTCGACTGGGCAGATGAACATGGCATCGTGGTGATTGATGAAACTGCTGCTGTCGGCTTTAACCTCTCTTTAGGCATTGGTTTCGAAGCGGGCAACAAGCCGAAAGAACTGTACAGCGAAGAGGCAGTCAACGGGGAAACTCAGCAAGCGCACTTACAGGCGATTAAAGAGCTGATAGCGCGTGACAAAAACCACCCAAGCGTGGTGATGTGGAGTATTGCCAACGAACCGGATACCCGTCCGCAAGGTGCACGGGAATATTTCGCGCCACTGGCGGAAGCAACGCGTAAACTCGACCCGACGCGTCCGATCACCTGCGTCAATGTAATGTTCTGCGACGCTCACACCGATACCATCAGCGATCTCTTTGATGTGCTGTGCCTGAACCGTTATTACGGATGGTATGTCCAAAGCGGCGATTTGGAAACGGCAGAGAAGGTACTGGAAAAAGAACTTCTGGCCTGGCAGGAGAAACTGCATCAGCCGATTATCATCACCGAATACGGCGTGGATACGTTAGCCGGGCTGCACTCAATGTACACCGACATGTGGAGTGAAGAGTATCAGTGTGCATGGCTGGATATGTATCACCGCGTCTTTGATCGCGTCAGCGCCGTCGTCGGTGAACAGGTATGGAATTTCGCCGATTTTGCGACCTCGCAAGGCATATTGCGCGTTGGCGGTAACAAGAAAGGGATCTTCACTCGCGACCGCAAACCGAAGTCGGCGGCTTTTCTGCTGCAAAAACGCTGGACTGGCATGAACTTCGGTGAAAAACCGCAGCAGGGAGGCAAACAATGAAACAATTTGTACTAATTCTTTTTTCTGCGGTCATCAGCATAATCAGTGGTAATTTCCAGTTGGGAAGCATTGAGTTGATGTTGTAGATTTTTCAGGTTATATTGTTATATAATGTCCCCTTTCTTTAACCCATGTTATTTTGTCTAAATAAAGGGCGAGTATATCAGTTATAGACAGCTATCCTTTTGATGTCCTTCAACAAACTCTATCCTTGATTTGGTTCAGTTTGGAGGAACTTCTTTAGACATAAGAACTTTTGCCATGTGAACAAACTCATGCTGCGTGTTAATGTTCACCTGCCCTTCGCATTAAATGACGCCTCTAATACGTGGTGGTCTATGTAAAGTGAGATTACTGTTAGTTATGCAGTTATGAAACTTCTGGAAAACTTGAGACACCTGATCTTTGATTTTCAAAATATAAACCCATAATTTTCGTGAAGCATCATCAATAAAAGTAACAAAATATTTGTTACCGTACATTTATTCAATTTCCATTGGGCCACAAACATCAGAATATACTAAATCAAGTATATTCAATTTTCTTTCAAACGATGTCTGAAATGAGACTCTATGCTGCTTACCAAATAAACAGTAGTCACAGGGTTTTATTGTTGTACCTTTGGCATAAGAAATAAGTGATTTTTTGGCAAGAATCTGCAATCCCTTGTCGCTCATATGACCCATTCTTTTTATGCCACAAATTTGCAGAAATCTCATCTTGCATCGCATTCAATTCACCTTGGCATATTACTGCATTTGTCCTGTACAACGTGCCACGAGTAACTCCCTTTGCAATCACCAACGATCCCTTGGTGAGTCTCCATTTTTGATTTGCAAAATAGTTCTCGTATCCATCTCGGTCCAAAGTAATCCCCGAGATCAAGTTCATCCACAAAACAGGTACATGCCGCACATCCTTTAGAACCAATGTGCATCCGACATTTGTCTTGATACAAATGTCACCAATCCCCCTGCAATCTTTGAGTAACTCGTGTTAGGATCC*CAAGTCTGATGCAGCAAGCGAGTTTATAAACCGGTCAGAACTCTGCTGGACAGGCGAGGTGGGACTAAACTAAGAGGAACTGCTGCGTTGCTCTCCCCAATCGTGAACACGTCGACCACCCGACAAAGCCCGATCATGCGGGCCTAAAAGCCCAGCGCTAGACCGTTGCTGAACTCACGTGGGCTGCATCTCACTATGCGCCCAGATCAGTGAGAGCTAGAGGGCAGTTATCTTCAGCTCTCTCGTAGTTCCTCTGATCAATATTCCTAGTTCGATTCCCTCCTAGCAGTGAGTAACAATGTGCTCCTTGATAAATATAAATGTGTTGAGCACCAGAACTGTCAGAATAACGGGCTTGGTC*AAGCTTCCCTAGAAAGATAATCATATTGTGACGTACGTTAAAGATAATCATGCGTAAAATTGACGCATGTTAACCGGTGATCAGCATAATCAGTGGAAACTGAAGGCGGGAAACGACAATCTGATCCAAGCTCAAGCTAAGCTCACGTGACGGAATTAAGCTTCGACTTGCCTTCCGCACAATACATCATTTCTTCTTAGCTTTTTTTCTTCTTCTTCGTTCATACAGTTTTTTTTTGTTTATCAGCTTACATTTTCTTGAACCGTAGCTTTCGTTTTCTTCTTTTTAACTTTCCATTCGGAGTTTTTGTATCTTGTTTCATAGTTTGTCCCAGGATTAGAATGATTAGGCATCGAACCTTCAAGAATTTGATTGAATAAAACATCTTCATTCTTAAGATATGAAGATAATCTTCAAAAGGCCCCTGGGAATCTGAAAGAAGAGAAGCAGGCCCATTTATATGGGAAAGAACAATAGTATTTCTTATATAGGCCCATTTAAGTTGAAAACAATCTTCAAAAGTCCCACATCGCTTAGATAAGAAAACGAAGCTGAGTTTATATACAGCTAGAGTCGAAGTAGTGATTGGCCACATATCCACTGGTGGGTTTTAGAGCTAGAAATAGCAAGTTAAAATAAGGCTAGTCCGTTATCAACTTGAAAAAGTGGCACCGAGTCGGTGCTTTTTTTTGCAAAATTTTCCAGATCGATTTCTTCTTCCTCTGTTCTTCGGCGTTCAATTTCTGGGGTTTTCTCTTCGTTTTCTGTAACTGAAACCTAAAATTTGACCTAAAAAAAATCTCAAATAATATGATTCAGTGGTTTTGTACTTTTCAGTTAGTTGAGTTTTGCAGTTCCGATGAGATAAACCAATATTAATCCAAACTACTGCAGCCTGACAGACAAATGAGGATGCAAACAATTTTAAAGTTTATCTAACGCTAGCTGTTTTGTTTCTTCTCTCTGGTGCACCAACGACGGCGTTTTCTCAATCATAAAGAGGCTTGTTTTACTTAAGGCCAATAATGTTGATGGATCGAAAGAAGAGGGCTTTTAATAAACGAGCCCGTTTAAGCTGTAAACGATGTCAAAAACATCCCACATCGTTCAGTTGAAAATAGAAGCTCTGTTTATATATTGGTAGAGTCGACTAAGAGATTGCACTGATTATGCTGATCACGTTTTAGAGCTAGAAATAGCAAGTTAAAATAAGGCTAGTCCGTTATCAACTTGAAAAAGTGGCACCGAGTCGGTGCTTTTTTTTGCAAAATTTTCCAGATCGATTTCTTCTTCCTCTGTTCTTCGGCGTTCAATTTCTGGGGTTTTCTCTTCGTTTTCTGTAACTGAAACCTAAAATTTGACCTAAAAAAAATCTCAAATAATATGATTCAGTGGTTTTGTACTTTTCAGTTAGTTGAGTTTTGCAGTTCCGATGAGATAAACCAATAAGCTTGCATGCCTGCAGGTCAACATGGTGGAGCACGACACACTTGTCTACTCCAAAAATATCAAAGATACAGTCTCAGAAGACCAAAGGGCAATTGAGACTTTTCAACAAAGGGTAATATCCGGAAACCTCCTCGGATTCCATTGCCCAGCTATCTGTCACTTTATTGTGAAGATAGTGGAAAAGGAAGGTGGCTCCTACAAATGCCATCATTGCGATAAAGGAAAGGCCATCGTTGAAGATGCCTCTGCCGACAGTGGTCCCAAAGATGGACCCCCACCCACGAGGAGCATCGTGGAAAAAGAAGACGTTCCAACCACGTCTTCAAAGCAAGTGGATTGATGTGATAACATGGTGGAGCACGACACACTTGTCTACTCCAAAAATATCAAAGATACAGTCTCAGAAGACCAAAGGGCAATTGAGACTTTTCAACAAAGGGTAATATCCGGAAACCTCCTCGGATTCCATTGCCCAGCTATCTGTCACTTTATTGTGAAGATAGTGGAAAAGGAAGGTGGCTCCTACAAATGCCATCATTGCGATAAAGGAAAGGCCATCGTTGAAGATGCCTCTGCCGACAGTGGTCCCAAAGATGGACCCCCACCCACGAGGAGCATCGTGGAAAAAGAAGACGTTCCAACCACGTCTTCAAAGCAAGTGGATTGATGTGATATCTCCACTGACGTAAGGGATGACGCACAATCCCACTATCCTTCGCAAGACCCTTCCTCTATATAAGGAAGTTCATTTCATTTGGAGAGGACCTCGACCTCAACACAACATATACAAAACAAACGAATCTCAAGCAATCAAGCATTCTACTTCTATTGCAGCAATTTAAATCATTTCTTTTAAAGCAAAAGCAATTTTCTGAAAATTTTCACCATTTACGAACGATACTCGAGGGGGATCCCCAATACTTGTATGGCCGCGGCCGCTCTAGGCCTACTAGATGGATTACAAGGACCACGACGGGGATTACAAGGACCACGACATTGATTACAAGGATGATGATGACAAGATGGCTCCGAAGAAGAAGAGGAAGGTTGGCATCCACGGGGTGCCAGCTGCTGACAAGAAGTACTCGATCGGCCTCGATATTGGGACTAACTCTGTTGGCTGGGCCGTGATCACCGACGAGTACAAGGTGCCCTCAAAGAAGTTCAAGGTCCTGGGCAACACCGATCGGCATTCCATCAAGAAGAATCTCATTGGCGCTCTCCTGTTCGAC**AGA**GGCGAGACGGCTGAGGCTACGCGGCTCAAGCGCACCGCCCGCAGGCGGTACACGCGCAGGAAGAATCGCATCTGCTACCTGCAGGAGATTTTCTCCAACGAGATGGCGAAGGTTGACGATTCTTTCTTCCACAGGCTGGAGGAGTCATTCCTCGTGGAGGAGGATAAGAAGCACGAGCGGCATCCAATCTTCGGCAACATTGTCGACGAGGTTGCCTACCACGAGAAGTACCCTACGATCTACCATCTGCGGAAGAAGCTCGTGGACTCCACAGATAAGGCGGACCTCCGCCTGATCTACCTCGCTCTGGCCCACATGATTAAGTTCAGGGGCCATTTCCTGATCGAGGGGGATCTCAACCCGGACAATAGCGATGTTGACAAGCTGTTCATCCAGCTCGTGCAGACGTACAACCAGCTCTTCGAGGAGAACCCCATTAATGCGTCAGGCGTCGACGCGAAGGCTATCCTGTCCGCTAGGCTCTCGAAGTCTCGGCGCCTCGAGAACCTGATCGCCCAGCTGCCGGGCGAGAAGAAGAACGGCCTGTTCGGGAATCTCATTGCGCTCAGCCTGGGGCTCACGCCCAACTTCAAGTCGAATTTCGATCTCGCTGAGGACGCCAAGCTGCAGCTCTCCAAGGACACATACGACGATGACCTGGATAACCTCCTGGCCCAGATCGGCGATCAGTACGCGGACCTGTTCCTCGCTGCCAAGAATCTGTCGGACGCCATCCTCCTGTCTGATATTCTCAGGGTGAACACCGAGATTACGAAGGCTCCGCTCTCAGCCTCCATGATCAAGCGCTACGACGAGCACCATCAGGATCTGACCCTCCTGAAGGCGCTGGTCAGGCAGCAGCTCCCCGAGAAGTACAAGGAGATCTTCTTCGATCAGTCGAAGAACGGCTACGCTGGGTACATTGACGGCGGGGCCTCTCAGGAGGAGTTCTACAAGTTCATCAAGCCGATTCTGGAGAAGATGGACGGCACGGAGGAGCTGCTGGTGAAGCTCAATCGCGAGGACCTCCTGAGGAAGCAGCGGACATTCGATAACGGCAGCATCCCACACCAGATTCATCTCGGGGAGCTGCACGCTATCCTGAGGAGGCAGGAGGACTTCTACCCTTTCCTCAAGGATAACCGCGAGAAGATCGAGAAGATTCTGACTTTCAGGATCCCGTACTACGTCGGCCCACTCGCTAGGGGCAACTCCCGCTTCGCTTGGATGACCCGCAAGTCAGAGGAGACGATCACGCCGTGGAACTTCGAGGAGGTGGTCGACAAGGGCGCTAGCGCTCAGTCGTTCATCGAGAGGATGACGAATTTCGACAAGAACCTGCCAAATGAGAAGGTGCTCCCTAAGCACTCGCTCCTGTACGAGTACTTCACAGTCTACAACGAGCTGACTAAGGTGAAGTATGTGACCGAGGGCATGAGGAAGCCGGCTTTCCTGTCTGGGGAGCAGAAGAAGGCCATCGTGGACCTCCTGTTCAAGACCAACCGGAAGGTCACGGTTAAGCAGCTCAAGGAGGACTACTTCAAGAAGATTGAGTGCTTCGATTCGGTCGAGATCTCTGGCGTTGAGGACCGCTTCAACGCCTCCCTGGGGACCTACCACGATCTCCTGAAGATCATTAAGGATAAGGACTTCCTGGACAACGAGGAGAATGAGGATATCCTCGAGGACATTGTGCTGACACTCACTCTGTTCGAGGACCGGGAGATGATCGAGGAGCGCCTGAAGACTTACGCCCATCTCTTCGATGACAAGGTCATGAAGCAGCTCAAGAGGAGGAGGTACACCGGCTGGGGGAGGCTGAGCAGGAAGCTCATCAACGGCATTCGGGACAAGCAGTCCGGGAAGACGATCCTCGACTTCCTGAAGAGCGATGGCTTCGCGAACCGCAATTTCATGCAGCTGATTCACGATGACAGCCTCACATTCAAGGAGGATATCCAGAAGGCTCAGGTGAGCGGCCAGGGGGACTCGCTGCACGAGCATATCGCGAACCTCGCTGGCTCGCCAGCTATCAAGAAGGGGATTCTGCAGACCGTGAAGGTTGTGGACGAGCTGGTGAAGGTCATGGGCAGGCACAAGCCTGAGAACATCGTCATTGAGATGGCCCGGGAGAATCAGACCACGCAGAAGGGCCAGAAGAACTCACGCGAGAGGATGAAGAGGATCGAGGAGGGCATTAAGGAGCTGGGGTCCCAGATCCTCAAGGAGCACCCGGTGGAGAACACGCAGCTGCAGAATGAGAAGCTCTACCTGTACTACCTCCAGAATGGCCGCGATATGTATGTGGACCAGGAGCTGGATATTAACAGGCTCAGCGATTACGACGTCGATCATATCGTTCCACAGTCATTCCTGAAGGATGACTCCATTGACAACAAGGTCCTCACCAGGTCGGACAAGAACCGGGGCAAGTCTGATAATGTTCCTTCAGAGGAGGTCGTTAAGAAGATGAAGAACTACTGGCGCCAGCTCCTGAATGCCAAGCTGATCACGCAGCGGAAGTTCGATAACCTCACAAAGGCTGAGAGGGGCGGGCTCTCTGAGCTGGACAAGGCGGGCTTCATCAAGAGGCAGCTGGTCGAGACACGGCAGATCACTAAGCACGTTGCGCAGATTCTCGACTCACGGATGAACACTAAGTACGATGAGAATGACAAGCTGATCCGCGAGGTGAAGGTCATCACCCTGAAGTCAAAGCTCGTCTCCGACTTCAGGAAGGATTTCCAGTTCTACAAGGTT**GCG**GAGATCAACAATTACCACCATGCCCATGACGCGTACCTGAACGCGGTGGTCGGCACAGCTCTGATCAAGAAGTACCCA**GCG**CTCGAGAGCGAGTTCGTGTACGGGGACTACAAGGTTTACGATGTGAGGAAGATGATCGCCAAGTCGGAGCAGGAGATTGGCAAGGCTACCGCCAAGTACTTCTTCTACTCTAACATTATGAATTTCTTCAAGACAGAGATCACTCTGGCCAATGGCGAGATCCGGAAGCGCCCCCTCATCGAGACGAACGGCGAGACGGGGGAGATCGTGTGGGACAAGGGCAGGGATTTCGCGACCGTCAGGAAGGTTCTCTCCATGCCACAAGTGAATATCGTCAAGAAGACAGAGGTCCAGACTGGCGGGTTCTCTAAGGAGTCAATTCTGCCTAAGCGGAACAGCGACAAGCTCATCGCCCGCAAGAAGGACTGGGATCCGAAGAAGTACGGCGGGTTCGACAGCCCCACTGTGGCCTACTCGGTCCTGGTTGTGGCGAAGGTTGAGAAGGGCAAGTCCAAGAAGCTCAAGAGCGTGAAGGAGCTGCTGGGGATCACGATTATGGAGCGCTCCAGCTTCGAGAAGAACCCGATCGATTTCCTGGAGGCGAAGGGCTACAAGGAGGTGAAGAAGGACCTGATCATTAAGCTCCCCAAGTACTCACTCTTCGAGCTGGAGAACGGCAGGAAGCGGATGCTGGCTTCCGCTGGCGAGCTGCAGAAGGGGAACGAGCTGGCTCTGCCGTCCAAGTATGTGAACTTCCTCTACCTGGCCTCCCACTACGAGAAGCTCAAGGGCAGCCCCGAGGACAACGAGCAGAAGCAGCTGTTCGTCGAGCAGCACAAGCATTACCTCGACGAGATCATTGAGCAGATTTCCGAGTTCTCCAAGCGCGTGATCCTGGCCGACGCGAATCTGGATAAGGTCCTCTCCGCGTACAACAAGCACCGCGACAAGCCAATCAGGGAGCAGGCTGAGAATATCATTCATCTCTTC**CGC**CTGACGAACCTCGGCGCCCCTGCTGCTTTCAAGTACTTCGACACAACTATCGATCGCAAGAGGTACACAAGCACTAAGGAGGTCCTGGACGCGACCCTCATCCACCAGTCGATTACCGGCCTCTACGAGACGCGCATCGACCTGTCTCAGCTCGGGGGCGACAAGCGGCCAGCGGCGACGAAGAAGGCGGGGCAGGCGAAGAAGAAGAAGCTTAAGAGCGGAGGATCTTCCGGAGGATCTAGCGGCTCCGAGACACCAGGAACATCCGAAAGCGCTACACCAGAATCTAGCGGAGGCTCTTCCGGAGGATCTATGGGATCCAGTCTCGACGACGAGCACATCTTGTCCGCACTACTGCAATCCGACGACGAACTGGTCGGGGAAGACAGTGATTCTGAAGTAAGCGACCATGTCTCGGAGGACGATGTGCAGTCGGATACCGAAGAGGCCTTCATTGACGAGGTTCATGAGGTTCAGCCCACGTCTTCAGGATCTGAGATTCTTGATGAACAAAATGTTATTGAGCAGCCGGGCTCGAGCTTGGCTTCAAATAGAATTTTAACACTGCCTCAAAGGACAATCCGCGGCAAGAATAAGCACTGCTGGTCAACATCTAAACCAACGCGTCGCAGCCGCGTGTCTGCCTTAAATATAGTCAGGTCCCAGCGCGGGCCTACAAGGATGTGCAGAAACATTTATGATCCTCTTCTCTGCTTCAAGCTGTTCTTCACCGACGAGATAATCTCCGAGATCGTCAAGTGGACTAATGCTGAAATATCGCTCAAGCGTAGGGAGTCGATGACGTCCGCAACATTTAGGGATACGAATGAGGATGAAATTTATGCATTTTTTGGGATCCTGGTTATGACTGCTGTCAGGAAGGATAACCACATGTCAACTGATGACCTCTTTGATCGATCGTTGTCCATGGTGTATGTTTCCGTCATGAGTCGGGACAGGTTTGATTTCCTTATACGGTGTCTCCGCATGGATGACAAGAGCATACGACCAACATTAAGGGAAAATGACGTCTTCACGCCCGTTAGAAAGATATGGGACCTCTTCATTCATCAGTGCATTCAGAACTACACACCCGGCGCCCACTTGACCATTGATGAGCAACTTTTGGGTTTTCGCGGCAGATGTCCGTTCAGAGTGTACATCCCCAACAAGCCATCTAAGTACGGTATCAAAATACTGATGATGTGTGACTCGGGAACTAAATATATGATAAACGGCATGCCATATCTTGGGCGGGGTACTCAAACCAATGGTGTGCCACTAGGCGAGTACTATGTGAAAGAGCTGTCGAAGCCGGTCCACGGAAGCTGCCGCAACATCACCTGCGACAACTGGTTCACTTCGATCCCTCTCGCGAAGAACTTGCTACAGGAGCCTTACAAGCTCACGATCGTTGGAACTGTGGCCAGCAACGCACGTGAGATACCAGAAGTACTGAAAAATTCACGAAGTCGGCCCGTTGGCACTTCTATGTTCTGCTTTGACGGCCCGCTCACTCTCGTGTCTTATAAACCGAAGCCTGCCAAGATGGTTTATCTTTTATCCTCCTGTGATGAAGATGCATCCATCAATGAATCTACAGGAAAGCCACAAATGGTCATGTACTACAACCAAACAAAAGGGGGTGTCGACACTTTGAACCAGATGTGCAGCGTCATGACATGTTCAAGGAAAACCAACCGGTGGCCGATGGCCCTTCTCTACGGCATGATTAACATTGCCTGTATCAACTCCTTCATCATTTACTCTCACAATGTGTCATCAAAGGGAGAGAAGGTACAGAGTCGGAAGAAGTTCATGCGTAATCTCTACATGGGCCTTACCAGTAGCTTCATGAGAAAGAGACTGGAGGCGCCGACCCTAAAAAGGTACCTCAGGGATAATATCTCAAACATCTTGCCTAAGGAAGTACCAGGAACGTCAGATGATTCCACCGAGGAGCCTGTGATGAAGAAAAGGACCTACTGCACATATTGCCCGAGCAAAATACGCAGAAAGGCGTCGGCTTCATGTAAGAAGTGCAAGAAGGTGATTTGCCGGGAGCATAATATCGACATGTGCCAGTCTTGCTTCGCGCCCAAAAAAAAACGAAAAGTGGGCATTCATGGCGTGCCGGCGGCCTGAGCAGAGCTTTCGTTCGTATCATCGGTTTCGACAACGTTCGTCAAGTTCAATGCATCAGTTTCATTGCGCACACACCAGAATCCTACTGAGTTTGAGTATTATGGCATTGGGAAAACTGTTTTTCTTGTACCATTTGTTGTGCTTGTAATTTACTGTGTTTTTTATTCGGTTTTCGCTATCGAACTGTGAAATGGAAATGGATGGAGAAGAGTTAATGAATGATATGGTCCTTTTGTTCATTCTCAAATTAATATTATTTGTTTTTTCTCTTATTTGTTGTGTGTTGAATTTGAAATTATAAGAGATATGCAAACATTTTGTTTTGAGTAAAAATGTGTCAAATCGTGGCCTCTAATGACCGAAGTTAATATGAGGAGTAAAACACTTGTAGTTGTACCATTATGCTTATTCACTAGGCAACAAATATATTTTCAGACCTAGAAAAGCTGCAAATGTTACTGAATACAAGTATGTCCTCTTGTGTTTTAGACATTTATGAACTTTCCTTTATGTAATTTTCCAGAATCCTTGTCAGATTCTAATCATTGCTTTATAATTATAGTTATACTCATGGATTTGTAGTTGAGTATGAAAATATTTTTTAATGCATTTTATGACTTGCCAATTGATTGACAACGAATTCGTAATCATGTCATAGCTGTTTCCTGTGTGAAATTGTTATCCGCTCACAATTCCACACAACATACGAGCCGGAAGCATAAAGTGTAAAGCCTGGGGTGCCTAATGAGTGAGCTAACTCACATTAATTGCGTTGCGCTCACTGCCCGCTTTCCAGTCGGGAAACCTGTCGTGCCAGCTGCATTAATGAATCGGCCAACGCGCGGGGAGAGGCGGTTTGCGTATTGGCTAGAGCAGCTTGCCAACATGGTGGAGCACGACACTCTCGTCTACTCCAAGAATATCAAAGATACAGTCTCAGAAGACCAAAGGGCTATTGAGACTTTTCAACAAAGGGTAATATCGGGAAACCTCCTCGGATTCCATTGCCCAGCTATCTGTCACTTCATCAAAAGGACAGTAGAAAAGGAAGGTGGCACCTACAAATGCCATCATTGCGATAAAGGAAAGGCTATCGTTCAAGATGCCTCTGCCGACAGTGGTCCCAAAGATGGACCCCCACCCACGAGGAGCATCGTGGAAAAAGAAGACGTTCCAACCACGTCTTCAAAGCAAGTGGATTGATGTGAACATGGTGGAGCACGACACTCTCGTCTACTCCAAGAATATCAAAGATACAGTCTCAGAAGACCAAAGGGCTATTGAGACTTTTCAACAAAGGGTAATATCGGGAAACCTCCTCGGATTCCATTGCCCAGCTATCTGTCACTTCATCAAAAGGACAGTAGAAAAGGAAGGTGGCACCTACAAATGCCATCATTGCGATAAAGGAAAGGCTATCGTTCAAGATGCCTCTGCCGACAGTGGTCCCAAAGATGGACCCCCACCCACGAGGAGCATCGTGGAAAAAGAAGACGTTCCAACCACGTCTTCAAAGCAAGTGGATTGATGTGATATCTCCACTGACGTAAGGGATGACGCACAATCCCACTATCCTTCGCAAGACCCTTCCTCTATATAAGGAAGTTCATTTCATTTGGAGAGGACACGCTGAAATCACCAGTCTCTCTCTACAAATCTATCTCTCTCGAGCTTTCGCAGATCTGTCGATCGACCATGGGGATTGAACAAGATGGATTGCACGCAGGTTCTCCGGCCGCTTGGGTGGAGAGGCTATTCGGCTATGACTGGGCACAACAGACAATCGGCTGCTCTGATGCCGCCGTGTTTCGGCTGTCAGCGCAGGGGCGCCCGGTTCTTTTTGTCAAGACCGACCTGTCCGGTGCCCTGAATGAACTCCAGGACGAGGCAGCGCGGCTATCGTGGCTGGCCACGACGGGCGTTCCTTGCGCAGCTGTGCTCGACGTTGTCACTGAAGCGGGAAGGGACTGGCTGCTATTGGGCGAAGTGCCGGGGCAGGATCTCCTGTCATCTCACCTTGCTCCTGCCGAGAAAGTATCCATCATGGCTGATGCAATGCGGCGGCTGCATACGCTTGATCCGGCTACCTGCCCATTCGACCACCAAGCGAAACATCGCATCGAGCGAGCACGTACTCGGATGGAAGCCGGTCTTGTCGATCAGGATGATCTGGACGAAGAGCATCAGGGGCTCGCGCCAGCCGAACTGTTCGCCAGGCTCAAGGCGCGCATGCCCGACGGCGAGGATCTCGTCGTGACACATGGCGATGCCTGCTTGCCGAATATCATGGTGGAAAATGGCCGCTTTTCTGGATTCATCGACTGTGGCCGGCTGGGTGTGGCGGACCGCTATCAGGACATAGCGTTGGCTACCCGTGATATTGCTGAAGAGCTTGGCGGCGAATGGGCTGACCGCTTCCTCGTGCTTTACGGTATCGCCGCTCCCGATTCGCAGCGCATCGCCTTCTATCGCCTTCTTGACGAGTTCTTCTGAGCGGGACTCTGGGGTTCGGATCGATCCTCTAGCTAGAGTCGATCGACAAGCTCGAGTTTCTCCATAATAATGTGTGAGTAGTTCCCAGATAAGGGAATTAGGGTTCCTATAGGGTTTCGCTCATGTGTTGAGCATATAAGAAACCCTTAGTATGTATTTGTATTTGTAAAATACTTCTATCAATAAAATTTCTAATTCCTAAAACCAAAATCCAGTACTAAAATCCAGATCCCCCGAATTAATTCGGCGTTAATTCAGTACATTAAAAACGTCCGCAATGTGTTATTAAGTTGTCTAAGCGTCAATTTGTTTACACCACAATATATCCTGCCA

**pHUE-vCas9-ipB7^RKD^ (*OsAct*::*Gusi*)**

Right Border;Donor (sgRNA1-LE TIR-LHA-T2A-GOI-RHA-*U6*-RE TIR-sgRNA2); OsU3p-(Spacer1) gRNA scaffold-polyT-TaU3p-(Spacer2) gRNA scaffold-polyT; pUbi-3xFLAG-NLS-vCas9- NLS Linker-ipB7^RKD^- NLS; CaMV35s (enhanced)-Hyg; Left Border

GTTTACCCGCCAATATATCCTGTCAAACACTGATAGTTTTTGCAGATGTGGATTGCCAAGGTTAACCCTAGAAAGATAGTCTGCGTAAAATTGACGCATGGAATTCGATGAGATTTAGTTCTGCAATCTTCAATTGTCATACAGCAAGACTATATAATAGCTTTCAAAATAAAATCATAGGCAGTTCTCATAAATGGAATCATGTTTGAACATCCTAATTCTGTTGGCATGGAGTGCTTTGACATTTTGATGTGCTACAGTTGTGAATAACTGAATTTCCTTTTCCCAGGTATTGTGTTGGACTCTGGTGATGGTGTCAGCCACACTGTCCCCATCTATGAAGGATATGCTCTCCCCCATGCTATCCTTCGTCTCGACCTTGCTGGGCGTGATCTCACTGATTACCTCATGAAGATCCTGACGGAGCGTGGTTACTCATTCACCACAACGGCCGAGCGGGAAATTGTGAGGGACATGAAGGAGAAGCTTTCCTACATCGCCCTGGACTATGACCAGGAAATGGAGACTGCCAAGACCAGCTCCTCCGTGGAGAAGAGCTACGAGCTTCCTGATGGACAGGTTATCACCATTGGTGCTGAGCGTTTCCGCTGCCCTGAGGTCCTCTTCCAGCCTTCCTTCATAGGAATGGAAGCTGCGGGTATCCATGAGACTACATACAACTCCATCATGAAGTGCGACGTGGATATTAGGAAGGATCTATATGGCAACATCGTTCTCAGTGGTGGTACCACTATGTTCCCTGGCATTGCTGACAGGATGAGCAAGGAGATCACTGCCTTGGCTCCTAGCAGCATGAAGATCAAGGTGGTCGCCCCTCCTGAAAGGAAGTACAGTGTCTGGATTGGAGGATCCATCTTGGCATCTCTCAGCACATTCCAGCAGGTAAATATACAAATGCAGCAATGTAGTGTGTTTACCTCATGAACTTGATCAATTTGCTTACAATGTTGCTTGCCGTTGCAGATGTGGATTGCGAAAGCTGAGTACGACGAGTCTGGCCCATCTATCGTGCACAGGAAATGCTTCGAGGGCAGAGGAAGTCTTCTAACATGCGGTGACGTGGAGGAGAATCCCGGCCCTATGTTACGTCCTGTAGAAACCCCAACCCGTGAAATCAAAAAACTCGACGGCCTGTGGGCATTCAGTCTGGATCGCGAAAACTGTGGAATTGATCAGCGTTGGTGGGAAAGCGCGTTACAAGAAAGCCGGGCAATTGCTGTGCCAGGCAGTTTTAACGATCAGTTCGCCGATGCAGATATTCGTAATTATGCGGGCAACGTCTGGTATCAGCGCGAAGTCTTTATACCGAAAGGTAAGTCTTACTCTCTCTTTTTTGGTCTGTATTTTTAATTTTTTGAAGTATACTATTTGTACTGACGCTAATAATCTTTTTTCAGGTTGGGCAGGCCAGCGTATCGTGCTGCGTTTCGATGCGGTCACTCATTACGGCAAAGTGTGGGTCAATAATCAGGAAGTGATGGAGCATCAGGGCGGCTATACGCCATTTGAAGCCGATGTCACGCCGTATGTTATTGCCGGGAAAAGTGTACGTATCACCGTTTGTGTGAACAACGAACTGAACTGGCAGACTATCCCGCCGGGAATGGTGATTACCGACGAAAACGGCAAGAAAAAGCAGTCTTACTTCCATGATTTCTTTAACTATGCCGGAATCCATCGCAGCGTAATGCTCTACACCACGCCGAACACCTGGGTGGACGATATCACCGTGGTGACGCATGTCGCGCAAGACTGTAACCACGCGTCTGTTGACTGGCAGGTACTTCATGCTTCAACGTGTAACTTAAGAGATACTGTGTGAAATTTTATATTTCCATACATTTGCTTGACCTTTGCTTTTTGTCAATTTTTTTCCCCTTACAGGTGGTGGCCAATGGTGATGTCAGCGTTGAACTGCGTGATGCGGATCAACAGGTGGTTGCAACTGGACAAGGCACTAGCGGGACTTTGCAAGTGGTGAATCCGCACCTCTGGCAACCGGGTGAAGGTTATCTCTATGAACTGTGCGTCACAGCCAAAAGCCAGACAGAGTGTGATATCTACCCGCTTCGCGTCGGCATCCGGTCAGTGGCAGTGAAGGGCGAACAGTTCCTGATTAACCACAAACCGTTCTACTTTACTGGCTTTGGTCGTCATGAAGATGCGGACTTGCGTGGCAAAGGATTCGATAACGTGCTGATGGTGCACGACCACGCATTAATGGACTGGATTGGGGCCAACTCCTACCGTACCTCGCATTACCCTTACGCTGAAGAGATGCTCGACTGGGCAGATGAACATGGCATCGTGGTGATTGATGAAACTGCTGCTGTCGGCTTTAACCTCTCTTTAGGCATTGGTTTCGAAGCGGGCAACAAGCCGAAAGAACTGTACAGCGAAGAGGCAGTCAACGGGGAAACTCAGCAAGCGCACTTACAGGCGATTAAAGAGCTGATAGCGCGTGACAAAAACCACCCAAGCGTGGTGATGTGGAGTATTGCCAACGAACCGGATACCCGTCCGCAAGGTGCACGGGAATATTTCGCGCCACTGGCGGAAGCAACGCGTAAACTCGACCCGACGCGTCCGATCACCTGCGTCAATGTAATGTTCTGCGACGCTCACACCGATACCATCAGCGATCTCTTTGATGTGCTGTGCCTGAACCGTTATTACGGATGGTATGTCCAAAGCGGCGATTTGGAAACGGCAGAGAAGGTACTGGAAAAAGAACTTCTGGCCTGGCAGGAGAAACTGCATCAGCCGATTATCATCACCGAATACGGCGTGGATACGTTAGCCGGGCTGCACTCAATGTACACCGACATGTGGAGTGAAGAGTATCAGTGTGCATGGCTGGATATGTATCACCGCGTCTTTGATCGCGTCAGCGCCGTCGTCGGTGAACAGGTATGGAATTTCGCCGATTTTGCGACCTCGCAAGGCATATTGCGCGTTGGCGGTAACAAGAAAGGGATCTTCACTCGCGACCGCAAACCGAAGTCGGCGGCTTTTCTGCTGCAAAAACGCTGGACTGGCATGAACTTCGGTGAAAAACCGCAGCAGGGAGGCAAACAATGATTCTTCGGACCCAAGAATGCTAAGCCAAGAGGAGCTGTTATCGCCGTCCTCCTGCTTGTTTCTCTCTTTTTGTTGCTGTTTCTTCATTAGCGTGGACAAAGTTTTCAACCGGCCTATCTGTTATCATTTTCTTCTATTCAAAGACTGTAATACCTATTGCTACCTGTGGTTCTCACTTGTGATTTTGGACACATATGTTCGGTTTATTCAAATTTAATCAGATGCCTGATGAGGGTACCAGAAAAAATACGTGTTCTGGTTGTTTTTGAGTTGCGATTATTCTATGAAATGAATAACATCGAAGTTATCATCCCAGTATTTTCGCATGAATGTTCTTTTCTTCTGTCTTGTGCATCAGTGATCTAGTGCATGGGAGTTTGTATTGTGATGTTCGACATCACGTAACTTCCACTTTGCCTTTGCTGTTCGATATTTTAATGACATGTCACACACACTTCTGATACTTTTCTTTCTTGGCTATTGTGCCAGCATGATGCAAGATGCATCACAGCATCAGATATATTCTCATCGTCAGGCTTTAGCAGCACACGAGCACGCTTTGCCGCTTAAAAGTTGTACGGCGCAGCTTAGACATCCCCTGTAGAAGTGATAATCTTTTCACTTTTCCTTAAACAAATTGAGAGGGGAAATGGAACCATGTGGATCAGAGAAGCTTTTGTTTCTTTACACAAGAATATTTGGTACAGTGGGGGTCCTATGTTCGTGGGTTCGTGGCTTGGCTGCCTGTCTTCAACCAAGTGTTTTCAGTTCAACATGTTAGCGTGTAGAAAGAGCACAATTCTGTTTATCTCCAAGGTAAAATGTGGCATTCTGTTAAAGAACATGATCCTGCCAATTTTTTAAGTTTCAATGGAAGAGGAATGTAAAGCTTTCTATGGTTTGTGTACACAACACAGTGGAAGAGGAGTGCAAGCTTTCTATGGTTTGTGTGCGCGTTGTGTGGATCC*CAAGTCTGATGCAGCAAGCGAGTTTATAAACCGGTCAGAACTCTGCTGGACAGGCGAGGTGGGACTAAACTAAGAGGAACTGCTGCGTTGCTCTCCCCAATCGTGAACACGTCGACCACCCGACAAAGCCCGATCATGCGGGCCTAAAAGCCCAGCGCTAGACCGTTGCTGAACTCACGTGGGCTGCATCTCACTATGCGCCCAGATCAGTGAGAGCTAGAGGGCAGTTATCTTCAGCTCTCTCGTAGTTCCTCTGATCAATATTCCTAGTTCGATTCCCTCCTAGCAGTGAGTAACAATGTGCTCCTTGATAAATATAAATGTGTTGAGCACCAGAACTGTCAGAATAACGGGCTTGGTC*AAGCTTCCCTAGAAAGATAATCATATTGTGACGTACGTTAAAGATAATCATGCGTAAAATTGACGCATGTTAACCATTGTGCACAGGAAATGCTTAAACTGAAGGCGGGAAACGACAATCTGATCCAAGCTCAAGCTGCTCTAGCATTCGCCATTCAGGCTGCGCAACTGTTGGGAAGGGCGATCGGTGCGGGCCTCTTCGCTATTACGCCAGCTGGCGAAAGGGGGATGTGCTGCAAGGCGATTAAGTTGGGTAACGCCAGGGTTTTCCCAGTCACGACGTTGTAAAACGACGGCCAGTGCCAAGCTTAGTAATTCATCCAGGTCACCAAGTTCTAGGATTTTCAGAACTGCAACTTATTTTATCAAGGAATCTTTAAACATACGAACAGATCACTTAAAGTTCTTCTGAAGCAACTTAAAGTTATCAGGCATGCATGGATCTTGGAGGAATCAGATGTGCAGTCAGGGACCATAGCACAAGACAGGCGTCTTCTACTGGTGCTACCAGCAAATGCTGGAAGCCGGGAACACTGGGTACGTTGGAAACCACGTGATGTGAAGAAGTAAGATAAACTGTAGGAGAAAAGCATTTCGTAGTGGGCCATGAAGCCTTTCAGGACATGTATTGCAGTATGGGCCGGCCCATTACGCAATTGGACGACAACAAAGACTAGTATTAGTACCACCTCGGCTATCCACATAGATCAAAGCTGATTTAAAAGAGTTGTGCAGATGATCCGTGGCGTTGCAGATGTGGATTGCCAGTTTTAGAGCTAGAAATAGCAAGTTAAAATAAGGCTAGTCCGTTATCAACTTGAAAAAGTGGCACCGAGTCGGTGCTTTTTTTTTTCGTTTTGCATTGAGTTTTCTCCGTCGCATGTTTGCAGTTTTATTTTCCGTTTTGCATTGAAATTTCTCCGTCTCATGTTTGCAGCGTGTTCAAAAAGTACGCAGCTGTATTTCACTTATTTACGGCGCCACATTTTCATGCCGTTTGTGCCAACTATCCCGAGCTAGTGAATACAGCTTGGCTTCACACAACACTGGTGACCCGCTGACCTGCTCGTACCTCGTACCGTCGTACGGCACAGCATTTGGAATTAAAGGGTGTGATCGATACTGCTTGCTGCTCATGAATCCAAACCACACGGAGTTCAAATTCCCACAGATTAAGGCTCGTCCGTCGCACAAGGTAATGTGTGAATATTATATCTGTCGTGCAAAATTGCCTGGCCTGCACAATTGCTGTTATAGTTGGCGGCAGGGAGAGTTTTAACATTGACTAGCGTGCTGATAATTTGTGAGAAATAATAATTGACAAGTAGATACTGACATTTGAGAAGAGCTTCTGAACTGTTATTAGTAACAAAAATGGAAAGCTGATGCACGGAAAAAGGAAAGAAAAAGCCATACTTTTTTTTAGGTAGGAAAAGAAAAAGCCATACGAGACTGATGTCTCTCAGATGGGCCGGGATCTGTCTATCTAGCAGGCAGCAGCCCACCAACCTCACGGGCCAGCAATTACGAGTCCTTCTAAAAGCTCCCGCCGAGGGGCGCTGGCGCTGCTGTGCAGCAGCACGTCTAACATTAGTCCCACCTCGCCAGTTTACAGGGAGCAGAACCAGCTTATAAGCGGAGGCGCGGCACCAAGAAGCGAAGCATTTCCTGTGCACAAGTTTTAGAGCTAGAAATAGCAAGTTAAAATAAGGCTAGTCCGTTATCAACTTGAAAAAGTGGCACCGAGTCGGTGCTTTTTTTTTTCGTTTTGCATTGAGTTTTCTCCGTCGCATGTTTGCAGTTTTATTTTCCGTTTTGCATTGAAATTTCTCCGTCTCATGTTTGCAGCGTGTTCAAAAAGTACGCAGCTGTATTTCACTTATTTACGGCGCCACATTTTCATGCCGTTTGTGCCAACTATCCCGAGCTAGTGAATACAGCTTGGCTTCACACAACACTGGTGACCCGCTGACCTGCTCGTACCTCGTACCGTCGTACGGCACAGCATTTGGAATTAAAGGGTGTGATCGATACTGCTTGCTGCTAAGCTTGCATGCCTGCAGTGCAGCGTGACCCGGTCGTGCCCCTCTCTAGAGATAATGAGCATTGCATGTCTAAGTTATAAAAAATTACCACATATTTTTTTTGTCACACTTGTTTGAAGTGCAGTTTATCTATCTTTATACATATATTTAAACTTTACTCTACGAATAATATAATCTATAGTACTACAATAATATCAGTGTTTTAGAGAATCATATAAATGAACAGTTAGACATGGTCTAAAGGACAATTGAGTATTTTGACAACAGGACTCTACAGTTTTATCTTTTTAGTGTGCATGTGTTCTCCTTTTTTTTTGCAAATAGCTTCACCTATATAATACTTCATCCATTTTATTAGTACATCCATTTAGGGTTTAGGGTTAATGGTTTTTATAGACTAATTTTTTTAGTACATCTATTTTATTCTATTTTAGCCTCTAAATTAAGAAAACTAAAACTCTATTTTAGTTTTTTTATTTAATAATTTAGATATAAAATAGAATAAAATAAAGTGACTAAAAATTAAACAAATACCCTTTAAGAAATTAAAAAAACTAAGGAAACATTTTTCTTGTTTCGAGTAGATAATGCCAGCCTGTTAAACGCCGTCGACGAGTCTAACGGACACCAACCAGCGAACCAGCAGCGTCGCGTCGGGCCAAGCGAAGCAGACGGCACGGCATCTCTGTCGCTGCCTCTGGACCCCTCTCGAGAGTTCCGCTCCACCGTTGGACTTGCTCCGCTGTCGGCATCCAGAAATTGCGTGGCGGAGCGGCAGACGTGAGCCGGCACGGCAGGCGGCCTCCTCCTCCTCTCACGGCACGGCAGCTACGGGGGATTCCTTTCCCACCGCTCCTTCGCTTTCCCTTCCTCGCCCGCCGTAATAAATAGACACCCCCTCCACACCCTCTTTCCCCAACCTCGTGTTGTTCGGAGCGCACACACACACAACCAGATCTCCCCCAAATCCACCCGTCGGCACCTCCGCTTCAAGGTACGCCGCTCGTCCTCCCCCCCCCCCCCTCTCTACCTTCTCTAGATCGGCGTTCCGGTCCATGGTTAGGGCCCGGTAGTTCTACTTCTGTTCATGTTTGTGTTAGATCCGTGTTTGTGTTAGATCCGTGCTGCTAGCGTTCGTACACGGATGCGACCTGTACGTCAGACACGTTCTGATTGCTAACTTGCCAGTGTTTCTCTTTGGGGAATCCTGGGATGGCTCTAGCCGTTCCGCAGACGGGATCGATTTCATGATTTTTTTTGTTTCGTTGCATAGGGTTTGGTTTGCCCTTTTCCTTTATTTCAATATATGCCGTGCACTTGTTTGTCGGGTCATCTTTTCATGCTTTTTTTTGTCTTGGTTGTGATGATGTGGTCTGGTTGGGCGGTCGTTCTAGATCGGAGTAGAATTCTGTTTCAAACTACCTGGTGGATTTATTAATTTTGGATCTGTATGTGTGTGCCATACATATTCATAGTTACGAATTGAAGATGATGGATGGAAATATCGATCTAGGATAGGTATACATGTTGATGCGGGTTTTACTGATGCATATACAGAGATGCTTTTTGTTCGCTTGGTTGTGATGATGTGGTGTGGTTGGGCGGTCGTTCATTCGTTCTAGATCGGAGTAGAATACTGTTTCAAACTACCTGGTGTATTTATTAATTTTGGAACTGTATGTGTGTGTCATACATCTTCATAGTTACGAGTTTAAGATGGATGGAAATATCGATCTAGGATAGGTATACATGTTGATGTGGGTTTTACTGATGCATATACATGATGGCATATGCAGCATCTATTCATATGCTCTAACCTTGAGTACCTATCTATTATAATAAACAAGTATGTTTTATAATTATTTTGATCTTGATATACTTGGATGATGGCATATGCAGCAGCTATATGTGGATTTTTTTAGCCCTGCCTTCATACGCTATTTATTTGCTTGGTACTGTTTCTTTTGTCGATGCTCACCCTGTTGTTTGGTGTTACTTCTGCAGCCCTAGGCCTACTAGATGGATTACAAGGACCACGACGGGGATTACAAGGACCACGACATTGATTACAAGGATGATGATGACAAGATGGCTCCGAAGAAGAAGAGGAAGGTTGGCATCCACGGGGTGCCAGCTGCTGACAAGAAGTACTCGATCGGCCTCGATATTGGGACTAACTCTGTTGGCTGGGCCGTGATCACCGACGAGTACAAGGTGCCCTCAAAGAAGTTCAAGGTCCTGGGCAACACCGATCGGCATTCCATCAAGAAGAATCTCATTGGCGCTCTCCTGTTCGAC**AGA**GGCGAGACGGCTGAGGCTACGCGGCTCAAGCGCACCGCCCGCAGGCGGTACACGCGCAGGAAGAATCGCATCTGCTACCTGCAGGAGATTTTCTCCAACGAGATGGCGAAGGTTGACGATTCTTTCTTCCACAGGCTGGAGGAGTCATTCCTCGTGGAGGAGGATAAGAAGCACGAGCGGCATCCAATCTTCGGCAACATTGTCGACGAGGTTGCCTACCACGAGAAGTACCCTACGATCTACCATCTGCGGAAGAAGCTCGTGGACTCCACAGATAAGGCGGACCTCCGCCTGATCTACCTCGCTCTGGCCCACATGATTAAGTTCAGGGGCCATTTCCTGATCGAGGGGGATCTCAACCCGGACAATAGCGATGTTGACAAGCTGTTCATCCAGCTCGTGCAGACGTACAACCAGCTCTTCGAGGAGAACCCCATTAATGCGTCAGGCGTCGACGCGAAGGCTATCCTGTCCGCTAGGCTCTCGAAGTCTCGGCGCCTCGAGAACCTGATCGCCCAGCTGCCGGGCGAGAAGAAGAACGGCCTGTTCGGGAATCTCATTGCGCTCAGCCTGGGGCTCACGCCCAACTTCAAGTCGAATTTCGATCTCGCTGAGGACGCCAAGCTGCAGCTCTCCAAGGACACATACGACGATGACCTGGATAACCTCCTGGCCCAGATCGGCGATCAGTACGCGGACCTGTTCCTCGCTGCCAAGAATCTGTCGGACGCCATCCTCCTGTCTGATATTCTCAGGGTGAACACCGAGATTACGAAGGCTCCGCTCTCAGCCTCCATGATCAAGCGCTACGACGAGCACCATCAGGATCTGACCCTCCTGAAGGCGCTGGTCAGGCAGCAGCTCCCCGAGAAGTACAAGGAGATCTTCTTCGATCAGTCGAAGAACGGCTACGCTGGGTACATTGACGGCGGGGCCTCTCAGGAGGAGTTCTACAAGTTCATCAAGCCGATTCTGGAGAAGATGGACGGCACGGAGGAGCTGCTGGTGAAGCTCAATCGCGAGGACCTCCTGAGGAAGCAGCGGACATTCGATAACGGCAGCATCCCACACCAGATTCATCTCGGGGAGCTGCACGCTATCCTGAGGAGGCAGGAGGACTTCTACCCTTTCCTCAAGGATAACCGCGAGAAGATCGAGAAGATTCTGACTTTCAGGATCCCGTACTACGTCGGCCCACTCGCTAGGGGCAACTCCCGCTTCGCTTGGATGACCCGCAAGTCAGAGGAGACGATCACGCCGTGGAACTTCGAGGAGGTGGTCGACAAGGGCGCTAGCGCTCAGTCGTTCATCGAGAGGATGACGAATTTCGACAAGAACCTGCCAAATGAGAAGGTGCTCCCTAAGCACTCGCTCCTGTACGAGTACTTCACAGTCTACAACGAGCTGACTAAGGTGAAGTATGTGACCGAGGGCATGAGGAAGCCGGCTTTCCTGTCTGGGGAGCAGAAGAAGGCCATCGTGGACCTCCTGTTCAAGACCAACCGGAAGGTCACGGTTAAGCAGCTCAAGGAGGACTACTTCAAGAAGATTGAGTGCTTCGATTCGGTCGAGATCTCTGGCGTTGAGGACCGCTTCAACGCCTCCCTGGGGACCTACCACGATCTCCTGAAGATCATTAAGGATAAGGACTTCCTGGACAACGAGGAGAATGAGGATATCCTCGAGGACATTGTGCTGACACTCACTCTGTTCGAGGACCGGGAGATGATCGAGGAGCGCCTGAAGACTTACGCCCATCTCTTCGATGACAAGGTCATGAAGCAGCTCAAGAGGAGGAGGTACACCGGCTGGGGGAGGCTGAGCAGGAAGCTCATCAACGGCATTCGGGACAAGCAGTCCGGGAAGACGATCCTCGACTTCCTGAAGAGCGATGGCTTCGCGAACCGCAATTTCATGCAGCTGATTCACGATGACAGCCTCACATTCAAGGAGGATATCCAGAAGGCTCAGGTGAGCGGCCAGGGGGACTCGCTGCACGAGCATATCGCGAACCTCGCTGGCTCGCCAGCTATCAAGAAGGGGATTCTGCAGACCGTGAAGGTTGTGGACGAGCTGGTGAAGGTCATGGGCAGGCACAAGCCTGAGAACATCGTCATTGAGATGGCCCGGGAGAATCAGACCACGCAGAAGGGCCAGAAGAACTCACGCGAGAGGATGAAGAGGATCGAGGAGGGCATTAAGGAGCTGGGGTCCCAGATCCTCAAGGAGCACCCGGTGGAGAACACGCAGCTGCAGAATGAGAAGCTCTACCTGTACTACCTCCAGAATGGCCGCGATATGTATGTGGACCAGGAGCTGGATATTAACAGGCTCAGCGATTACGACGTCGATCATATCGTTCCACAGTCATTCCTGAAGGATGACTCCATTGACAACAAGGTCCTCACCAGGTCGGACAAGAACCGGGGCAAGTCTGATAATGTTCCTTCAGAGGAGGTCGTTAAGAAGATGAAGAACTACTGGCGCCAGCTCCTGAATGCCAAGCTGATCACGCAGCGGAAGTTCGATAACCTCACAAAGGCTGAGAGGGGCGGGCTCTCTGAGCTGGACAAGGCGGGCTTCATCAAGAGGCAGCTGGTCGAGACACGGCAGATCACTAAGCACGTTGCGCAGATTCTCGACTCACGGATGAACACTAAGTACGATGAGAATGACAAGCTGATCCGCGAGGTGAAGGTCATCACCCTGAAGTCAAAGCTCGTCTCCGACTTCAGGAAGGATTTCCAGTTCTACAAGGTT**GCG**GAGATCAACAATTACCACCATGCCCATGACGCGTACCTGAACGCGGTGGTCGGCACAGCTCTGATCAAGAAGTACCCA**GCG**CTCGAGAGCGAGTTCGTGTACGGGGACTACAAGGTTTACGATGTGAGGAAGATGATCGCCAAGTCGGAGCAGGAGATTGGCAAGGCTACCGCCAAGTACTTCTTCTACTCTAACATTATGAATTTCTTCAAGACAGAGATCACTCTGGCCAATGGCGAGATCCGGAAGCGCCCCCTCATCGAGACGAACGGCGAGACGGGGGAGATCGTGTGGGACAAGGGCAGGGATTTCGCGACCGTCAGGAAGGTTCTCTCCATGCCACAAGTGAATATCGTCAAGAAGACAGAGGTCCAGACTGGCGGGTTCTCTAAGGAGTCAATTCTGCCTAAGCGGAACAGCGACAAGCTCATCGCCCGCAAGAAGGACTGGGATCCGAAGAAGTACGGCGGGTTCGACAGCCCCACTGTGGCCTACTCGGTCCTGGTTGTGGCGAAGGTTGAGAAGGGCAAGTCCAAGAAGCTCAAGAGCGTGAAGGAGCTGCTGGGGATCACGATTATGGAGCGCTCCAGCTTCGAGAAGAACCCGATCGATTTCCTGGAGGCGAAGGGCTACAAGGAGGTGAAGAAGGACCTGATCATTAAGCTCCCCAAGTACTCACTCTTCGAGCTGGAGAACGGCAGGAAGCGGATGCTGGCTTCCGCTGGCGAGCTGCAGAAGGGGAACGAGCTGGCTCTGCCGTCCAAGTATGTGAACTTCCTCTACCTGGCCTCCCACTACGAGAAGCTCAAGGGCAGCCCCGAGGACAACGAGCAGAAGCAGCTGTTCGTCGAGCAGCACAAGCATTACCTCGACGAGATCATTGAGCAGATTTCCGAGTTCTCCAAGCGCGTGATCCTGGCCGACGCGAATCTGGATAAGGTCCTCTCCGCGTACAACAAGCACCGCGACAAGCCAATCAGGGAGCAGGCTGAGAATATCATTCATCTCTTC**CGC**CTGACGAACCTCGGCGCCCCTGCTGCTTTCAAGTACTTCGACACAACTATCGATCGCAAGAGGTACACAAGCACTAAGGAGGTCCTGGACGCGACCCTCATCCACCAGTCGATTACCGGCCTCTACGAGACGCGCATCGACCTGTCTCAGCTCGGGGGCGACAAGCGGCCAGCGGCGACGAAGAAGGCGGGGCAGGCGAAGAAGAAGAAGCTTAAGAGCGGAGGATCTTCCGGAGGATCTAGCGGCTCCGAGACACCAGGAACATCCGAAAGCGCTACACCAGAATCTAGCGGAGGCTCTTCCGGAGGATCTATGGGATCCAGTCTCGACGACGAGCACATCTTGTCCGCACTACTGCAATCCGACGACGAACTGGTCGGGGAAGACAGTGATTCTGAAGTAAGCGACCATGTCTCGGAGGACGATGTGCAGTCGGATACCGAAGAGGCCTTCATTGACGAGGTTCATGAGGTTCAGCCCACGTCTTCAGGATCTGAGATTCTTGATGAACAAAATGTTATTGAGCAGCCGGGCTCGAGCTTGGCTTCAAATAGAATTTTAACACTGCCTCAAAGGACAATCCGCGGCAAGAATAAGCACTGCTGGTCAACATCTAAACCAACGCGTCGCAGCCGCGTGTCTGCCTTAAATATAGTCAGGTCCCAGCGCGGGCCTACAAGGATGTGCAGAAACATTTATGATCCTCTTCTCTGCTTCAAGCTGTTCTTCACCGACGAGATAATCTCCGAGATCGTCAAGTGGACTAATGCTGAAATATCGCTCAAGCGTAGGGAGTCGATGACGTCCGCAACATTTAGGGATACGAATGAGGATGAAATTTATGCATTTTTTGGGATCCTGGTTATGACTGCTGTCAGGAAGGATAACCACATGTCAACTGATGACCTCTTTGATCGATCGTTGTCCATGGTGTATGTTTCCGTCATGAGTCGGGACAGGTTTGATTTCCTTATACGGTGTCTCCGCATGGATGACAAGAGCATACGACCAACATTAAGGGAAAATGACGTCTTCACGCCCGTTAGAAAGATATGGGACCTCTTCATTCATCAGTGCATTCAGAACTACACACCCGGCGCCCACTTGACCATTGATGAGCAACTTTTGGGTTTTCGCGGCAGATGTCCGTTCAGAGTGTACATCCCCAACAAGCCATCTAAGTACGGTATCAAAATACTGATGATGTGTGACTCGGGAACTAAATATATGATAAACGGCATGCCATATCTTGGGCGGGGTACTCAAACCAATGGTGTGCCACTAGGCGAGTACTATGTGAAAGAGCTGTCGAAGCCGGTCCACGGAAGCTGCCGCAACATCACCTGCGACAACTGGTTCACTTCGATCCCTCTCGCGAAGAACTTGCTACAGGAGCCTTACAAGCTCACGATCGTTGGAACTGTGGCCAGCAACGCACGTGAGATACCAGAAGTACTGAAAAATTCACGAAGTCGGCCCGTTGGCACTTCTATGTTCTGCTTTGACGGCCCGCTCACTCTCGTGTCTTATAAACCGAAGCCTGCCAAGATGGTTTATCTTTTATCCTCCTGTGATGAAGATGCATCCATCAATGAATCTACAGGAAAGCCACAAATGGTCATGTACTACAACCAAACAAAAGGGGGTGTCGACACTTTGAACCAGATGTGCAGCGTCATGACATGTTCAAGGAAAACCAACCGGTGGCCGATGGCCCTTCTCTACGGCATGATTAACATTGCCTGTATCAACTCCTTCATCATTTACTCTCACAATGTGTCATCAAAGGGAGAGAAGGTACAGAGTCGGAAGAAGTTCATGCGTAATCTCTACATGGGCCTTACCAGTAGCTTCATGAGAAAGAGACTGGAGGCGCCGACCCTAAAAAGGTACCTCAGGGATAATATCTCAAACATCTTGCCTAAGGAAGTACCAGGAACGTCAGATGATTCCACCGAGGAGCCTGTGATGAAGAAAAGGACCTACTGCACATATTGCCCGAGCAAAATACGCAGAAAGGCGTCGGCTTCATGTAAGAAGTGCAAGAAGGTGATTTGCCGGGAGCATAATATCGACATGTGCCAGTCTTGCTTCGCGCCCAAAAAAAAACGAAAAGTGGGCATTCATGGCGTGCCGGCGGCCTGAGAGCTCAGAGCTTTCGTTCGTATCATCGGTTTCGACAACGTTCGTCAAGTTCAATGCATCAGTTTCATTGCGCACACACCAGAATCCTACTGAGTTTGAGTATTATGGCATTGGGAAAACTGTTTTTCTTGTACCATTTGTTGTGCTTGTAATTTACTGTGTTTTTTATTCGGTTTTCGCTATCGAACTGTGAAATGGAAATGGATGGAGAAGAGTTAATGAATGATATGGTCCTTTTGTTCATTCTCAAATTAATATTATTTGTTTTTTCTCTTATTTGTTGTGTGTTGAATTTGAAATTATAAGAGATATGCAAACATTTTGTTTTGAGTAAAAATGTGTCAAATCGTGGCCTCTAATGACCGAAGTTAATATGAGGAGTAAAACACTTGTAGTTGTACCATTATGCTTATTCACTAGGCAACAAATATATTTTCAGACCTAGAAAAGCTGCAAATGTTACTGAATACAAGTATGTCCTCTTGTGTTTTAGACATTTATGAACTTTCCTTTATGTAATTTTCCAGAATCCTTGTCAGATTCTAATCATTGCTTTATAATTATAGTTATACTCATGGATTTGTAGTTGAGTATGAAAATATTTTTTAATGCATTTTATGACTTGCCAATTGATTGACAACGAATTCGTAATCATGTCATAGCTGTTTCCTGTGTGAAATTGTTATCCGCTCACAATTCCACACAACATACGAGCCGGAAGCATAAAGTGTAAAGCCTGGGGTGCCTAATGAGTGAGCTAACTCACATTAATTGCGTTGCGCTCACTGCCCGCTTTCCAGTCGGGAAACCTGTCGTGCCAGCTGCATTAATGAATCGGCCAACGCGCGGGGAGAGGCGGTTTGCGTATTGGCTAGAGCAGCTTGCCAACATGGTGGAGCACGACACTCTCGTCTACTCCAAGAATATCAAAGATACAGTCTCAGAAGACCAAAGGGCTATTGAGACTTTTCAACAAAGGGTAATATCGGGAAACCTCCTCGGATTCCATTGCCCAGCTATCTGTCACTTCATCAAAAGGACAGTAGAAAAGGAAGGTGGCACCTACAAATGCCATCATTGCGATAAAGGAAAGGCTATCGTTCAAGATGCCTCTGCCGACAGTGGTCCCAAAGATGGACCCCCACCCACGAGGAGCATCGTGGAAAAAGAAGACGTTCCAACCACGTCTTCAAAGCAAGTGGATTGATGTGATAACATGGTGGAGCACGACACTCTCGTCTACTCCAAGAATATCAAAGATACAGTCTCAGAAGACCAAAGGGCTATTGAGACTTTTCAACAAAGGGTAATATCGGGAAACCTCCTCGGATTCCATTGCCCAGCTATCTGTCACTTCATCAAAAGGACAGTAGAAAAGGAAGGTGGCACCTACAAATGCCATCATTGCGATAAAGGAAAGGCTATCGTTCAAGATGCCTCTGCCGACAGTGGTCCCAAAGATGGACCCCCACCCACGAGGAGCATCGTGGAAAAAGAAGACGTTCCAACCACGTCTTCAAAGCAAGTGGATTGATGTGATATCTCCACTGACGTAAGGGATGACGCACAATCCCACTATCCTTCGCAAGACCTTCCTCTATATAAGGAAGTTCATTTCATTTGGAGAGGACACGCTGAAATCACCAGTCTCTCTCTACAAATCTATCTCTCTCGAGCTTTCGCAGATCCCGGGGGGCAATGAGATATGAAAAAGCCTGAACTCACCGCGACGTCTGTCGAGAAGTTTCTGATCGAAAAGTTCGACAGCGTCTCCGACCTGATGCAGCTCTCGGAGGGCGAAGAATCTCGTGCTTTCAGCTTCGATGTAGGAGGGCGTGGATATGTCCTGCGGGTAAATAGCTGCGCCGATGGTTTCTACAAAGATCGTTATGTTTATCGGCACTTTGCATCGGCCGCGCTCCCGATTCCGGAAGTGCTTGACATTGGGGAGTTTAGCGAGAGCCTGACCTATTGCATCTCCCGCCGTGCACAGGGTGTCACGTTGCAAGACCTGCCTGAAACCGAACTGCCCGCTGTTCTACAACCGGTCGCGGAGGCTATGGATGCGATCGCTGCGGCCGATCTTAGCCAGACGAGCGGGTTCGGCCCATTCGGACCGCAAGGAATCGGTCAATACACTACATGGCGTGATTTCATATGCGCGATTGCTGATCCCCATGTGTATCACTGGCAAACTGTGATGGACGACACCGTCAGTGCGTCCGTCGCGCAGGCTCTCGATGAGCTGATGCTTTGGGCCGAGGACTGCCCCGAAGTCCGGCACCTCGTGCACGCGGATTTCGGCTCCAACAATGTCCTGACGGACAATGGCCGCATAACAGCGGTCATTGACTGGAGCGAGGCGATGTTCGGGGATTCCCAATACGAGGTCGCCAACATCTTCTTCTGGAGGCCGTGGTTGGCTTGTATGGAGCAGCAGACGCGCTACTTCGAGCGGAGGCATCCGGAGCTTGCAGGATCGCCACGACTCCGGGCGTATATGCTCCGCATTGGTCTTGACCAACTCTATCAGAGCTTGGTTGACGGCAATTTCGATGATGCAGCTTGGGCGCAGGGTCGATGCGACGCAATCGTCCGATCCGGAGCCGGGACTGTCGGGCGTACACAAATCGCCCGCAGAAGCGCGGCCGTCTGGACCGATGGCTGTGTAGAAGTACTCGCCGATAGTGGAAACCGACGCCCCAGCACTCGTCCGAGGGCAAAGAAATAGAGTAGATGCCGACCGGGATCTGTCGATCGACAAGCTCGAGTTTCTCCATAATAATGTGTGAGTAGTTCCCAGATAAGGGAATTAGGGTTCCTATAGGGTTTCGCTCATGTGTTGAGCATATAAGAAACCCTTAGTATGTATTTGTATTTGTAAAATACTTCTATCAATAAAATTTCTAATTCCTAAAACCAAAATCCAGTACTAAAATCCAGATCCCCCGAATTAATTCGGCGTTAATTCAGTACATTAAAAACGTCCGCAATGTGTTATTAAGTTGTCTAAGCGTCAATTTGTTTACACCACAATATATCCTGCCA
